# Prediction of Mechanosensitive Genes in Vascular Endothelial Cells Under High Wall Shear Stress

**DOI:** 10.3389/fgene.2021.796812

**Published:** 2022-01-11

**Authors:** Lei Shen, Kaige Zhou, Hong Liu, Jie Yang, Shuqi Huang, Fei Yu, Dongya Huang

**Affiliations:** ^1^ Department of Neurology, Shanghai East Hospital, School of Medicine, Tongji University, Shanghai, China; ^2^ School of Medicine, Tongji University, Shanghai, China; ^3^ Department of Neurology, Shanghai Tianyou Hospital, School of Medicine, Tongji University, Shanghai, China

**Keywords:** mechanosensitive genes, wall shear stress, vascular endothelial cells, atherosclerotic plaques, ischemic stroke

## Abstract

**Objective:** The vulnerability of atherosclerotic plaques is among the leading cause of ischemic stroke. High wall shear stress (WSS) promotes the instability of atherosclerotic plaques by directly imparting mechanical stimuli, but the specific mechanisms remain unclear. We speculate that modulation of mechanosensitive genes may play a vital role in accelerating the development of plaques. The purpose of this study was to find mechanosensitive genes in vascular endothelial cells (ECs) through combining microarray data with bioinformatics technology and further explore the underlying dynamics–related mechanisms that cause the progression and destabilization of atherosclerotic plaques.

**Methods:** Microarray data sets for human vascular ECs under high and normal WSS were retrieved from the Gene Expression Omnibus (GEO) database. Differentially expressed genes (DEGs) were identified through the R language. The performance of enrichment analysis and protein–protein interaction (PPI) network presented the biological function and signaling pathways of the DEGs. Hub genes were identified based on the PPI network and validated by GEO data sets. Predicted transcription factor (TF) genes and miRNAs interaction with potential mechanosensitive genes were identified by NetworkAnalyst.

**Results:** A total of 260 DEGs, 121 upregulated and 139 downregulated genes, were screened between high and normal WSS from GSE23289. A total of 10 hub genes and four cluster modules were filtered out based on the PPI network. The enrichment analysis showed that the biological functions of the hub genes were mainly involved in responses to unfolded protein and topologically incorrect protein, and t to endoplasmic reticulum stress. The significant pathways associated with the hub genes were those of protein processing in the endoplasmic reticulum, antigen processing, and presentation. Three out of the 10 hub genes, namely, activated transcription factor 3 (ATF3), heat shock protein family A (Hsp70) member 6 (HSPA6), and dual specificity phosphatase 1 (DUSP1, also known as CL100, HVH1, MKP-1, PTPN10), were verified in GSE13712. The expression of DUSP1 was higher in the senescent cell under high WSS than that of the young cell. The TF–miRNA–mechanosensitive gene coregulatory network was constructed.

**Conclusion:** In this work, we identified three hub genes, ATF3, HSPA6, and DUSP1, as the potential mechanosensitive genes in the human blood vessels. DUSP1 was confirmed to be associated with the senescence of vascular ECs. Therefore, these three mechanosensitive genes may have emerged as potential novel targets for the prediction and prevention of ischemic stroke. Furthermore, the TF–miRNA–mechanosensitive genes coregulatory network reveals an underlying regulatory mechanism and the pathways to control disease progression.

## Introduction

Atherosclerosis (AS), the underlying cause of ischemic stroke, is the most common cause of morbidity, mortality, and disability in the world, which has become a major global health problem due to the lack of effective methods for screening at an early stage and limited therapeutic strategies. This disease imposes serious economic burden and health toll on the individuals, families, and health care systems indirectly (Hoshino et al., 2018; Geng et al., 2019). Atherosclerosis is a chronic inflammatory disorder of the arterial wall (Kimura et al., 2018). The aggressive control of the several risk factors for atherosclerosis, including smoking, hypertension, diabetes, and hyperlipidemia, or any other form of stroke is still nearly 14.9–24% (Derdeyn et al., 2014). It has been therefore suggested that various additional factors and pathogenic mechanisms may be involved, which urges physicians and biomedical scientists to find more effective approaches to diagnose, prevent, and treat this deadly disease.

The vascular endothelial cells (ECs) line the inner layer of the blood vessels, constituting a barrier that separates the blood from the vessel wall under normal physiological conditions (Pulous et al., 2019). Endothelial dysfunction increases endothelial permeability and stimulates a complex series of reactions such as lipid accumulation, inflammatory responses, neovascularization, unstable plaque progression, and oxidative stress to promote the development and progression of atherosclerosis (Zhang et al., 2015a; Badimon et al., 2017; Wiese et al., 2019). Numerous researches have shown that atherosclerotic lesions preferentially develop in areas of arterial turns or bifurcation where hemodynamic wall shear stress (WSS) is low, oscillatory, or multidirectional and complex, also known as disturbed flow (Malek et al., 1999; Ramírez et al., 2019). The pattern of complex blood flow and WSS in the vessels are thought to be associated with endothelial dysfunction, which may result in changes in endothelial cellular function by directly affecting WSS or regulating the expression of genes of ECs in the different regions of the arterial wall (Casey et al., 2012; Mack et al., 2017). Recent molecular and cellular studies have begun to elucidate that low shear stress acting directly on vascular ECs leads to the formation of atherosclerosis and the development of thin fibrous caps. It promotes the deposition of particulate matter on the arterial wall and the accumulation of plaque, resulting from persistent changes of inflammation, oxidative stress, and proliferation in ECs (Feldman et al., 2002; Chatzizisis et al., 2007; Milkiewicz et al., 2008; Hsu et al., 2019). Conversely, high WSS was not only more commonly associated with a positive remodeling but also induced inflammation and increased oxidative stress by directly acting on the plaques, making the atherosclerotic plaques destabilized and causing embolism (Cicha et al., 2011; Costopoulos et al., 2019; Leng et al., 2019). The vascular endothelial function is regulated by WSS through some shear-sensitive expressions of the genes, such as KLF2/KLF4, NRF2, HIF-1α, NF-κB, etc. (Spruell and Baker, 2013; Wang et al., 2016; Niu et al., 2019). However, the molecular mechanisms involved in this process remain largely unknown. The discovery of novel mechanosensitive genes and their potential functional mechanisms in ECs have great implications in the individualized early diagnosis and precision treatment of ischemic stroke.

Currently, genomic sequencing and microarray technology have been widely applied by numerous laboratories around the world in various biological problems and disease mechanisms to identify new biomarkers. Through bioinformatics analysis, it is possible to obtain tremendous gene expression information and explore disease-associated genes and their biological functions, processes, as well as signaling pathways. Furthermore, bioinformatics analysis plays an important role in molecular genetics and also provides a theoretical basis for “precision medicine.”

**FIGURE 1:**
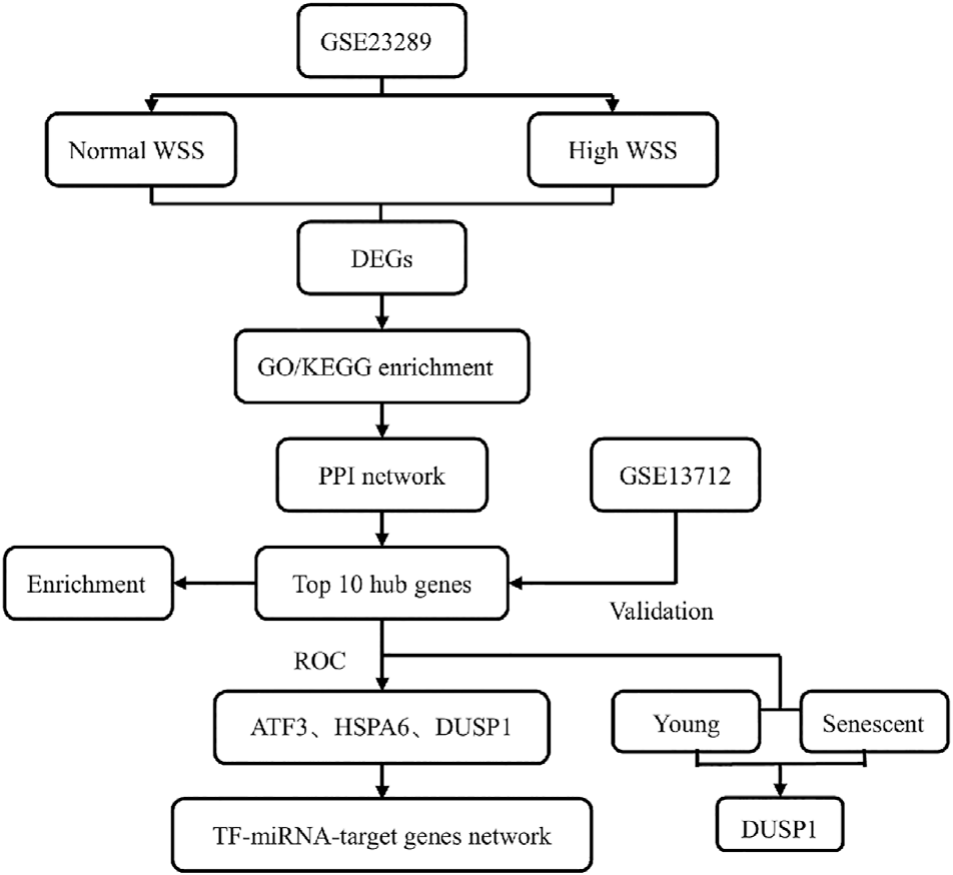
Flow chart of this study design.

In this study, we aimed to perform the differentially expressed profiles associated with the mechanosensitive genes in the human umbilical vein ECs (HUVECs) exposed to normal and high shear stress, followed by gene function and pathway enrichment analyses. After constructing a protein–protein interaction (PPI) network, we identified hub genes related to high WSS in this network and further verified them using another Gene Expression Omnibus (GEO) data set. Then, a TF–miRNA coregulatory network with validated hub genes was established (Figure 1). Finally, this study explored the valuable information for hemodynamics related to hub genes and presented a corresponding discussion for hub genes as a new potentially predicted and protected target for patients with ischemic stroke.

## Materials and Methods

### Microarray Data Sets and Screening of Differentially Expressed Genes

The GEO database is a gene expression database, mainly containing a variety of microarray data and some sequencing data, created and maintained by The National Center for Biotechnology Information (Zhao et al., 2020). The GEO database was used to obtain microarray data of ECs cultured under different WSS for the analysis of ECs-expressed specific mechanosensitive genes involved in generating mechanical force on the vascular wall in our study. The filtering criteria are as follows: first, these data sets we selected for analysis were the gene expression data of HUVECs cultured under normal and relatively high WSS; second, each data set did possess both the above two samples; third, the type of data sets was RNA expression profile by arrays. Finally, two data sets, GSE23289 and GSE13712, were selected as the test and validation sets, respectively. The microarray expression profiling data set GSE23289, generated by White SJ et al., was download from the GEO data sets (https://www.ncbi.nlm.nih.gov/geo/), which was produced on the Illumina GPL6104 platform (Illumina HumanRef-8 v2.0 Expression BeadChips). A total of eight samples were contained in this experiment, consisting of two groups (four subjects per group) with the HUVECs cultured for 24 h on gelatin-coated slides under normal and high WSS (15 dynes/cm^2^ and 75 dynes/cm^2^, respectively) on the ECs. The other microarray data set GSE13712 was annotated in the GPL570 platform [(HG-U133_Plus_2) Affymetrix Human Genome U133 Plus 2.0 Array], which included 12 samples; a comparison of the young and senescent HUVECs under normal and high WSS is given in Table 1.

**TABLE 1:** Information for selected GEO microarray data sets.

GSE accession	Platform	Samples			Sources tissue	Country	Contributor	Attribute
		Total	Normal WSS	High WSS				
GSE23289	GPL6104	8	4	4	HUVECs	United Kingdom	White SJ et al.	Test set
GSE13712	GPL570	12	6	6 (three young and three senescent)	HUVECs	South Korea	Yong Chool Boo et al.	Validation set

HSS, high shear stress; GPL6104, Illumina HumanRef-8 v2.0 Expression BeadChips; GPL570, [HG-U133_Plus_2] Affymetrix Human Genome U133 Plus 2.0 Array.

### Differential Expression Analysis

**FIGURE 2:**
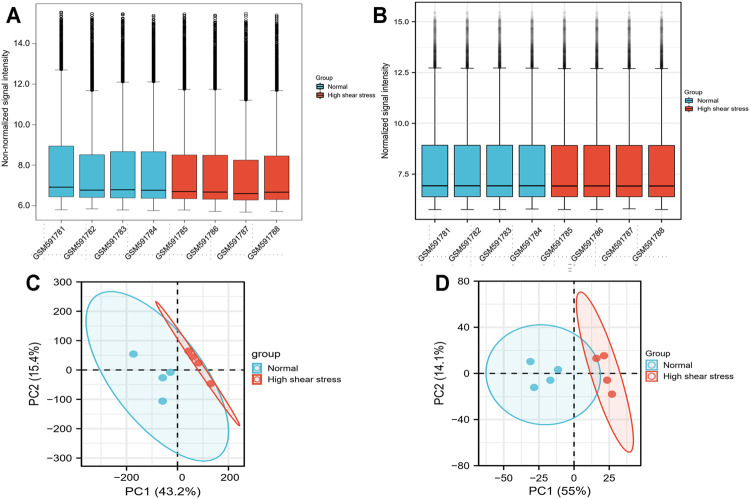
Data processing. **(A)** Boxplot for GSE23289 before quantile normalization. **(B)** Boxplot for GSE23289 after quantile normalization. **(C)** The PCA for GSE23289 before quantile normalization. **(D)** The PCA for GSE23289 after quantile normalization.

We downloaded original files of the microarray data sets, GSE23289 and GSE13712, from the GEO database with the GEOquery R package (Davis and Meltzer, 2007). The probes matching multiple genes were removed, as where the same genes corresponding to multiple probes, and the maximum median intensity probe value was taken. After filtering, the data were shown by boxplots to reflect quantile normalization, and principal component analysis (PCA) was used to demonstrate clustering of samples in different groups (Figures 2A–D). The R package “limma” (bioconductor) was performed to analyze the differentially expressed genes (DEGs) between the normal and high WSS groups (Smyth et al., 2005). The DEGs were screening out according to the following two cutoff criteria: adjusted *p*-value <0.05 and absolute log2 [fold change (FC)] >1 (Rougeot et al., 2019; Gao et al., 2020). We removed the target DEGs that had no annotated gene names. These significant DEGs were visualized in volcano plots and heat maps, which were generated in R v4.0.1 with the ggplot2 package (Gu et al., 2016).

### Functional Enrichment Analysis of Differentially Expressed Genes

To get a better understanding of the biological functions of genes, we used the R package clusterProfiler and GOplot to perform gene ontology (GO) functional enrichment and Kyoto Encyclopedia of Genes and Genomes (KEGG) analysis of the DEGs (Yu et al., 2012). GO annotations were gained from the Bioconductor R package org.Hs.eg.db for humans. The GO database consists of a set of terms that describe the properties of genes and gene products. These terms were divided into three main categories: biological process (BP), cellular component, and molecular function (MF). KEGG pathway analysis was used to assign DEGs to specific pathways for constructing networks of molecular interactions, reactions, and their relationships. The DEGs' functional enrichment and their pathway analyses were visualized by the R package ggplot2. The adjusted *p*-value <0.1 and q-value <0.2 (Benjamini–Hochberg method) was set as a default cutoff threshold.

### Protein–Protein Interaction Network Analysis, mCODE Subcluster Modules Construction, and Hub Genes Identification

A PPI network consisting of the significant DEGs were constructed by the online tool (https://string-db.org/)—with an interaction score of >0.4 (default parameters) (Hoyer et al., 2019; Li et al., 2020a; Gao et al., 2020)—the Search Tool for the Retrieval of Interacting Genes (STRING), which is a database to predict the predicted PPIs (Szklarczyk et al., 2018). The Cytoscape software (V3.8.2) was used to create and visualize the PPI network (Shannon et al., 2003). In this PPI network, each node stands for a target gene, and the lines between the nodes represents the associated interactions. The major gene clusters in the PPI network were identified by the plug-in Minimal Common Oncology Data Elements (mCODE) cluster in Cytoscape with the settings of degree cutoff = 2, node score cutoff = 0.2, k-core = 4, and max. depth = 100. The interface cytoHubba was used to explore important nodes in the biological networks. It provided many topological analysis methods; the Maximal Clique Centrality (MCC) algorithm was considered to be the most effective method to find the hub nodes. It had a better performance on the precision of predicting essential proteins from complex interaction networks (Chin et al., 2014; Li et al., 2020b). In our study, we detected the hub genes in this PPI network according to the threshold criteria of MCC calculated by a plug-in of cytoHubba in Cytoscape software.

### GO/KEGG Enrichment, Expression, and Diagnostic Analysis of Hub Genes

The GO and KEGG pathway enrichment analyses were performed for hub genes using the R package Clusterprofiler and GOplot. We defined the adjusted *p*-value of <0.05 and q-value <0.2 (Benjamini–Hochberg method) as the cutoff criteria. The bubble graph for gene functions and the network diagram representing the gene interaction pathways were drawn by the R package ggplot2. Furthermore, the sensitivity and specificity of these hub genes in predictions for EC suffering high shear stress were evaluated by the receiver operator characteristic (ROC) curves and the area under the ROC curve. The pROC R package was used for ROC curve analysis, while the ggplot2 R package was used for data visualization.

### Verification of the Specifically Expressed Hub Genes

Additional publicly available data set GSE13712 was used to further validate the specific expression patterns of such hub genes. Then two-sample t-test was used to compare the differential expression values of the hub genes between ECs lying in the normal and high WSS groups. Finally, a post-analysis was conducted between the young and old EC subgroups under high shear stress to identify the expression of hub genes. The hub gene expression levels between the two subgroups were performed using a t-test, and *p*-values <0.05 were regarded as statistically significant. The violin plots comparing the expression levels of the selected hub genes in the different groups were depicted by the R package ggplot2. Statistical significance was indicated as follows: *(*p* < 0.05), **(*p* < 0.01), and ns (*p* > 0.05, no significance).

### TF–miRNA Coregulatory Network

We obtained TF–miRNA co-regulated interaction data from the RegNetwork repository, which contributes to predicting miRNAs and TFs that regulate the hub genes of interest at the transcriptional and post-transcriptional levels (Liu et al., 2015). The NetworkAnalyst platform (https://www.networkanalyst.ca/) was used to identify and visualize the TF genes–miRNAs interactions with selected hub genes using a cutoff degree >1.

## Results

### Identification of Differentially Expressed Genes

**FIGURE 3:**
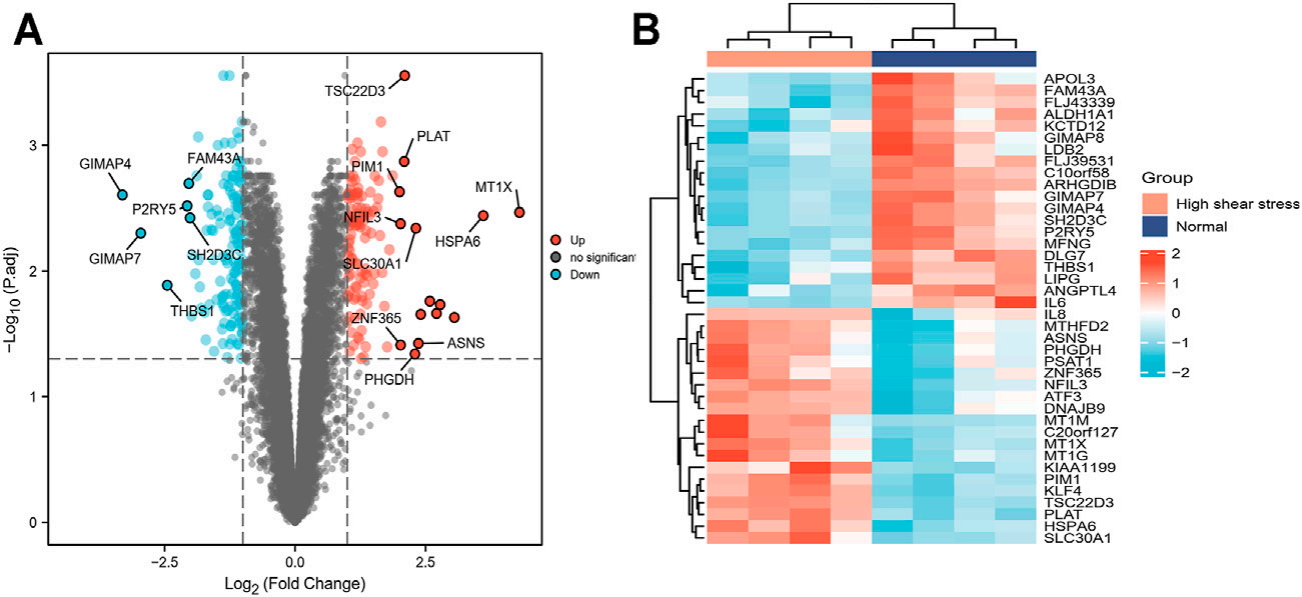
Screening of DEGs between normal and high WSS groups in GSE23289. **(A)** The volcano plot of DEGs, in which the red dots represent upregulated genes, blue dots represent downregulated genes, and grey dots represent genes with no significant difference. The y-axis indicates the −log10 (adjusted *p*-value), and the x-axis indicates the log2FC. FC is the fold change. **(B)** The cluster heat map of DEGs with each row corresponding to a gene and each column corresponding to a sample. The color scale ranges from light blue, showing relatively low gene expression levels, and passes through white to red, representing relatively high gene expression levels.

The data set GSE23289, which included eight samples divided into two groups (HUVECs were cultured under the normal and the high WSS, respectively), identified and analyzed the DEGs using the R package (limma). As a result, using the cutoff criteria (adjusted *p*-value <0.05 and |log2FC|>1), compared with the normal WSS groups, a total of 260 DEGs (121 upregulated and 139 downregulated genes) were identified in the high WSS groups, which provided the underlying clues to study atherogenesis and atherosclerotic progression (Supplementary Table 1). These significantly DEGs in subsequence analysis were visualized via volcano plot and heat map. These DEGs with distinct changes (adjusted *p*-value <0.05 and |log2FC| >2) were marked by their symbols on the volcano plot diagram. Among all DEGs, the top 20 highly expressed genes and the top 20 lowly expressed genes, respectively, were selected to visualize in the heat map (Figures 3A,B).

### GO and KEGG Functional Analysis for Differentially Expressed Genes

**FIGURE 4:**
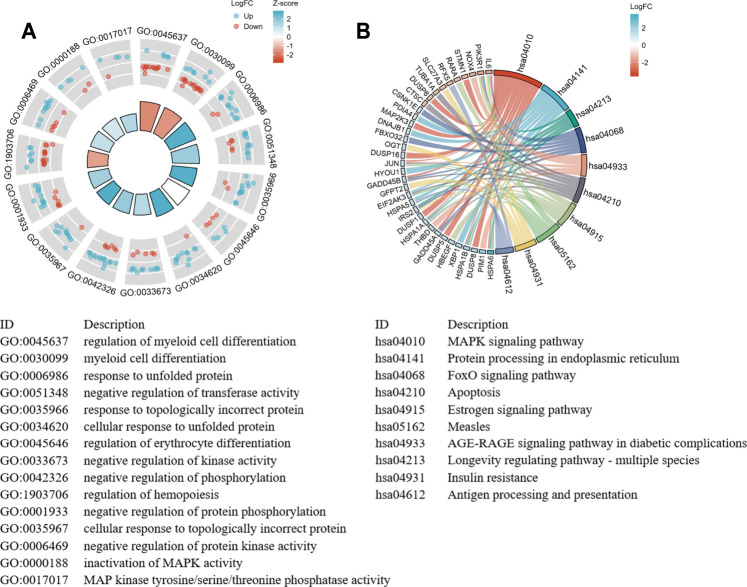
The significant enrichment GO terms and KEGG pathways of the DEGs. **(A)** GO functional enrichment Circos plots of DEGs. Each column of the inner circle corresponds to a GO term. The height represents the adjusted *p*-value. The higher the column, the smaller the adjusted *p*-value. The filled color of the column represents the z-score value corresponding to the GO terms. **(B)** Signaling pathways enrichment chord diagram of DEGs. The left half represents the DEGs, the right half shows the pathways, and the size of the color block represents the corresponding counts. The chord lines between the left and right parts represent links between DEGs and specialized signaling pathways.

The GO and KEGG functional pathways enrichment analyses of 260 DEGs were performed at the threshold of adjusted *p*-value <0.1 and q-value <0.2 using the R package clusterProfiler. Overall, we found that 241 GO-BPs, 16 GO-MFs, and 16 KEGG pathways were highly enriched for these DEGs (Supplementary Table 2). The results of the GO terms showed that regulation of myeloid cell differentiation (GO:0045637), myeloid cell differentiation (GO:0030099), and response to unfolded protein (GO:0006986) were significantly enriched processes in the BP category. The apparently enriched entries for the MF category were mitogen-activated protein kinase (MAPK) tyrosine/serine/threonine phosphatase activity (GO:0017017), MAPK phosphatase activity (GO:0033549), and misfolded protein binding (GO:0051787). Additionally, the KEGG pathways enrichment analyses indicated that DEGs were enriched in the MAPK signaling pathway (hsa04010), protein processing in the endoplasmic reticulum (hsa04141), and longevity regulating pathway–multiple species (hsa04213). Finally, we displayed the top 15 GO terms and top 10 KEGG pathways enriched results (Figures 4A,B).

### PPI Network Analysis, mCODE Cluster Modules, and Hub Gene Identification

**FIGURE 5:**
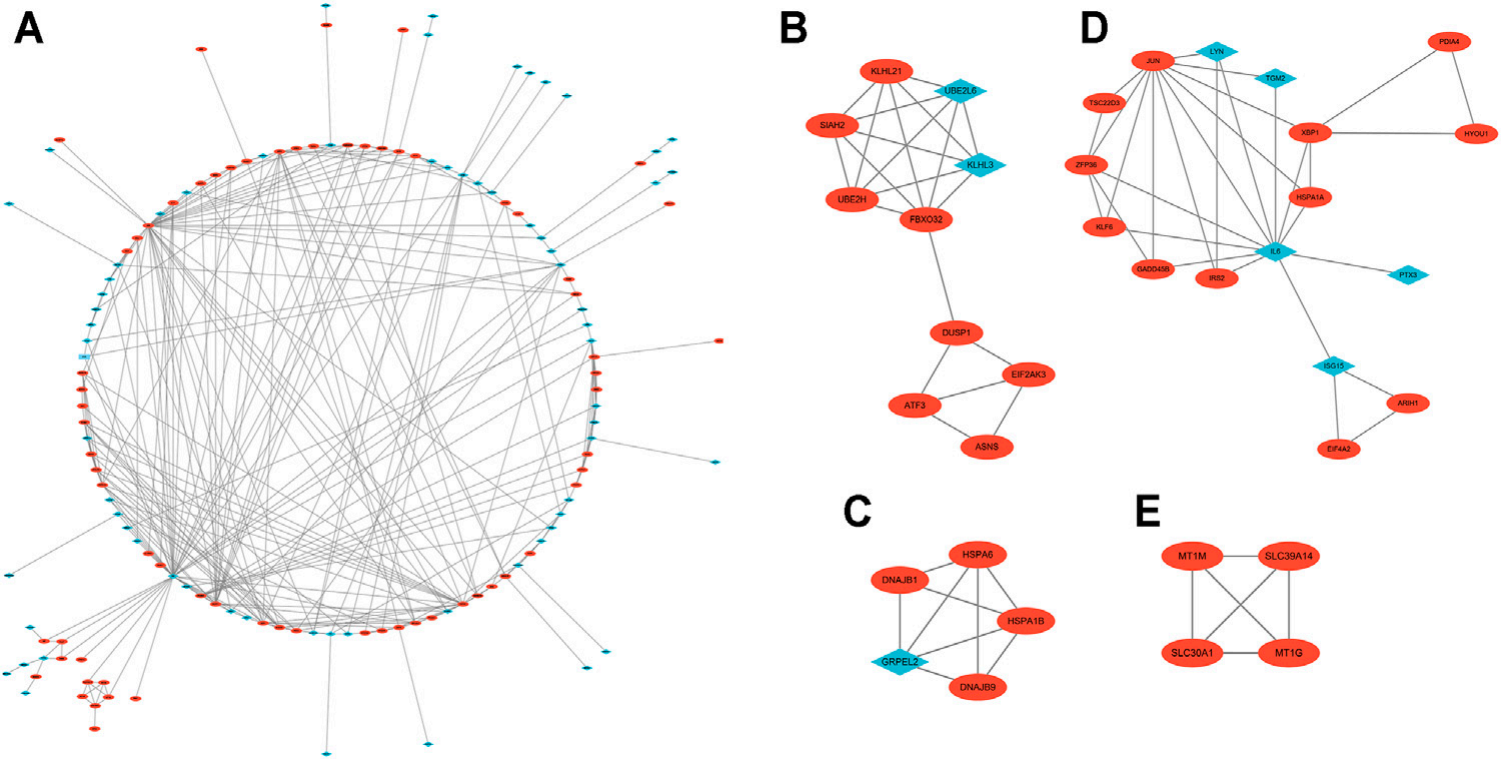
The PPI network and two cluster modules were extracted by mCODE. **(A)** The PPI network of DEGs in GSE23289, which comprised 167 nodes and 334 edges. Each node represents a protein, while each edge between the two proteins shows a protein–protein correlation. The up- or downregulated genes are highlighted as red circles and blue diamonds, respectively. **(B)** Cluster mCODE 1 consists of 10 nodes and 21 edges. mCODE score = 4.667. KLHL21 is the seed gene in this subnetwork. **(C)** Cluster mCODE 2 includes five nodes and nine edges. mCODE score = 4.5. DNAJB9 is the seed gene in this subnetwork. **(D)** Cluster mCODE 3 includes four nodes and six edges, MT1M is the seed gene, score = 4. **(E)** Cluster mCODE 4 includes 17 nodes and 31 edges, ZFP36 is the seed gene, and score = 3.882).

**FIGURE 6:**
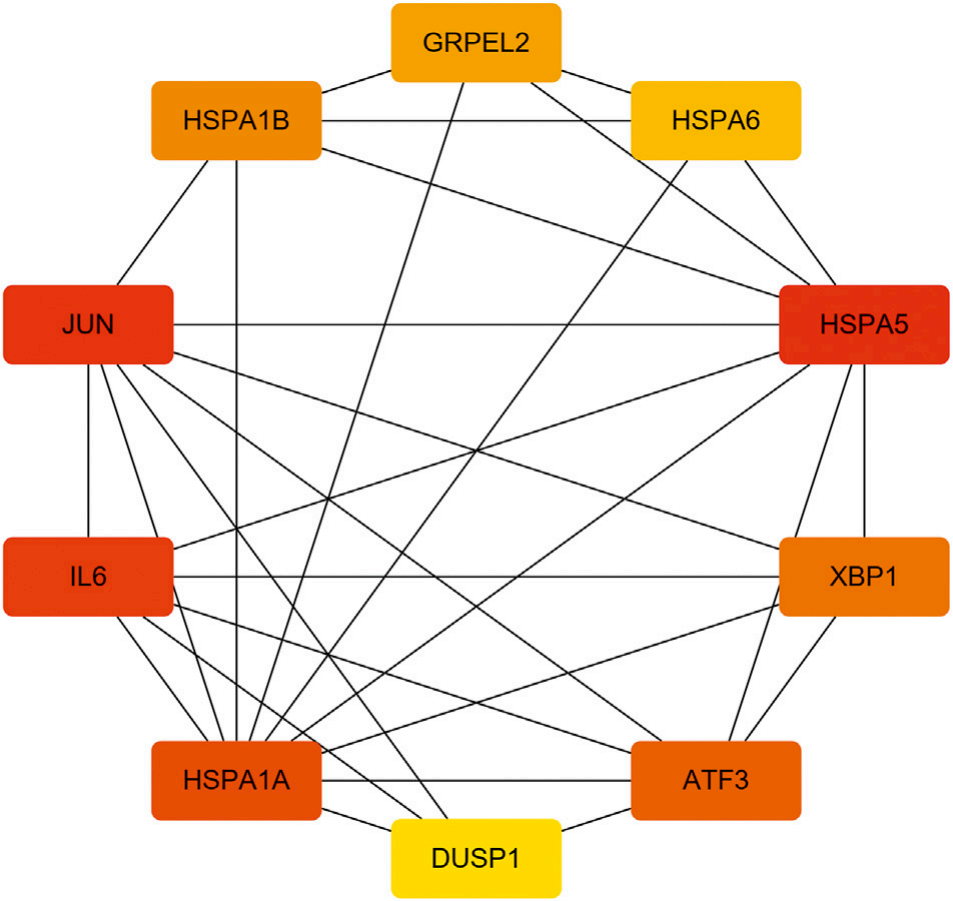
The 10 hub genes interaction network. Each node indicates a gene, and each edge between the two nodes indicates a possible interrelationship of those two genes. The color gradient of the node reflects the MCC score, and the darker the color, the higher the score.

**FIGURE 7:**
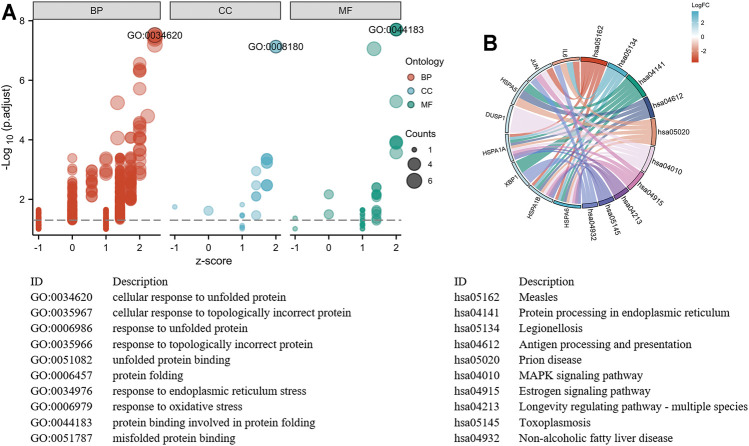
Top 10 hub genes functional enrichment analysis. **(A)** The GO functional enrichment bubble plot of hub genes. The abscissa represents the z-score, and the ordinate represents the adjusted *p*-value. The size of the bubble displayed is corresponding to the counts enriched in the GO terms, and the bubble's color correlated with the enriched GO categories. **(B)** The signaling pathways enrichment chord diagram of hub genes.

With the STRING online tool and the Cytoscape software, we established a PPI network related to the above 260 DEGs. The PPI network included 167 nodes and 334 edges, as shown in Figure 5A. Using the mCODE plug-in, four significant cluster modules were obtained from the PPI network based on the screening criteria. Module 1 had the highest cluster score of 4.667, which included a total of 21 edges, 10 nodes, and a seed gene named KLHL21 (Figure 5B). The Module 2 subnetwork had a cluster score of 4.5, which consisted of nine edges, five nodes, and a seed gene named DNAJB9 (Figure 5C). Module 3 had a cluster score of 4, which included a total of six edges, four nodes, and a seed gene named MT1M (Figure 5D). The Module 4 subnetwork had the cluster score of 3.882, which consisted of 31 edges, 17 nodes, and a seed gene named ZFP36 (Figure 5E). Next, we filtered the top 10 hub genes ranked by the MCC algorithm [HSPA5, JUN, IL6, HSPA1A, activated transcription factor 3 (ATF3), XBP1, HSPA1B, GRPEL2, heat shock protein family A (Hsp70) member 6 (HSPA6), and dual specificity phosphatase 1 (DUSP1, also known as CL100, HVH1, MKP-1, PTPN10)], using cytoHubba plug-in (Figure 6; Table 2). These hub genes may be potentially mechanosensitive genes that have crucial roles in sensing the force stretch stimulation of ECs directly. Finally, these hub genes' functional enrichment analyses were performed by the R clusterProfiler package, and it was found that they were dramatically enriched in GO terms such as cellular response to unfolded protein, COP9 signalosome, heat shock protein binding, and KEGG signaling pathways such as protein processing in the endoplasmic reticulum and the MAPK signaling pathway. According to the criteria, all summary results of meaningful functional annotation analyses from the hub genes were shown in Figure 7; Supplementary Table 3.

**TABLE 2:** Top 10 hub genes ranked by MCC method in cytoHubba.

Gene symbol	Description	log2FC	adjusted *p*-value	Score	Regulation
HSPA5	Heat shock protein family A (Hsp70) member 5	1.273662	0.029748	641	Up
JUN	Jun proto-oncogene, AP-1 transcription factor subunit	1.193381	0.003161	572	Up
IL6	Interleukin 6	−1.69372	0.004736	501	Down
HSPA1A	Heat shock protein family A (Hsp70) member 1A	1.354534	0.040084	438	Up
ATF3	Activating transcription factor 3	2.710495	0.021757	410	Up
XBP1	X-box binding protein 1	1.498306	0.012617	347	Up
HSPA1B	Heat shock protein family A (Hsp70) member 1B	1.669	0.010268	264	Up
GRPEL2	GrpE like 2, mitochondrial	−1.07237	0.023067	252	Down
HSPA6	Heat shock protein family A (Hsp70) member 6	3.606503	0.003621	240	Up
DUSP1	Dual specificity phosphatase 1	1.315756	0.048207	201	Up

### The Expression Levels of Hub Genes in the Validation Data Set

**FIGURE 8:**
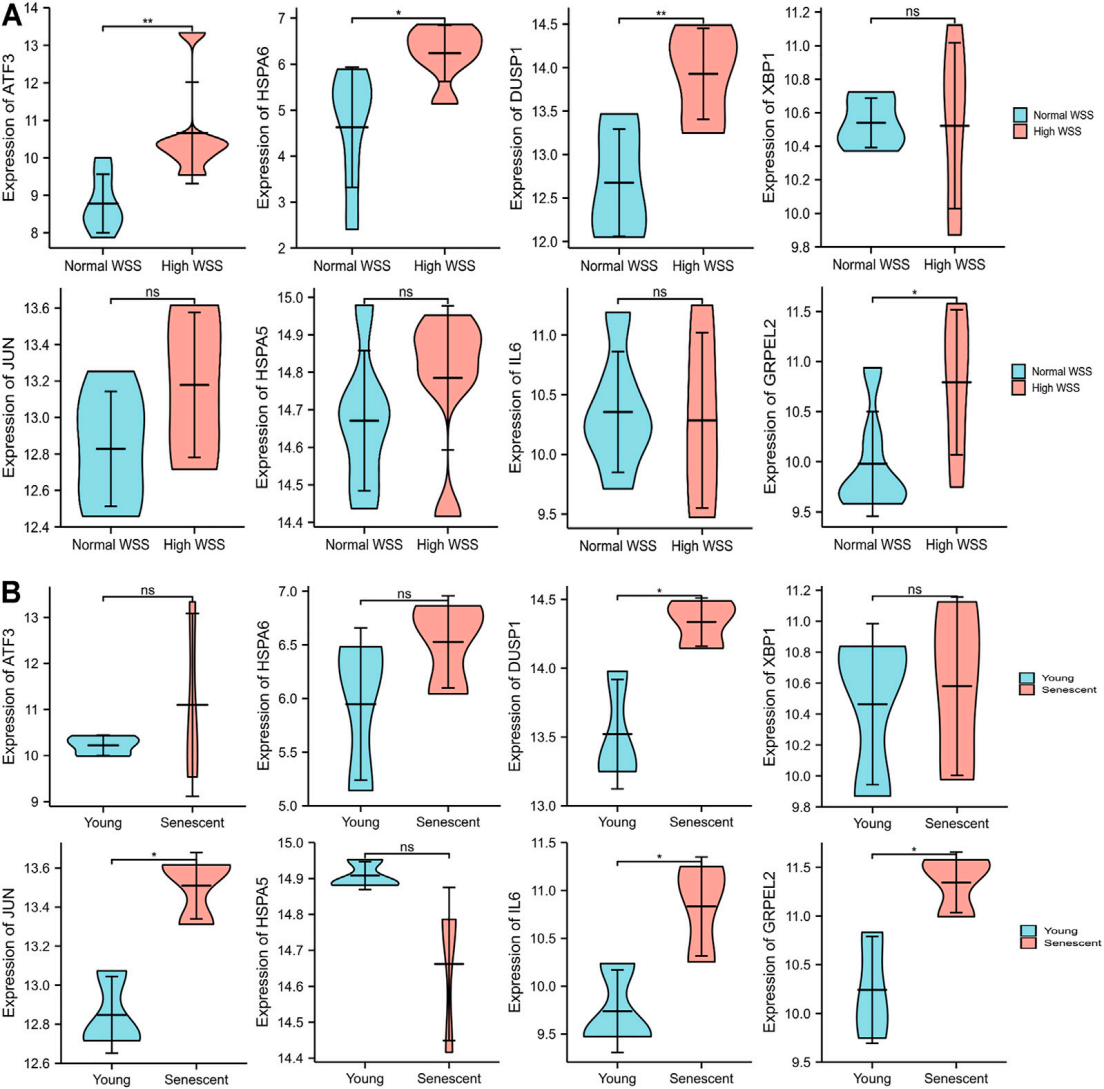
The expression level validation of hub genes. **(A)** A comparison of the expressed level of the hub genes between normal and high WSS groups in the GSE13712 data set. Compared with the normal group, hub genes (ATF3, DUSP1, HSPA6, and GRPLE2) are highly expressed in the high WSS group with significance. **(B)** The comparison of the expressed levels of the hub genes between the young and senescent HUVEC groups under high WSS in the GSE13712 data set. Compared with the young HUVECs group, DUSP1, GRPEL2, and JUN were highly expressed in the senescent HUVECs group, while others have no significance. *p < 0.05, **p < 0.01, ns: *p* > 0.05, no significance.

Due to the removal of probes matching multiple genes, only eight genes were retained, and the HSPA1A/HSPA1B genes were excluded in GSE13712. The expression profiles of these eight hub genes were validated by the GSE13712 data set. The ggplot2 package of R software was performed to draw violin plots and conducted a t-test for the comparison of the two groups. Compared with the normal group, the expression levels of the hub genes, ATF3 and DUSP1, in the relatively high WSS group were significantly increased (*p* < 0.01). In addition, the expression levels of the another two hub genes, HSPA6 and GRPEL2, were also strongly upregulated in high WSS (*p* < 0.05) (Figure 8A). To investigate whether aging can affect mechanosensitive genes, the expression of the abovementioned hub genes was statistically analyzed according to the different degrees of aging HUVECs. We observed that the expression levels of DUSP1, GRPEL2, and JUN genes in the senescent HUVECs group were significantly higher than those in the young HUVECs group under high WSS (*p* < 0.05) (Figure 8B). However, the expression level of GRPLE2 in the GSE13712 was inconsistent with that in the GSE23289 data set. In the GSE23289 data set, GRPLE2 was downregulated in the high WSS group, while in the GSE13712 data set, the gene was upregulated. The possible reasons for the inconsistency of results among the two data sets could be the different chip platforms for analyzing the data and the small sample size of these GEO data sets. Ultimately, we found that DUSP1 may be an important aging-related mechanosensitive gene because it is not only significantly upregulated in the high WSS group but also more significantly upregulated in the senescent HUVECs than in the young HUVECs.

### The Predictive Performance Based on ROC Curve of Hub Genes

**FIGURE 9:**
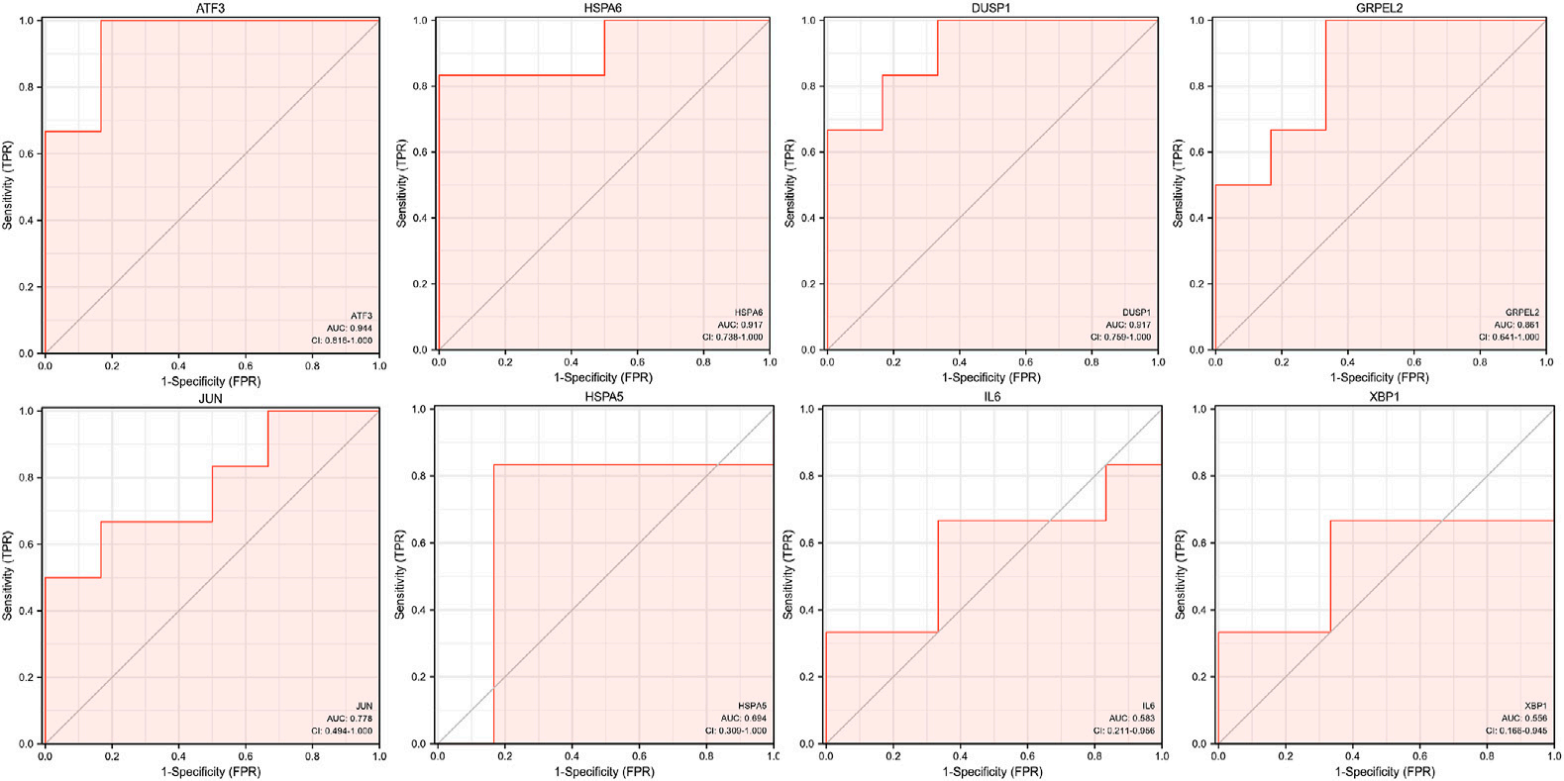
The ROC curves of eight hub genes.

We used the pROC package in R software to analyze the expression profiles of the above specifically expressed hub genes in the normal group, high shear stress group, and young HUVECs and senescent HUVECs groups under high shear stress and drew the ROC curves by ggplot2. The area under the curve (AUC) is an indicator that combines sensitivity and specificity, which can describe the inherent validity of a diagnostic test. Compared with the normal group, these five specifically expressed hub genes (ATF3, HSPA6, DUSP1, GRPLE2, and JUN) have higher diagnostic value in the high shear stress group (0.7<AUC<1.0). Among them, ATF3 has the strongest diagnostic ability with an AUC of 0.944, followed by HSPA6 and DUSP1, which have the same AUC value of 0.917, and the AUC of GRPLE2 and JUN were 0.861 and 0.778, respectively. While in the senescent HUVECs group under high WSS, DUSP1 was found to have the highest diagnostic value (AUC = 1.0), which was consistent with the previous hub genes expression verification results between the young and senescent HUVEC groups (Figure 9). Therefore, after combining the above analysis of the eight hub genes expression levels with the ROC results, we concluded that ATF3, HSPA6, and DUSP1 might be the most potential mechanosensitive genes, being upregulated in high shear stress with statistical significance, which could have an important role in sensing the mechanical tension stimulus of the blood vessel wall directly. Moreover, under high shear stress, DUSP1 was expressed at a higher level in senescent HUVECs than in young HUVECs. Therefore, we speculated that DUSP1 might be a mechanosensitive gene related to the degree of aging based on our current samples data.

### TF–miRNA Coregulatory Network

**FIGURE 10:**
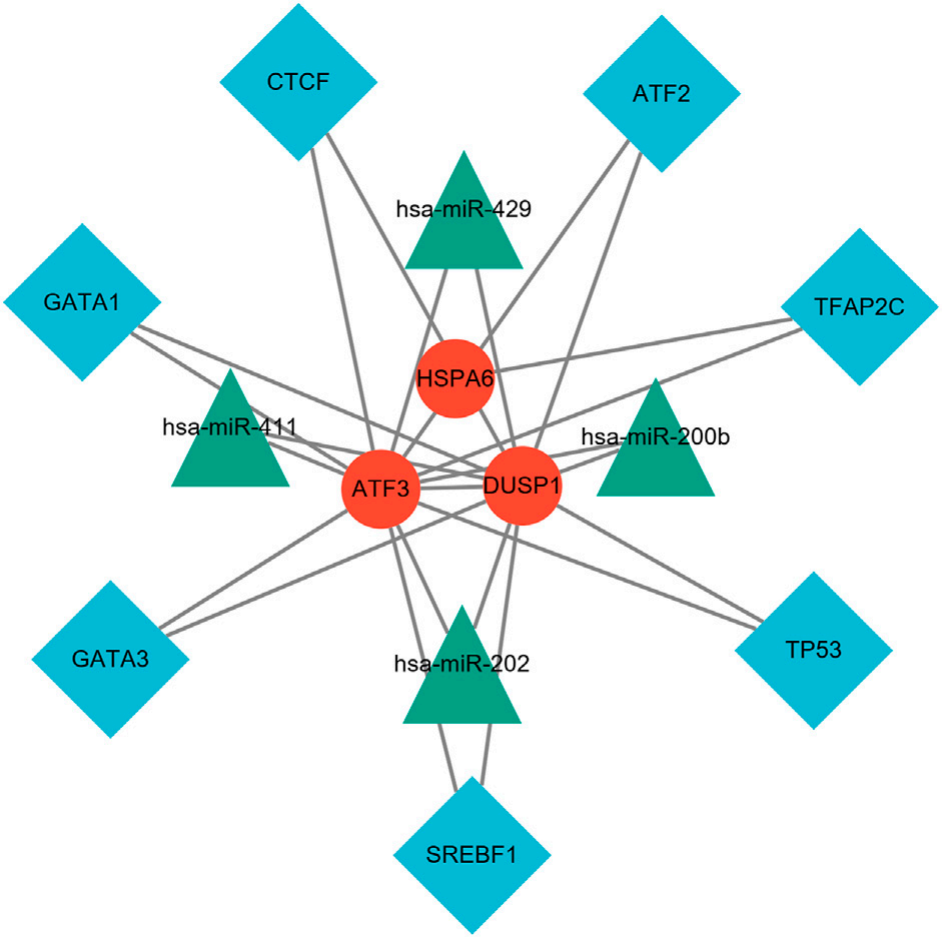
TF–miRNA coregulatory network. The network consisted of 14 nodes and 23 edges, including seven TF genes and four miRNAs, and three validated hub genes. The red round nodes are validated hub genes, the blue diamond nodes are TF genes, and the green triangle nodes are miRNA genes.

NetworkAnalyst online platform is used to generate TF–miRNA co-regulatory network. The interaction between TFs and miRNAs and the selected hub genes was delivered via the TF–miRNA co-regulatory network analysis. This interaction may be the underlying mechanism for the regulation of hub genes expression. The TF–miRNA co-regulatory network consisted of 14 nodes and 23 edges. A total of four miRNAs and seven TF genes interacted with the validated hub genes. Figure 10 shows the TF–miRNA co-regulatory network.

## Discussion

Stroke is the main cause of long-term disability and the second most common cause of death in the world with an increasing burden on global health (sD and Collaborators, 2016; MortalityD (2016). Cau, 2016; Feigin et al., 2017). Ischemic stroke, which accounts for nearly 80% of all types of stroke, is mainly caused by an *in situ* blockage of a blood flow, resulting from a narrowing large artery in the brain circulation, or emboli from a ruptured atherosclerotic plaque blocking downstream branches (Hademenos and Massoud, 1997). Previous studies have shown that there is a significant correlation between WSS and stroke events, and WSS is considered to be the main risk factor for atherosclerotic disease and ischemic stroke (Cicha et al., 2011; Leng et al., 2019). WSS is the frictional resistance exerted by the blood flow that acts on and parallel to the surface of the endothelium. Low WSS may promote the deposition of particles on the arterial wall and the accumulation of plaque, while high WSS not only stimulates adaptive outward remodeling but also destabilizes atherosclerotic plaques in narrowed blood vessels by directly acting on plaques, inducing inflammation, enhancing oxidative stress, and subsequently causing embolization of distal arteries (Dolan et al., 2013; Chen et al., 2019; Goudot et al., 2020). The ability of blood vessels to react to changes in hemodynamic stimuli is mediated primarily by ECs, which form the internal layer of blood vessel walls. Since WSS directly acts on vascular ECs, it is speculated that mechanical tension stimulates mechanics-sensitive genes on the endothelium, induces EC injury and dysfunction, and triggers a host of biological responses noted above. Another study illustrates that correcting hemodynamic factors plays a protective role in the blood vessels (Dhawan et al., 2010). Therefore, it is essential to study the mechanism of WSS-induced ischemic events and to identify new potential molecular targets for disease diagnosis and treatment. Bioinformatics technology has been widely used to discuss the specificity of gene expression during the occurrence and development of diseases. Moreover, it is a reliable way to explore validated diagnosis and treatment targets of disease.

At present, there is little research on the correlation between WSS and mechanics-sensitive genes expressed on vascular ECs and its mechanism of cerebrovascular health promotion and stroke prevention. In our study, using bioinformatics methods, we analyzed the expression profiles of different genes on vascular ECs under high and normal WSS, and a total of 260 DEGs were observed with 121 upregulated and 139 downregulated genes. Many, mainly the biological functions, of these DEGs were screened out, including cellular response to unfolded protein and topologically incorrect protein, response to endoplasmic reticulum stress, oxidative stress, heat shock protein binding, etc. The KEGG signaling pathways that we enriched included protein processing in the endoplasmic reticulum, antigen processing and presentation, the MAPK signaling pathway, and the signaling pathways associated with infection and inflammatory disease. After the 10 hub genes filtered by the PPI network were validated using another GEO data set, we determined eight hub genes (HSPA5, JUN, IL6, ATF3, XBP1, GRPEL2, HSPA6, and DUSP1). The ROC curve analysis shows that ATF3, HSPA6, DUSP1, GRPLE2, and JUN have significant diagnostic values in the high WSS group (0.7 < AUC < 1.0). Combined with these eight hub genes' expressed levels in high and normal WSS, ATF3, HSPA6, and DUSP1 were significantly different and upregulated in high WSS (*p* < 0.05). Subsequent analysis of the above genes in the subgroups revealed that in the high WSS group, compared with young cells, the expression level of DUSP1 was statistically significant in the senescent cells (AUC = 1, *p* = 0.032). Therefore, we hypothesized that ATF3, HSPA6, and DUSP1 may be mechanics-sensitive genes expressed on vascular ECs under high WSS based on our current samples. And among them, DUSP1 may be positively correlated with the degree of EC senescence. In addition, we constructed a TF–miRNA co-regulatory network, which provides the interaction between miRNA and TF with ATF3, HSPA6, and DUSP1. This interaction may be the reason for the regulation of mechanics-sensitive genes expression.

ATF3, an immediate, early transcription factor gene belonging to the activated protein 1 (AP-1) family, plays an important role in inflammation and infectious diseases, endoplasmic reticulum stress, mitochondrial oxidative stress, endothelial dysfunction, and prevention of atherosclerosis (Cai et al., 2000; Nawa et al., 2002; Aung et al., 2013; Rohini et al., 2018). Teasdale et al. (2017) found that elevated laminar flow in blood vessels can induce the expression of ATF3 and reduce the expression of inflammatory genes independent of nuclear factor-κB activation. They also identify that ATF3 is an important protective factor against vascular endothelial dysfunction, and regulating the expression of ATF3 may be a new way to modulate the expression of pro-inflammatory genes. It is suggested that ATF3 may play a pivotal role in the mechanism of vascular endothelial dysfunction and atherosclerotic plaque rupture induced by high WSS. Consistent with our study, we found that ATF3 was upregulated in HUVECs cultured under high WSS. In addition, the ROC curve showed that ATF3 has a high diagnostic value for high WSS (AUC = 0.944). Therefore, we considered ATF3 as a gene that is very sensitive to high WSS.

HSPA6 (also called Hsp70B'), is an inducible member of the HSPA (Hsp70) multigene family. Compared with the extensively studied HSP70 family genes, it has received little attention because it is the only gene expressed in the human genome but not in rodents such as mice and rats (Noonan et al., 2007; Deane and Brown, 2017). Previous studies have shown that HSP70 can be expressed in atherosclerotic plaques, and the high expression of HSP70 is positively correlated with the severity of atherosclerotic vascular lesions (Konstantinova et al., 2019). In addition, HSP70 has been shown to have crucial functions in inflammation/immune-related regulation and vascular endothelial dysfunction and to be linked to a series of inflammatory diseases such as atherosclerosis, arthritis, neurodegenerative diseases, as well as cancer (Thériault et al., 2005; Henderson and Pockley, 2010; Costa-Beber et al., 2020). A few studies referred to HSPA6. McCullagh et al. (2016) found that overexpression of HSPA6 can inhibit the proliferation of smooth muscle cells, which suggested that HSPA6 may play a crucial role in targeting therapy for vascular repair. Further studies have shown that HSPA6 is preferentially expressed in macrophages and is considered to have pro-inflammatory properties. It synergizes with circulating immune complexes to promote the progression of atherosclerotic processes, which could promise a new therapeutic target for regulating vulnerable atherosclerotic plaques (Hammad et al., 2009; Smith et al., 2010). At present, there is no research on the relationship between HSAP6 and WSS. However, in our study, HSP70 family genes, including HSPA1A, HSPA1B, HSPA5, and HSPA6, were significantly upregulated under high WSS in vascular ECs. Among them, HSPA6 served as the hub gene. It demonstrated that HSP70, especially the less studied HSPA6, maybe a mechanosensitive gene, which plays a key role in the progression of atherosclerotic disease and the ruptured atherosclerotic plaques.

DUSP1 belongs to a family of inducible nuclear dual-specific phosphatases that can dephosphorylate tyrosine and threonine/serine residues. Integrated analyses of ischemic stroke data sets from many previous studies have shown that DUSP1 is overexpressed in both males and females, which suggested that DUSP1 may be a diagnostic biomarker for ischemic stroke (Li et al., 2016; Zhang et al., 2019; Feng et al., 2021). Currently, the mechanism by which DUSP1 regulates atherosclerosis progression is still controversial. ZAKKAR M and HOPPSTADTER J et al. thought that the increased expression of DUSP1 is anti-atherogenic *via* inhibiting EC pro-inflammatory signaling pathways, such as MAPK and NF-κB (Zakkar et al., 2016; Hoppstädter and Ammit, 2019). Shen et al. (2010) considered that the deletion of DUSP1 leads to a reduction of atherosclerotic lesion formation, which is accompanied by decreases of multiple pro-inflammatory cytokines, including TNF and IL-1. Similar views have shown that DUSP1 is overexpressed in atherosclerotic lesion, correlated with hyperlipidemia, and reduced DUSP1 can inhibit the development of atherosclerosis (Reddy et al., 2004). In addition, a study by Lee et al. (2016) indicated that DUSP1 involves in the regulatory mechanism of cellular senescence *via* immunomodulatory properties. In our present study, unlike the abovementioned ATF3 and HSPA6, we identified that DUSP1 was not only highly expressed on vascular ECs under high WSS but also highly expressed in the senescent cells subgroup than in young cells (*p* = 0.0315 AUC = 1.000), indicating that DUSP1 may be a mechanosensitive gene related to high WSS and correlated with the senescence of vascular ECs.

Furthermore, the target TF genes and miRNAs were predicted for ATF3, HSPA6, and DUSP1, and a TF–miRNA–mechanosensitive genes coregulatory network was constructed by the NetworkAnalyst. This co-regulatory network revealed the regulatory mechanism of mechanosensitive genes at the transcriptome level. Among the most interactive TFs, the higher degree of TFAP2C was 2, and the higher betweenness was 1692. TFAP2C and ATF3 play multiple roles in shaping the sensitivity of ferroptosis through either transcription-independent or transcription-dependent mechanisms (Dai et al., 2020). Pharmacological components based on TFAP2C can be constructed to inhibit ferroptosis and cell death caused by excitotoxicity or endoplasmic reticulum stress and coordinate the repair of neurons after stroke (Alim et al., 2019). Evidence for changes in miRNA expression in the co-regulatory network has been established in various studies. For instance, has-miR-429 regulates the inflammatory response by targeting inhibition of DUSP1 expression (Qi and Wang, 2020). van der Kwast et al. (2020) demonstrated that hsa-miRNA-411, regarded as vasoactive miRNA, was abundantly expressed in the vasculature for chronic ischemic diseases. The TF genes are a reactor that regulates gene expression and complete regulation by combining with target genes or miRNA. On the other hand, miRNAs induce gene silencing and downregulate gene expression by binding to target genes (Zhang et al., 2015b).

Several limitations of this study should be considered. First, our study did not truly combine transcriptomic microarray data, since we use only one data set to discover mechanosensitive genes and another one as a validation data set. The sample size was not large enough, limited by the publicly available microarray data sets, which require further samples to be sent of our own for transcriptomic high-throughput sequencing or microarray data to expand the sample size. Second, recently, we did not illustrate the real co-relations between high shear stress, processes of atherogenesis, and atherosclerotic progression due to the lack of animal models in a different stage of atherosclerosis. Additional in-depth and more rigorous experimental studies will be designed to focus on the role of high shear stress in the processes of atherogenesis and atherosclerotic progression. Third, the work was solely based on bioinformatic strategies. Although we validated the potential three mechanosensitive genes in a public microarray data set, an in-depth validation of the hub genes would greatly enhance the amount of information in this article. Specifically, a mechanistic study based on the proposed TF–miRNA genes coregulatory network could better demonstrate the atherosclerotic disease progressions. But unfortunately, the experiment was halted due to the recurrent episodes of severe acute respiratory coronavirus 2 (SARS-CoV-2).

## Conclusion

In this work, we identified three hub genes, namely, ATF3, HSPA6, and DUSP1, as potential mechanosensitive genes under high WSS in blood vessels. Among them, DUSP1 was confirmed to be associated with the senescence of vascular ECs that experienced high WSS. Therefore, these three mechanosensitive genes may have emerged as potential novel targets for the prediction and prevention of ischemic stroke. In addition, a TF–miRNA–mechanosensitive genes coregulatory network was constructed, which revealed an underlying regulatory mechanism and the pathways to control disease progression.

## Data Availability

The data sets presented in this study can be found in online repositories. The names of the repository/repositories and accession number(s) can be found in the article/Supplementary Material.
