# Supplementary material for: Prediction of Mechanosensitive Genes in Vascular Endothelial Cells Under High Wall Shear Stress
Source: Front Genet. 2022 Jan 11;12:796812. doi: 10.3389/fgene.2021.796812 (PMC8787366; doi:10.3389/fgene.2021.796812)
Supplement: Supplementary file 1 [file DataSheet1.docx]

Supplementary Material

# Supplementary Tables

## Supplementary Table1| 260 DEGs of GSE23289

| Gene.Symbol | adj.P.Val | P.Value | logFC |
| --- | --- | --- | --- |
| TSC22D3 | 0.000279 | 6.31E-08 | 2.101354 |
| GMFB | 0.000279 | 6.13E-08 | -1.25813 |
| ATIC | 0.000279 | 3.79E-08 | -1.37237 |
| FBXO34 | 0.000651 | 3.26E-07 | 1.645628 |
| DERA | 0.000651 | 3.80E-07 | -1.01703 |
| DSCR2 | 0.00079 | 6.07E-07 | -1.10237 |
| ARHGDIB | 0.000857 | 7.07E-07 | -1.85529 |
| FAM36A | 0.00088 | 7.73E-07 | -1.07768 |
| MYCT1 | 0.000958 | 9.53E-07 | 1.197073 |
| RFX5 | 0.000958 | 1.00E-06 | -1.21393 |
| C6orf108 | 0.000958 | 1.05E-06 | -1.34688 |
| GPX1 | 0.001 | 1.15E-06 | -1.47724 |
| PHF13 | 0.001076 | 1.30E-06 | 1.094404 |
| DUSP8 | 0.001124 | 1.48E-06 | 1.677482 |
| AYTL2 | 0.001124 | 1.45E-06 | 1.257676 |
| PLAT | 0.001345 | 2.19E-06 | 2.091558 |
| ARID5A | 0.001345 | 2.45E-06 | 1.146562 |
| NMRAL1 | 0.001345 | 2.30E-06 | -1.0339 |
| S100A13 | 0.001345 | 2.36E-06 | -1.0803 |
| ACAA2 | 0.001532 | 3.10E-06 | -1.11804 |
| GALK1 | 0.00158 | 3.30E-06 | -1.01756 |
| KLF4 | 0.00175 | 5.57E-06 | 1.869136 |
| STX1A | 0.00175 | 6.30E-06 | 1.529953 |
| PIP5K2A | 0.00175 | 6.13E-06 | 1.114494 |
| POFUT2 | 0.00175 | 7.47E-06 | 1.045673 |
| C1orf212 | 0.00175 | 4.50E-06 | -1.0005 |
| DBNL | 0.00175 | 5.81E-06 | -1.05481 |
| TUBA1A | 0.00175 | 4.24E-06 | -1.17283 |
| SLC27A3 | 0.00175 | 6.13E-06 | -1.19322 |
| ASF1A | 0.00175 | 7.28E-06 | -1.31855 |
| C10orf58 | 0.00175 | 5.30E-06 | -1.88391 |
| OGT | 0.001885 | 9.53E-06 | 1.152659 |
| ZNF364 | 0.001908 | 9.92E-06 | 1.603555 |
| C17orf58 | 0.001908 | 1.01E-05 | -1.19759 |
| RNASE4 | 0.001945 | 1.11E-05 | 1.087181 |
| LCMT2 | 0.001945 | 1.10E-05 | -1.07696 |
| PRKCDBP | 0.001995 | 1.18E-05 | -1.02786 |
| FAM43A | 0.002014 | 1.23E-05 | -2.03584 |
| EPM2AIP1 | 0.002029 | 1.25E-05 | -1.36636 |
| NME1 | 0.002105 | 1.35E-05 | -1.27651 |
| GPRC5A | 0.002201 | 1.51E-05 | 1.211126 |
| PDIA4 | 0.00226 | 1.59E-05 | 1.067283 |
| TEAD2 | 0.002312 | 1.75E-05 | -1.24069 |
| PIM1 | 0.002347 | 1.87E-05 | 2.00246 |
| ABL2 | 0.002361 | 1.90E-05 | 1.44406 |
| LMCD1 | 0.002392 | 2.04E-05 | 1.202944 |
| TMEM170 | 0.002392 | 2.05E-05 | 1.19321 |
| RSRC2 | 0.002392 | 2.10E-05 | 1.032509 |
| DUSP16 | 0.002486 | 2.44E-05 | 1.153691 |
| RBM33 | 0.002486 | 2.29E-05 | 1.083648 |
| CSNK1E | 0.002486 | 2.52E-05 | 1.01291 |
| ARIH1 | 0.002486 | 2.53E-05 | 1.005022 |
| C16orf33 | 0.002486 | 2.53E-05 | -1.19564 |
| FLJ39531 | 0.002486 | 2.49E-05 | -1.66556 |
| DLG7 | 0.002486 | 2.50E-05 | -1.66735 |
| GIMAP4 | 0.002486 | 2.41E-05 | -3.30868 |
| ATP6V0E2 | 0.00252 | 2.63E-05 | -1.07404 |
| IBRDC3 | 0.00254 | 2.68E-05 | 1.281204 |
| CXYorf3 | 0.002608 | 2.81E-05 | 1.078268 |
| LOC196752 | 0.002653 | 3.05E-05 | 1.020374 |
| TMEM177 | 0.002653 | 3.03E-05 | -1.09877 |
| CCDC25 | 0.002653 | 3.03E-05 | -1.16734 |
| STX11 | 0.00266 | 3.09E-05 | 1.593416 |
| NIPA1 | 0.002668 | 3.17E-05 | 1.200597 |
| TUT1 | 0.002754 | 3.41E-05 | -1.00172 |
| TRAPPC2L | 0.002774 | 3.53E-05 | -1.05435 |
| RANBP6 | 0.002888 | 3.79E-05 | -1.08145 |
| RBBP9 | 0.003027 | 4.13E-05 | -1.03553 |
| F2RL2 | 0.003033 | 4.19E-05 | -1.138 |
| P2RY5 | 0.003033 | 4.20E-05 | -2.06729 |
| LPXN | 0.003056 | 4.28E-05 | -1.09449 |
| DOLK | 0.003081 | 4.33E-05 | -1.0927 |
| MFNG | 0.003098 | 4.55E-05 | -1.66679 |
| TMEM16A | 0.003122 | 4.67E-05 | 1.570797 |
| RORA | 0.003161 | 4.90E-05 | 1.26979 |
| JUN | 0.003161 | 4.93E-05 | 1.193381 |
| SMARCE1 | 0.003173 | 4.99E-05 | -1.0441 |
| GADD45A | 0.003222 | 5.17E-05 | 1.36158 |
| MAP2K3 | 0.003222 | 5.16E-05 | 1.075567 |
| CLEC14A | 0.00329 | 5.44E-05 | -1.49923 |
| PHLDA1 | 0.003345 | 5.79E-05 | 1.500217 |
| CDK2AP2 | 0.0034 | 5.94E-05 | 1.391734 |
| MT1X | 0.003415 | 6.04E-05 | 4.29973 |
| SOCS4 | 0.003485 | 6.34E-05 | -1.28208 |
| NDRG1 | 0.003517 | 6.60E-05 | 1.199307 |
| PIK3R1 | 0.003517 | 6.63E-05 | -1.59816 |
| ETFB | 0.003541 | 6.77E-05 | -1.12149 |
| CDR2 | 0.00356 | 6.87E-05 | 1.431083 |
| HSPA6 | 0.003621 | 7.24E-05 | 3.606503 |
| CDYL2 | 0.003621 | 7.24E-05 | 1.044156 |
| DUSP23 | 0.003621 | 7.22E-05 | -1.45718 |
| RGC32 | 0.00363 | 7.34E-05 | 1.312455 |
| EIF2AK3 | 0.003729 | 7.88E-05 | 1.26838 |
| UBE2H | 0.003729 | 7.92E-05 | 1.069242 |
| SH2D3C | 0.003771 | 8.13E-05 | -2.01242 |
| ALDH7A1 | 0.003899 | 8.51E-05 | -1.23975 |
| C6orf145 | 0.003909 | 8.59E-05 | 1.011819 |
| CDRT4 | 0.003946 | 8.72E-05 | 1.320447 |
| LOC153222 | 0.003961 | 8.86E-05 | 1.00194 |
| JMY | 0.003984 | 9.29E-05 | 1.116924 |
| RARA | 0.003984 | 9.27E-05 | -1.25551 |
| C1orf63 | 0.004074 | 9.81E-05 | 1.20909 |
| EIF4A2 | 0.004091 | 9.94E-05 | 1.096648 |
| NOX4 | 0.00417 | 0.000103 | -1.43186 |
| NFIL3 | 0.004201 | 0.000106 | 2.021986 |
| SENP5 | 0.004242 | 0.000108 | 1.045818 |
| CRTAC1 | 0.004259 | 0.00011 | 1.227791 |
| MYEOV | 0.004259 | 0.00011 | -1.01802 |
| NR2C2 | 0.00431 | 0.000115 | 1.189071 |
| ISG15 | 0.004318 | 0.000115 | -1.03323 |
| SLC39A14 | 0.004343 | 0.000116 | 1.159295 |
| KLF6 | 0.004343 | 0.000116 | 1.051303 |
| CNKSR3 | 0.004456 | 0.000123 | 1.249923 |
| SLC30A1 | 0.00456 | 0.00013 | 2.315607 |
| KIF20A | 0.004653 | 0.000135 | -1.17421 |
| C1QTNF5 | 0.00469 | 0.000139 | -1.6017 |
| IL6 | 0.004736 | 0.000143 | -1.69372 |
| THBD | 0.004755 | 0.000145 | 1.357328 |
| LXN | 0.004771 | 0.000146 | -1.57114 |
| VGLL4 | 0.004836 | 0.00015 | -1.11983 |
| TOMM34 | 0.00498 | 0.000163 | -1.03453 |
| UBFD1 | 0.00499 | 0.000166 | 1.459028 |
| PUS7 | 0.00499 | 0.000167 | -1.14511 |
| GIMAP7 | 0.004992 | 0.000167 | -2.95672 |
| RPIB9 | 0.005119 | 0.000178 | 1.072396 |
| AXUD1 | 0.005214 | 0.000184 | 1.627784 |
| IL11 | 0.00543 | 0.000199 | 1.341742 |
| LOC728069 | 0.005432 | 0.0002 | -1.10754 |
| PRMT6 | 0.005545 | 0.000208 | -1.16567 |
| MYH10 | 0.005596 | 0.000214 | -1.00054 |
| XPC | 0.005619 | 0.000217 | 1.048831 |
| CASZ1 | 0.005979 | 0.000245 | 1.034084 |
| SMAD5 | 0.006119 | 0.000257 | -1.01521 |
| PIGW | 0.006119 | 0.000257 | -1.09157 |
| RPUSD2 | 0.006335 | 0.000277 | -1.19788 |
| ARL5B | 0.006586 | 0.000295 | 1.015982 |
| KIAA1199 | 0.006756 | 0.000307 | 1.807012 |
| ZFP36 | 0.006856 | 0.000313 | 1.046216 |
| KLHL3 | 0.006896 | 0.000316 | -1.05645 |
| HOXB8 | 0.007489 | 0.000371 | -1.27556 |
| ANGPTL4 | 0.007489 | 0.000371 | -1.97103 |
| FEM1B | 0.007566 | 0.000378 | 1.021108 |
| CDC42EP3 | 0.007626 | 0.000384 | -1.37668 |
| FBXO32 | 0.007911 | 0.000409 | 1.094187 |
| NANS | 0.008001 | 0.000416 | 1.084978 |
| C4orf18 | 0.008075 | 0.000425 | -1.56419 |
| TSPAN6 | 0.008108 | 0.000428 | -1.01213 |
| STMN1 | 0.008139 | 0.000433 | -1.32767 |
| FLJ43339 | 0.008142 | 0.000434 | -1.84717 |
| TK1 | 0.008491 | 0.000468 | -1.15738 |
| ABCG1 | 0.008618 | 0.000484 | -1.29643 |
| SIAH2 | 0.008706 | 0.000494 | 1.192224 |
| TPM2 | 0.008706 | 0.000495 | -1.02053 |
| UBE2L6 | 0.008706 | 0.000493 | -1.06197 |
| RHOBTB3 | 0.008724 | 0.000496 | -1.06729 |
| KLHL21 | 0.008828 | 0.000507 | 1.1834 |
| EGFL7 | 0.00913 | 0.000538 | -1.1285 |
| CLCF1 | 0.009301 | 0.000553 | 1.026041 |
| C20orf108 | 0.009562 | 0.000582 | -1.02522 |
| BAMBI | 0.00959 | 0.000587 | 1.357963 |
| FNBP1L | 0.009884 | 0.000622 | -1.0824 |
| GFPT2 | 0.010151 | 0.000649 | 1.267556 |
| MAT2A | 0.01024 | 0.00066 | -1.17277 |
| HSPA1B | 0.010268 | 0.000662 | 1.669 |
| DIXDC1 | 0.010307 | 0.000668 | -1.14191 |
| SNHG3-RCC1 | 0.01046 | 0.000683 | 1.519624 |
| ALDH1A1 | 0.010502 | 0.000689 | -1.91055 |
| ELL2 | 0.010531 | 0.000698 | 1.342906 |
| HMGB2 | 0.010531 | 0.000701 | 1.062913 |
| HBEGF | 0.010866 | 0.000745 | 1.383201 |
| TMEM158 | 0.011029 | 0.000766 | 1.031998 |
| HYOU1 | 0.01138 | 0.000812 | 1.248446 |
| HOXA5 | 0.01144 | 0.00082 | -1.2244 |
| TMEM46 | 0.01201 | 0.000889 | -1.18212 |
| CTGF | 0.012053 | 0.000893 | -1.43237 |
| MAP2 | 0.012328 | 0.000925 | 1.319452 |
| XBP1 | 0.012617 | 0.000972 | 1.498306 |
| PTPRE | 0.012852 | 0.001001 | -1.12658 |
| THBS1 | 0.012977 | 0.001018 | -2.45001 |
| TRIM22 | 0.013092 | 0.001043 | -1.12684 |
| SFN | 0.013305 | 0.001069 | 1.158853 |
| LIPG | 0.013369 | 0.001078 | -1.82535 |
| GSDML | 0.013566 | 0.001097 | 1.178584 |
| LYSMD2 | 0.013892 | 0.001137 | -1.09303 |
| RYBP | 0.014393 | 0.001203 | 1.04106 |
| IL13RA2 | 0.015274 | 0.001319 | 1.430662 |
| FZD4 | 0.015274 | 0.001319 | -1.10258 |
| TNRC6B | 0.015274 | 0.001322 | -1.21628 |
| OBFC2A | 0.015297 | 0.001325 | -1.32353 |
| HK2 | 0.015677 | 0.001366 | 1.017069 |
| PAFAH1B1 | 0.015677 | 0.001366 | -1.0102 |
| PCDH7 | 0.015756 | 0.001378 | -1.37767 |
| VIP | 0.015889 | 0.001396 | 1.280578 |
| ST3GAL1 | 0.016166 | 0.001433 | 1.007557 |
| CBX2 | 0.016194 | 0.001438 | -1.39366 |
| TICAM2 | 0.016208 | 0.001443 | 1.164544 |
| LOC400566 | 0.016212 | 0.001444 | -1.03958 |
| LOC653994 | 0.016231 | 0.001447 | -1.13758 |
| ANKRD15 | 0.016803 | 0.001528 | 1.130653 |
| ARHGAP28 | 0.016841 | 0.001537 | -1.49974 |
| IRS2 | 0.01724 | 0.001593 | 1.290791 |
| C20orf127 | 0.01737 | 0.001625 | 2.582774 |
| DUSP6 | 0.01738 | 0.001627 | -1.06654 |
| PURB | 0.018155 | 0.001729 | -1.38925 |
| DUSP5 | 0.018254 | 0.001754 | 1.37752 |
| CTSC | 0.018254 | 0.001756 | -1.04275 |
| MT1M | 0.018511 | 0.001792 | 2.780106 |
| ZNF323 | 0.018557 | 0.001798 | -1.6381 |
| ZNF792 | 0.018788 | 0.001831 | -1.03232 |
| BCL6B | 0.019023 | 0.001859 | -1.13114 |
| S100P | 0.019046 | 0.001863 | 1.712901 |
| ACP5 | 0.019086 | 0.001872 | -1.21789 |
| CTHRC1 | 0.019293 | 0.001902 | -1.36589 |
| KCTD12 | 0.0198 | 0.001976 | -1.79886 |
| RIN2 | 0.02045 | 0.002069 | -1.28568 |
| TMEM37 | 0.020878 | 0.002129 | -1.32642 |
| APOL3 | 0.021359 | 0.002208 | -1.72986 |
| TGM2 | 0.021558 | 0.002244 | -1.18169 |
| ATF3 | 0.021757 | 0.002285 | 2.710495 |
| GADD45B | 0.022045 | 0.002337 | 1.252146 |
| MTHFD2 | 0.022126 | 0.00235 | 2.408438 |
| GIMAP8 | 0.022745 | 0.002437 | -1.98234 |
| GRPEL2 | 0.023067 | 0.002487 | -1.07237 |
| RSC1A1 | 0.02314 | 0.002504 | 1.040161 |
| TNFAIP8L1 | 0.023422 | 0.002559 | -1.01689 |
| MT1G | 0.023423 | 0.002563 | 3.052585 |
| GEM | 0.02381 | 0.002639 | 1.143715 |
| TSPYL2 | 0.025563 | 0.002987 | 1.004903 |
| CIRBP | 0.025631 | 0.003004 | -1.00582 |
| MAFB | 0.025741 | 0.00303 | -1.18971 |
| FLJ20366 | 0.025891 | 0.003055 | -1.06536 |
| C20orf160 | 0.025966 | 0.003074 | -1.3568 |
| PTX3 | 0.027328 | 0.003346 | -1.22964 |
| C5orf13 | 0.027335 | 0.003348 | -1.22942 |
| FRMD4A | 0.027834 | 0.003437 | -1.00794 |
| GIMAP1 | 0.028988 | 0.003637 | -1.39722 |
| HSPA5 | 0.029748 | 0.003803 | 1.273662 |
| DNAJB1 | 0.030783 | 0.004018 | 1.077985 |
| CYYR1 | 0.030783 | 0.004019 | -1.01765 |
| FAM107A | 0.031857 | 0.004241 | -1.01981 |
| KCNK1 | 0.033721 | 0.004638 | 1.203102 |
| LOC139886 | 0.034979 | 0.004904 | -1.20717 |
| C12orf48 | 0.034993 | 0.004913 | 1.22901 |
| LDB2 | 0.035291 | 0.004984 | -1.71045 |
| ASNS | 0.037659 | 0.005451 | 2.363438 |
| RPP25 | 0.037793 | 0.005487 | -1.10442 |
| MTUS1 | 0.037811 | 0.005496 | -1.38302 |
| NDRG4 | 0.038093 | 0.005552 | -1.25009 |
| ZNF365 | 0.038814 | 0.005716 | 2.022528 |
| NOV | 0.038825 | 0.005727 | -1.07822 |
| ITGB8 | 0.039145 | 0.005802 | 1.121859 |
| HSPA1A | 0.040084 | 0.005999 | 1.354534 |
| DNAJB9 | 0.040186 | 0.006022 | 1.76515 |
| ERRFI1 | 0.043571 | 0.006745 | 1.048435 |
| RGS4 | 0.043587 | 0.00675 | -1.55189 |
| PHGDH | 0.045719 | 0.007254 | 2.297088 |
| DUSP1 | 0.048207 | 0.007871 | 1.315756 |
| TFPI2 | 0.048693 | 0.007988 | -1.29565 |
| LYN | 0.049387 | 0.008167 | -1.0372 |
| FLJ39575 | 0.049835 | 0.008284 | 1.265421 |

## Supplementary Table2| GO terms and KEGG pathways enriched by 260 DEGs

| ONTOLOGY | ID | Description | p.adjust | qvalue | Count |
| --- | --- | --- | --- | --- | --- |
| BP | GO:0045637 | regulation of myeloid cell differentiation | 5.0623E-06 | 4.2237E-06 | 17 |
| BP | GO:0030099 | myeloid cell differentiation | 5.9002E-06 | 4.9228E-06 | 21 |
| BP | GO:0006986 | response to unfolded protein | 6.3477E-06 | 5.2963E-06 | 14 |
| BP | GO:0051348 | negative regulation of transferase activity | 8.5688E-06 | 7.1494E-06 | 17 |
| BP | GO:0035966 | response to topologically incorrect protein | 1.4906E-05 | 1.2437E-05 | 14 |
| BP | GO:0034620 | cellular response to unfolded protein | 1.4906E-05 | 1.2437E-05 | 12 |
| BP | GO:0045646 | regulation of erythrocyte differentiation | 1.4906E-05 | 1.2437E-05 | 8 |
| BP | GO:0033673 | negative regulation of kinase activity | 4.3125E-05 | 3.5981E-05 | 15 |
| BP | GO:0042326 | negative regulation of phosphorylation | 4.6757E-05 | 3.9012E-05 | 20 |
| BP | GO:1903706 | regulation of hemopoiesis | 4.6757E-05 | 3.9012E-05 | 20 |
| BP | GO:0001933 | negative regulation of protein phosphorylation | 4.6757E-05 | 3.9012E-05 | 19 |
| BP | GO:0035967 | cellular response to topologically incorrect protein | 4.6757E-05 | 3.9012E-05 | 12 |
| BP | GO:0006469 | negative regulation of protein kinase activity | 5.5652E-05 | 4.6433E-05 | 14 |
| BP | GO:0000188 | inactivation of MAPK activity | 7.4837E-05 | 6.2441E-05 | 6 |
| BP | GO:0043409 | negative regulation of MAPK cascade | 0.0001386 | 0.00011564 | 12 |
| BP | GO:0097501 | stress response to metal ion | 0.0001437 | 0.00011989 | 5 |
| BP | GO:0030218 | erythrocyte differentiation | 0.00066757 | 0.00055699 | 9 |
| BP | GO:0002262 | myeloid cell homeostasis | 0.00069389 | 0.00057895 | 10 |
| BP | GO:0030968 | endoplasmic reticulum unfolded protein response | 0.0009279 | 0.0007742 | 9 |
| BP | GO:0045638 | negative regulation of myeloid cell differentiation | 0.0009279 | 0.0007742 | 8 |
| BP | GO:0034101 | erythrocyte homeostasis | 0.00094546 | 0.00078884 | 9 |
| BP | GO:0034976 | response to endoplasmic reticulum stress | 0.00170451 | 0.00142216 | 13 |
| BP | GO:0070373 | negative regulation of ERK1 and ERK2 cascade | 0.0018814 | 0.00156975 | 7 |
| BP | GO:0043405 | regulation of MAP kinase activity | 0.00206031 | 0.00171903 | 14 |
| BP | GO:0032956 | regulation of actin cytoskeleton organization | 0.00235759 | 0.00196706 | 14 |
| BP | GO:0043407 | negative regulation of MAP kinase activity | 0.00235759 | 0.00196706 | 7 |
| BP | GO:0051085 | chaperone cofactor-dependent protein refolding | 0.00243507 | 0.0020317 | 5 |
| BP | GO:0061687 | detoxification of inorganic compound | 0.00302474 | 0.0025237 | 4 |
| BP | GO:1905897 | regulation of response to endoplasmic reticulum stress | 0.00343484 | 0.00286587 | 7 |
| BP | GO:0002683 | negative regulation of immune system process | 0.00375948 | 0.00313673 | 16 |
| BP | GO:0061077 | chaperone-mediated protein folding | 0.00375948 | 0.00313673 | 6 |
| BP | GO:0006882 | cellular zinc ion homeostasis | 0.00375948 | 0.00313673 | 5 |
| BP | GO:0051084 | 'de novo' posttranslational protein folding | 0.00413104 | 0.00344674 | 5 |
| BP | GO:0051098 | regulation of binding | 0.0044347 | 0.0037001 | 14 |
| BP | GO:0055069 | zinc ion homeostasis | 0.00444674 | 0.00371014 | 5 |
| BP | GO:0036499 | PERK-mediated unfolded protein response | 0.00572259 | 0.00477465 | 4 |
| BP | GO:0032970 | regulation of actin filament-based process | 0.00574469 | 0.00479309 | 14 |
| BP | GO:0048872 | homeostasis of number of cells | 0.00574469 | 0.00479309 | 11 |
| BP | GO:1990823 | response to leukemia inhibitory factor | 0.00574469 | 0.00479309 | 7 |
| BP | GO:1990830 | cellular response to leukemia inhibitory factor | 0.00574469 | 0.00479309 | 7 |
| BP | GO:0006458 | 'de novo' protein folding | 0.00574469 | 0.00479309 | 5 |
| BP | GO:0071300 | cellular response to retinoic acid | 0.00698016 | 0.00582391 | 6 |
| BP | GO:1903573 | negative regulation of response to endoplasmic reticulum stress | 0.00743772 | 0.00620567 | 5 |
| BP | GO:1902903 | regulation of supramolecular fiber organization | 0.00754093 | 0.00629178 | 13 |
| BP | GO:0035335 | peptidyl-tyrosine dephosphorylation | 0.0076874 | 0.00641399 | 7 |
| BP | GO:2001233 | regulation of apoptotic signaling pathway | 0.00808044 | 0.00674193 | 14 |
| BP | GO:0006984 | ER-nucleus signaling pathway | 0.00844274 | 0.00704421 | 5 |
| BP | GO:1900101 | regulation of endoplasmic reticulum unfolded protein response | 0.00877525 | 0.00732164 | 4 |
| BP | GO:0032526 | response to retinoic acid | 0.00990331 | 0.00826284 | 7 |
| BP | GO:0002762 | negative regulation of myeloid leukocyte differentiation | 0.00990331 | 0.00826284 | 5 |
| BP | GO:0071901 | negative regulation of protein serine/threonine kinase activity | 0.01014675 | 0.00846596 | 8 |
| BP | GO:0090084 | negative regulation of inclusion body assembly | 0.01131256 | 0.00943865 | 3 |
| BP | GO:0038066 | p38MAPK cascade | 0.01448774 | 0.01208787 | 5 |
| BP | GO:0097201 | negative regulation of transcription from RNA polymerase II promoter in response to stress | 0.01448774 | 0.01208787 | 3 |
| BP | GO:1903707 | negative regulation of hemopoiesis | 0.01458772 | 0.01217128 | 8 |
| BP | GO:2001236 | regulation of extrinsic apoptotic signaling pathway | 0.01458772 | 0.01217128 | 8 |
| BP | GO:0009266 | response to temperature stimulus | 0.01574759 | 0.01313903 | 10 |
| BP | GO:0008625 | extrinsic apoptotic signaling pathway via death domain receptors | 0.01672983 | 0.01395856 | 6 |
| BP | GO:0045648 | positive regulation of erythrocyte differentiation | 0.01672983 | 0.01395856 | 4 |
| BP | GO:1903960 | negative regulation of anion transmembrane transport | 0.01672983 | 0.01395856 | 3 |
| BP | GO:0032434 | regulation of proteasomal ubiquitin-dependent protein catabolic process | 0.01721892 | 0.01436663 | 7 |
| BP | GO:1902235 | regulation of endoplasmic reticulum stress-induced intrinsic apoptotic signaling pathway | 0.01822415 | 0.01520534 | 4 |
| BP | GO:1902041 | regulation of extrinsic apoptotic signaling pathway via death domain receptors | 0.01899748 | 0.01585058 | 5 |
| BP | GO:0007175 | negative regulation of epidermal growth factor-activated receptor activity | 0.0198061 | 0.01652525 | 3 |
| BP | GO:0045639 | positive regulation of myeloid cell differentiation | 0.0206839 | 0.01725764 | 6 |
| BP | GO:0048660 | regulation of smooth muscle cell proliferation | 0.02214129 | 0.01847362 | 8 |
| BP | GO:0031331 | positive regulation of cellular catabolic process | 0.02240185 | 0.01869101 | 12 |
| BP | GO:1903050 | regulation of proteolysis involved in cellular protein catabolic process | 0.02240185 | 0.01869101 | 9 |
| BP | GO:0048659 | smooth muscle cell proliferation | 0.02240185 | 0.01869101 | 8 |
| BP | GO:0010273 | detoxification of copper ion | 0.02240185 | 0.01869101 | 3 |
| BP | GO:1990169 | stress response to copper ion | 0.02240185 | 0.01869101 | 3 |
| BP | GO:0030183 | B cell differentiation | 0.02243954 | 0.01872247 | 7 |
| BP | GO:0032271 | regulation of protein polymerization | 0.02386911 | 0.01991522 | 9 |
| BP | GO:0046686 | response to cadmium ion | 0.02386911 | 0.01991522 | 5 |
| BP | GO:0009896 | positive regulation of catabolic process | 0.0244162 | 0.02037169 | 13 |
| BP | GO:0031668 | cellular response to extracellular stimulus | 0.0244162 | 0.02037169 | 10 |
| BP | GO:0051591 | response to cAMP | 0.0244162 | 0.02037169 | 6 |
| BP | GO:0036498 | IRE1-mediated unfolded protein response | 0.0244162 | 0.02037169 | 5 |
| BP | GO:0002070 | epithelial cell maturation | 0.0244162 | 0.02037169 | 3 |
| BP | GO:0090083 | regulation of inclusion body assembly | 0.0244162 | 0.02037169 | 3 |
| BP | GO:0071276 | cellular response to cadmium ion | 0.02458193 | 0.02050997 | 4 |
| BP | GO:0097191 | extrinsic apoptotic signaling pathway | 0.0255468 | 0.02131501 | 9 |
| BP | GO:0051099 | positive regulation of binding | 0.0255468 | 0.02131501 | 8 |
| BP | GO:0034605 | cellular response to heat | 0.0255468 | 0.02131501 | 7 |
| BP | GO:0050678 | regulation of epithelial cell proliferation | 0.02773585 | 0.02314145 | 12 |
| BP | GO:0061136 | regulation of proteasomal protein catabolic process | 0.02773585 | 0.02314145 | 8 |
| BP | GO:0021549 | cerebellum development | 0.02773585 | 0.02314145 | 6 |
| BP | GO:0055090 | acylglycerol homeostasis | 0.02773585 | 0.02314145 | 4 |
| BP | GO:0070328 | triglyceride homeostasis | 0.02773585 | 0.02314145 | 4 |
| BP | GO:2001234 | negative regulation of apoptotic signaling pathway | 0.02877554 | 0.02400891 | 9 |
| BP | GO:1903708 | positive regulation of hemopoiesis | 0.02888266 | 0.02409828 | 8 |
| BP | GO:0070507 | regulation of microtubule cytoskeleton organization | 0.02888266 | 0.02409828 | 8 |
| BP | GO:2001237 | negative regulation of extrinsic apoptotic signaling pathway | 0.02888266 | 0.02409828 | 6 |
| BP | GO:0042026 | protein refolding | 0.02888266 | 0.02409828 | 4 |
| BP | GO:0042036 | negative regulation of cytokine biosynthetic process | 0.02888266 | 0.02409828 | 4 |
| BP | GO:0051258 | protein polymerization | 0.03063688 | 0.02556192 | 10 |
| BP | GO:0071216 | cellular response to biotic stimulus | 0.03201839 | 0.02671459 | 9 |
| BP | GO:0031669 | cellular response to nutrient levels | 0.0326467 | 0.02723882 | 9 |
| BP | GO:0042149 | cellular response to glucose starvation | 0.03270724 | 0.02728933 | 4 |
| BP | GO:0071496 | cellular response to external stimulus | 0.03307266 | 0.02759422 | 11 |
| BP | GO:0033002 | muscle cell proliferation | 0.03359833 | 0.02803281 | 9 |
| BP | GO:2000058 | regulation of ubiquitin-dependent protein catabolic process | 0.0339298 | 0.02830937 | 7 |
| BP | GO:0007015 | actin filament organization | 0.03631765 | 0.03030168 | 12 |
| BP | GO:0030902 | hindbrain development | 0.03631765 | 0.03030168 | 7 |
| BP | GO:0022037 | metencephalon development | 0.03631765 | 0.03030168 | 6 |
| BP | GO:0046688 | response to copper ion | 0.03631765 | 0.03030168 | 4 |
| BP | GO:1902236 | negative regulation of endoplasmic reticulum stress-induced intrinsic apoptotic signaling pathway | 0.03631765 | 0.03030168 | 3 |
| BP | GO:1903362 | regulation of cellular protein catabolic process | 0.03951289 | 0.03296763 | 9 |
| BP | GO:0042035 | regulation of cytokine biosynthetic process | 0.0400116 | 0.03338374 | 6 |
| BP | GO:0032891 | negative regulation of organic acid transport | 0.0400116 | 0.03338374 | 3 |
| BP | GO:0030098 | lymphocyte differentiation | 0.0404664 | 0.0337632 | 11 |
| BP | GO:0070372 | regulation of ERK1 and ERK2 cascade | 0.0404664 | 0.0337632 | 10 |
| BP | GO:0019318 | hexose metabolic process | 0.0404664 | 0.0337632 | 9 |
| BP | GO:0002573 | myeloid leukocyte differentiation | 0.04274033 | 0.03566045 | 8 |
| BP | GO:0031396 | regulation of protein ubiquitination | 0.04274033 | 0.03566045 | 8 |
| BP | GO:0021695 | cerebellar cortex development | 0.04274033 | 0.03566045 | 4 |
| BP | GO:0002761 | regulation of myeloid leukocyte differentiation | 0.0429787 | 0.03585934 | 6 |
| BP | GO:0050769 | positive regulation of neurogenesis | 0.04480787 | 0.03738551 | 13 |
| BP | GO:0018108 | peptidyl-tyrosine phosphorylation | 0.04480787 | 0.03738551 | 11 |
| BP | GO:0010038 | response to metal ion | 0.04480787 | 0.03738551 | 11 |
| BP | GO:0042113 | B cell activation | 0.04480787 | 0.03738551 | 10 |
| BP | GO:0050730 | regulation of peptidyl-tyrosine phosphorylation | 0.04480787 | 0.03738551 | 9 |
| BP | GO:0043406 | positive regulation of MAP kinase activity | 0.04480787 | 0.03738551 | 9 |
| BP | GO:0071229 | cellular response to acid chemical | 0.04480787 | 0.03738551 | 8 |
| BP | GO:1903052 | positive regulation of proteolysis involved in cellular protein catabolic process | 0.04480787 | 0.03738551 | 6 |
| BP | GO:0032436 | positive regulation of proteasomal ubiquitin-dependent protein catabolic process | 0.04480787 | 0.03738551 | 5 |
| BP | GO:0042059 | negative regulation of epidermal growth factor receptor signaling pathway | 0.04480787 | 0.03738551 | 4 |
| BP | GO:0006541 | glutamine metabolic process | 0.04480787 | 0.03738551 | 3 |
| BP | GO:0070841 | inclusion body assembly | 0.04480787 | 0.03738551 | 3 |
| BP | GO:0071294 | cellular response to zinc ion | 0.04480787 | 0.03738551 | 3 |
| BP | GO:0018212 | peptidyl-tyrosine modification | 0.0464144 | 0.03872592 | 11 |
| BP | GO:0110053 | regulation of actin filament organization | 0.0465478 | 0.03883722 | 9 |
| BP | GO:0001961 | positive regulation of cytokine-mediated signaling pathway | 0.0465478 | 0.03883722 | 4 |
| BP | GO:0031113 | regulation of microtubule polymerization | 0.0465478 | 0.03883722 | 4 |
| BP | GO:0031098 | stress-activated protein kinase signaling cascade | 0.04765368 | 0.03975991 | 10 |
| BP | GO:0042089 | cytokine biosynthetic process | 0.04765368 | 0.03975991 | 6 |
| BP | GO:0043154 | negative regulation of cysteine-type endopeptidase activity involved in apoptotic process | 0.04765368 | 0.03975991 | 5 |
| BP | GO:0008637 | apoptotic mitochondrial changes | 0.04860135 | 0.04055061 | 6 |
| BP | GO:0042107 | cytokine metabolic process | 0.04860135 | 0.04055061 | 6 |
| BP | GO:0070371 | ERK1 and ERK2 cascade | 0.0488319 | 0.04074296 | 10 |
| BP | GO:0051100 | negative regulation of binding | 0.05053263 | 0.04216197 | 7 |
| BP | GO:0043393 | regulation of protein binding | 0.05139131 | 0.04287842 | 8 |
| BP | GO:0021680 | cerebellar Purkinje cell layer development | 0.05140325 | 0.04288838 | 3 |
| BP | GO:0032886 | regulation of microtubule-based process | 0.05214084 | 0.04350378 | 8 |
| BP | GO:0048732 | gland development | 0.05228091 | 0.04362065 | 12 |
| BP | GO:0050673 | epithelial cell proliferation | 0.05228091 | 0.04362065 | 12 |
| BP | GO:0070228 | regulation of lymphocyte apoptotic process | 0.05228091 | 0.04362065 | 4 |
| BP | GO:1901185 | negative regulation of ERBB signaling pathway | 0.05228091 | 0.04362065 | 4 |
| BP | GO:0034763 | negative regulation of transmembrane transport | 0.05318738 | 0.04437697 | 6 |
| BP | GO:0032868 | response to insulin | 0.05460301 | 0.0455581 | 9 |
| BP | GO:1903959 | regulation of anion transmembrane transport | 0.05460301 | 0.0455581 | 3 |
| BP | GO:0031667 | response to nutrient levels | 0.05636133 | 0.04702515 | 13 |
| BP | GO:0043030 | regulation of macrophage activation | 0.05794203 | 0.04834401 | 4 |
| BP | GO:0048545 | response to steroid hormone | 0.05800939 | 0.04840022 | 11 |
| BP | GO:0009408 | response to heat | 0.05800939 | 0.04840022 | 7 |
| BP | GO:0032496 | response to lipopolysaccharide | 0.05855894 | 0.04885874 | 10 |
| BP | GO:0007176 | regulation of epidermal growth factor-activated receptor activity | 0.05862319 | 0.04891235 | 3 |
| BP | GO:2000117 | negative regulation of cysteine-type endopeptidase activity | 0.06084887 | 0.05076934 | 5 |
| BP | GO:1903034 | regulation of response to wounding | 0.0617964 | 0.05155992 | 7 |
| BP | GO:0009314 | response to radiation | 0.06184014 | 0.05159641 | 12 |
| BP | GO:0046683 | response to organophosphorus | 0.06184014 | 0.05159641 | 6 |
| BP | GO:0060760 | positive regulation of response to cytokine stimulus | 0.06234803 | 0.05202016 | 4 |
| BP | GO:0042730 | fibrinolysis | 0.06236891 | 0.05203759 | 3 |
| BP | GO:1903792 | negative regulation of anion transport | 0.06236891 | 0.05203759 | 3 |
| BP | GO:1903320 | regulation of protein modification by small protein conjugation or removal | 0.06481868 | 0.05408156 | 8 |
| BP | GO:0071385 | cellular response to glucocorticoid stimulus | 0.06481868 | 0.05408156 | 4 |
| BP | GO:0072593 | reactive oxygen species metabolic process | 0.06534172 | 0.05451796 | 9 |
| BP | GO:0042116 | macrophage activation | 0.06534172 | 0.05451796 | 5 |
| BP | GO:2000060 | positive regulation of ubiquitin-dependent protein catabolic process | 0.06534172 | 0.05451796 | 5 |
| BP | GO:0010575 | positive regulation of vascular endothelial growth factor production | 0.0658165 | 0.05491409 | 3 |
| BP | GO:0061099 | negative regulation of protein tyrosine kinase activity | 0.0658165 | 0.05491409 | 3 |
| BP | GO:0071280 | cellular response to copper ion | 0.0658165 | 0.05491409 | 3 |
| BP | GO:0051403 | stress-activated MAPK cascade | 0.06630062 | 0.05531801 | 9 |
| BP | GO:0030219 | megakaryocyte differentiation | 0.06638591 | 0.05538918 | 5 |
| BP | GO:0001101 | response to acid chemical | 0.06826673 | 0.05695844 | 10 |
| BP | GO:0002237 | response to molecule of bacterial origin | 0.06826673 | 0.05695844 | 10 |
| BP | GO:0045576 | mast cell activation | 0.0687328 | 0.05734731 | 4 |
| BP | GO:1903364 | positive regulation of cellular protein catabolic process | 0.0694018 | 0.05790549 | 6 |
| BP | GO:0071384 | cellular response to corticosteroid stimulus | 0.0713434 | 0.05952547 | 4 |
| BP | GO:0090303 | positive regulation of wound healing | 0.0713434 | 0.05952547 | 4 |
| BP | GO:1905953 | negative regulation of lipid localization | 0.0713434 | 0.05952547 | 4 |
| BP | GO:0005996 | monosaccharide metabolic process | 0.07217514 | 0.06021943 | 9 |
| BP | GO:0030879 | mammary gland development | 0.07446291 | 0.06212824 | 6 |
| BP | GO:0070059 | intrinsic apoptotic signaling pathway in response to endoplasmic reticulum stress | 0.07446291 | 0.06212824 | 4 |
| BP | GO:0048511 | rhythmic process | 0.0754672 | 0.06296616 | 9 |
| BP | GO:0042594 | response to starvation | 0.0754672 | 0.06296616 | 7 |
| BP | GO:0001818 | negative regulation of cytokine production | 0.07631899 | 0.06367686 | 9 |
| BP | GO:0048661 | positive regulation of smooth muscle cell proliferation | 0.07631899 | 0.06367686 | 5 |
| BP | GO:0002637 | regulation of immunoglobulin production | 0.07671968 | 0.06401117 | 4 |
| BP | GO:0001505 | regulation of neurotransmitter levels | 0.07770002 | 0.06482912 | 10 |
| BP | GO:2000377 | regulation of reactive oxygen species metabolic process | 0.07770002 | 0.06482912 | 7 |
| BP | GO:0051384 | response to glucocorticoid | 0.07770002 | 0.06482912 | 6 |
| BP | GO:1901657 | glycosyl compound metabolic process | 0.07770002 | 0.06482912 | 6 |
| BP | GO:2000379 | positive regulation of reactive oxygen species metabolic process | 0.07770002 | 0.06482912 | 5 |
| BP | GO:0032091 | negative regulation of protein binding | 0.07770002 | 0.06482912 | 5 |
| BP | GO:0034766 | negative regulation of ion transmembrane transport | 0.07770002 | 0.06482912 | 5 |
| BP | GO:1902106 | negative regulation of leukocyte differentiation | 0.07770002 | 0.06482912 | 5 |
| BP | GO:0071353 | cellular response to interleukin-4 | 0.07770002 | 0.06482912 | 3 |
| BP | GO:0036500 | ATF6-mediated unfolded protein response | 0.07770002 | 0.06482912 | 2 |
| BP | GO:0051918 | negative regulation of fibrinolysis | 0.07770002 | 0.06482912 | 2 |
| BP | GO:1903265 | positive regulation of tumor necrosis factor-mediated signaling pathway | 0.07770002 | 0.06482912 | 2 |
| BP | GO:2000574 | regulation of microtubule motor activity | 0.07770002 | 0.06482912 | 2 |
| BP | GO:0010498 | proteasomal protein catabolic process | 0.07915096 | 0.06603971 | 12 |
| BP | GO:0061041 | regulation of wound healing | 0.07915096 | 0.06603971 | 6 |
| BP | GO:0071887 | leukocyte apoptotic process | 0.07915096 | 0.06603971 | 5 |
| BP | GO:1901800 | positive regulation of proteasomal protein catabolic process | 0.07915096 | 0.06603971 | 5 |
| BP | GO:0045926 | negative regulation of growth | 0.08026907 | 0.06697261 | 8 |
| BP | GO:0009267 | cellular response to starvation | 0.08026907 | 0.06697261 | 6 |
| BP | GO:0014074 | response to purine-containing compound | 0.08026907 | 0.06697261 | 6 |
| BP | GO:0043161 | proteasome-mediated ubiquitin-dependent protein catabolic process | 0.08068082 | 0.06731615 | 11 |
| BP | GO:0045861 | negative regulation of proteolysis | 0.08294819 | 0.06920794 | 10 |
| BP | GO:0055088 | lipid homeostasis | 0.08294819 | 0.06920794 | 6 |
| BP | GO:0010574 | regulation of vascular endothelial growth factor production | 0.08294819 | 0.06920794 | 3 |
| BP | GO:0033120 | positive regulation of RNA splicing | 0.08294819 | 0.06920794 | 3 |
| BP | GO:0070670 | response to interleukin-4 | 0.08294819 | 0.06920794 | 3 |
| BP | GO:0001678 | cellular glucose homeostasis | 0.08411065 | 0.07017784 | 6 |
| BP | GO:0043502 | regulation of muscle adaptation | 0.08411065 | 0.07017784 | 5 |
| BP | GO:0000185 | activation of MAPKKK activity | 0.08411065 | 0.07017784 | 2 |
| BP | GO:0051024 | positive regulation of immunoglobulin secretion | 0.08411065 | 0.07017784 | 2 |
| BP | GO:0055091 | phospholipid homeostasis | 0.08411065 | 0.07017784 | 2 |
| BP | GO:1900102 | negative regulation of endoplasmic reticulum unfolded protein response | 0.08411065 | 0.07017784 | 2 |
| BP | GO:1990440 | positive regulation of transcription from RNA polymerase II promoter in response to endoplasmic reticulum stress | 0.08411065 | 0.07017784 | 2 |
| BP | GO:0042133 | neurotransmitter metabolic process | 0.08458158 | 0.07057076 | 6 |
| BP | GO:0046916 | cellular transition metal ion homeostasis | 0.08458158 | 0.07057076 | 5 |
| BP | GO:0010812 | negative regulation of cell-substrate adhesion | 0.08458158 | 0.07057076 | 4 |
| BP | GO:1902905 | positive regulation of supramolecular fiber organization | 0.08826499 | 0.07364402 | 7 |
| BP | GO:0070227 | lymphocyte apoptotic process | 0.08826499 | 0.07364402 | 4 |
| BP | GO:0071222 | cellular response to lipopolysaccharide | 0.08996425 | 0.0750618 | 7 |
| BP | GO:0045666 | positive regulation of neuron differentiation | 0.09020233 | 0.07526045 | 10 |
| BP | GO:0010573 | vascular endothelial growth factor production | 0.09109618 | 0.07600623 | 3 |
| BP | GO:0043271 | negative regulation of ion transport | 0.09292367 | 0.077531 | 6 |
| BP | GO:0002064 | epithelial cell development | 0.093079 | 0.07766059 | 7 |
| BP | GO:0006983 | ER overload response | 0.09349342 | 0.07800636 | 2 |
| BP | GO:0060213 | positive regulation of nuclear-transcribed mRNA poly(A) tail shortening | 0.09349342 | 0.07800636 | 2 |
| BP | GO:0070493 | thrombin-activated receptor signaling pathway | 0.09349342 | 0.07800636 | 2 |
| BP | GO:0098974 | postsynaptic actin cytoskeleton organization | 0.09349342 | 0.07800636 | 2 |
| BP | GO:1903894 | regulation of IRE1-mediated unfolded protein response | 0.09349342 | 0.07800636 | 2 |
| BP | GO:1904424 | regulation of GTP binding | 0.09349342 | 0.07800636 | 2 |
| BP | GO:0021587 | cerebellum morphogenesis | 0.09466589 | 0.07898462 | 3 |
| BP | GO:0032680 | regulation of tumor necrosis factor production | 0.09781395 | 0.08161121 | 6 |
| BP | GO:0002688 | regulation of leukocyte chemotaxis | 0.09885974 | 0.08248377 | 5 |
| KEGG | hsa04010 | MAPK signaling pathway | 0.01770152 | 0.01547364 | 13 |
| KEGG | hsa04141 | Protein processing in endoplasmic reticulum | 0.03375117 | 0.02950332 | 9 |
| KEGG | hsa04068 | FoxO signaling pathway | 0.05241262 | 0.04581607 | 7 |
| KEGG | hsa04210 | Apoptosis | 0.05241262 | 0.04581607 | 7 |
| KEGG | hsa04915 | Estrogen signaling pathway | 0.05241262 | 0.04581607 | 7 |
| KEGG | hsa05162 | Measles | 0.05241262 | 0.04581607 | 7 |
| KEGG | hsa04933 | AGE-RAGE signaling pathway in diabetic complications | 0.05241262 | 0.04581607 | 6 |
| KEGG | hsa04213 | Longevity regulating pathway - multiple species | 0.05241262 | 0.04581607 | 5 |
| KEGG | hsa04931 | Insulin resistance | 0.05841528 | 0.05106324 | 6 |
| KEGG | hsa04612 | Antigen processing and presentation | 0.05841528 | 0.05106324 | 5 |
| KEGG | hsa04930 | Type II diabetes mellitus | 0.05841528 | 0.05106324 | 4 |
| KEGG | hsa00520 | Amino sugar and nucleotide sugar metabolism | 0.05841528 | 0.05106324 | 4 |
| KEGG | hsa05169 | Epstein-Barr virus infection | 0.06931365 | 0.06058997 | 8 |
| KEGG | hsa05323 | Rheumatoid arthritis | 0.09220545 | 0.08060065 | 5 |
| KEGG | hsa05134 | Legionellosis | 0.09220545 | 0.08060065 | 4 |
| KEGG | hsa04978 | Mineral absorption | 0.09220545 | 0.08060065 | 4 |
| MF | GO:0017017 | MAP kinase tyrosine/serine/threonine phosphatase activity | 0.00012501 | 0.00011456 | 5 |
| MF | GO:0033549 | MAP kinase phosphatase activity | 0.00014306 | 0.0001311 | 5 |
| MF | GO:0031072 | heat shock protein binding | 0.00134205 | 0.00122983 | 9 |
| MF | GO:0008138 | protein tyrosine/serine/threonine phosphatase activity | 0.00134205 | 0.00122983 | 6 |
| MF | GO:0051787 | misfolded protein binding | 0.00134205 | 0.00122983 | 5 |
| MF | GO:0003714 | transcription corepressor activity | 0.00960331 | 0.00880031 | 11 |
| MF | GO:0044183 | protein binding involved in protein folding | 0.00960331 | 0.00880031 | 4 |
| MF | GO:0004725 | protein tyrosine phosphatase activity | 0.01097452 | 0.01005686 | 7 |
| MF | GO:0043015 | gamma-tubulin binding | 0.01196636 | 0.01096576 | 4 |
| MF | GO:0050786 | RAGE receptor binding | 0.01196636 | 0.01096576 | 3 |
| MF | GO:0051219 | phosphoprotein binding | 0.01948928 | 0.01785964 | 6 |
| MF | GO:0050839 | cell adhesion molecule binding | 0.03566947 | 0.03268689 | 15 |
| MF | GO:0051087 | chaperone binding | 0.04889877 | 0.04480999 | 6 |
| MF | GO:0001221 | transcription cofactor binding | 0.05565311 | 0.05099955 | 4 |
| MF | GO:0045296 | cadherin binding | 0.063448 | 0.05814266 | 11 |
| MF | GO:0001223 | transcription coactivator binding | 0.07231228 | 0.06626573 | 3 |

## Supplementary Table 3| GO terms and KEGG pathways enriched by top 10 hub genes

| ONTOLOGY | ID | Description | p.adjust | qvalue | Count |
| --- | --- | --- | --- | --- | --- |
| BP | GO:0034620 | cellular response to unfolded protein | 3.0447E-08 | 1.0637E-08 | 6 |
| BP | GO:0035967 | cellular response to topologically incorrect protein | 3.5583E-08 | 1.2431E-08 | 6 |
| BP | GO:0006986 | response to unfolded protein | 4.0699E-08 | 1.4219E-08 | 6 |
| BP | GO:0035966 | response to topologically incorrect protein | 6.4151E-08 | 2.2413E-08 | 6 |
| BP | GO:0051085 | chaperone cofactor-dependent protein refolding | 2.7584E-07 | 9.6371E-08 | 4 |
| BP | GO:0051084 | 'de novo' posttranslational protein folding | 4.2164E-07 | 1.4731E-07 | 4 |
| BP | GO:0042026 | protein refolding | 4.8439E-07 | 1.6923E-07 | 4 |
| BP | GO:0006458 | 'de novo' protein folding | 4.8439E-07 | 1.6923E-07 | 4 |
| BP | GO:0061077 | chaperone-mediated protein folding | 1.9261E-06 | 6.7293E-07 | 4 |
| BP | GO:0006457 | protein folding | 5.6769E-06 | 1.9833E-06 | 5 |
| BP | GO:0034976 | response to endoplasmic reticulum stress | 1.6035E-05 | 5.6022E-06 | 5 |
| BP | GO:0043618 | regulation of transcription from RNA polymerase II promoter in response to stress | 2.6537E-05 | 9.2712E-06 | 4 |
| BP | GO:0043620 | regulation of DNA-templated transcription in response to stress | 2.9753E-05 | 1.0395E-05 | 4 |
| BP | GO:0034605 | cellular response to heat | 3.7447E-05 | 1.3083E-05 | 4 |
| BP | GO:0009267 | cellular response to starvation | 4.8924E-05 | 1.7093E-05 | 4 |
| BP | GO:1903573 | negative regulation of response to endoplasmic reticulum stress | 8.3977E-05 | 2.9339E-05 | 3 |
| BP | GO:0009408 | response to heat | 8.3977E-05 | 2.9339E-05 | 4 |
| BP | GO:0006984 | ER-nucleus signaling pathway | 8.5835E-05 | 2.9988E-05 | 3 |
| BP | GO:0006979 | response to oxidative stress | 8.7083E-05 | 3.0424E-05 | 5 |
| BP | GO:1903708 | positive regulation of hemopoiesis | 8.7083E-05 | 3.0424E-05 | 4 |
| BP | GO:0042594 | response to starvation | 9.4182E-05 | 3.2904E-05 | 4 |
| BP | GO:0031669 | cellular response to nutrient levels | 0.0002119 | 7.403E-05 | 4 |
| BP | GO:0009266 | response to temperature stimulus | 0.0002238 | 7.8187E-05 | 4 |
| BP | GO:0031668 | cellular response to extracellular stimulus | 0.00031599 | 0.0001104 | 4 |
| BP | GO:1905897 | regulation of response to endoplasmic reticulum stress | 0.00038379 | 0.00013408 | 3 |
| BP | GO:0036500 | ATF6-mediated unfolded protein response | 0.00039974 | 0.00013966 | 2 |
| BP | GO:1903265 | positive regulation of tumor necrosis factor-mediated signaling pathway | 0.00039974 | 0.00013966 | 2 |
| BP | GO:0034599 | cellular response to oxidative stress | 0.00041212 | 0.00014398 | 4 |
| BP | GO:0045639 | positive regulation of myeloid cell differentiation | 0.00041212 | 0.00014398 | 3 |
| BP | GO:0051024 | positive regulation of immunoglobulin secretion | 0.00041212 | 0.00014398 | 2 |
| BP | GO:0090084 | negative regulation of inclusion body assembly | 0.00041212 | 0.00014398 | 2 |
| BP | GO:1990440 | positive regulation of transcription from RNA polymerase II promoter in response to endoplasmic reticulum stress | 0.00041212 | 0.00014398 | 2 |
| BP | GO:0051591 | response to cAMP | 0.00044825 | 0.00015661 | 3 |
| BP | GO:0097201 | negative regulation of transcription from RNA polymerase II promoter in response to stress | 0.00046532 | 0.00016257 | 2 |
| BP | GO:0046677 | response to antibiotic | 0.00047488 | 0.00016591 | 4 |
| BP | GO:0070431 | nucleotide-binding oligomerization domain containing 2 signaling pathway | 0.00051759 | 0.00018083 | 2 |
| BP | GO:0071496 | cellular response to external stimulus | 0.00051759 | 0.00018083 | 4 |
| BP | GO:0030968 | endoplasmic reticulum unfolded protein response | 0.00073673 | 0.00025739 | 3 |
| BP | GO:0090083 | regulation of inclusion body assembly | 0.00073673 | 0.00025739 | 2 |
| BP | GO:0051023 | regulation of immunoglobulin secretion | 0.00091532 | 0.00031978 | 2 |
| BP | GO:2001233 | regulation of apoptotic signaling pathway | 0.00092687 | 0.00032382 | 4 |
| BP | GO:0046683 | response to organophosphorus | 0.00092687 | 0.00032382 | 3 |
| BP | GO:1901673 | regulation of mitotic spindle assembly | 0.00103275 | 0.00036081 | 2 |
| BP | GO:1902236 | negative regulation of endoplasmic reticulum stress-induced intrinsic apoptotic signaling pathway | 0.00103275 | 0.00036081 | 2 |
| BP | GO:0051090 | regulation of DNA-binding transcription factor activity | 0.00106831 | 0.00037323 | 4 |
| BP | GO:0036499 | PERK-mediated unfolded protein response | 0.00106831 | 0.00037323 | 2 |
| BP | GO:0042542 | response to hydrogen peroxide | 0.00106831 | 0.00037323 | 3 |
| BP | GO:0002262 | myeloid cell homeostasis | 0.00106831 | 0.00037323 | 3 |
| BP | GO:0051592 | response to calcium ion | 0.00106831 | 0.00037323 | 3 |
| BP | GO:0014074 | response to purine-containing compound | 0.00106831 | 0.00037323 | 3 |
| BP | GO:0048305 | immunoglobulin secretion | 0.00108265 | 0.00037824 | 2 |
| BP | GO:2001236 | regulation of extrinsic apoptotic signaling pathway | 0.00114068 | 0.00039852 | 3 |
| BP | GO:0070841 | inclusion body assembly | 0.00114068 | 0.00039852 | 2 |
| BP | GO:0001819 | positive regulation of cytokine production | 0.00121059 | 0.00042294 | 4 |
| BP | GO:0007088 | regulation of mitotic nuclear division | 0.00125695 | 0.00043914 | 3 |
| BP | GO:1903706 | regulation of hemopoiesis | 0.00125695 | 0.00043914 | 4 |
| BP | GO:1900101 | regulation of endoplasmic reticulum unfolded protein response | 0.00125695 | 0.00043914 | 2 |
| BP | GO:0007020 | microtubule nucleation | 0.00133784 | 0.0004674 | 2 |
| BP | GO:0090169 | regulation of spindle assembly | 0.00141997 | 0.00049609 | 2 |
| BP | GO:0002446 | neutrophil mediated immunity | 0.00142172 | 0.00049671 | 4 |
| BP | GO:0031667 | response to nutrient levels | 0.00142172 | 0.00049671 | 4 |
| BP | GO:0001959 | regulation of cytokine-mediated signaling pathway | 0.00143747 | 0.00050221 | 3 |
| BP | GO:0031116 | positive regulation of microtubule polymerization | 0.00153731 | 0.00053709 | 2 |
| BP | GO:0051783 | regulation of nuclear division | 0.00166514 | 0.00058175 | 3 |
| BP | GO:0060759 | regulation of response to cytokine stimulus | 0.00167972 | 0.00058684 | 3 |
| BP | GO:0045648 | positive regulation of erythrocyte differentiation | 0.00167972 | 0.00058684 | 2 |
| BP | GO:0071353 | cellular response to interleukin-4 | 0.00173851 | 0.00060738 | 2 |
| BP | GO:1902235 | regulation of endoplasmic reticulum stress-induced intrinsic apoptotic signaling pathway | 0.00173851 | 0.00060738 | 2 |
| BP | GO:0031112 | positive regulation of microtubule polymerization or depolymerization | 0.00177621 | 0.00062055 | 2 |
| BP | GO:0036003 | positive regulation of transcription from RNA polymerase II promoter in response to stress | 0.00177621 | 0.00062055 | 2 |
| BP | GO:0007179 | transforming growth factor beta receptor signaling pathway | 0.00177621 | 0.00062055 | 3 |
| BP | GO:0070670 | response to interleukin-4 | 0.00185603 | 0.00064844 | 2 |
| BP | GO:0031396 | regulation of protein ubiquitination | 0.00185913 | 0.00064952 | 3 |
| BP | GO:0070423 | nucleotide-binding oligomerization domain containing signaling pathway | 0.00191477 | 0.00066896 | 2 |
| BP | GO:0035872 | nucleotide-binding domain, leucine rich repeat containing receptor signaling pathway | 0.00194786 | 0.00068052 | 2 |
| BP | GO:1901099 | negative regulation of signal transduction in absence of ligand | 0.00194786 | 0.00068052 | 2 |
| BP | GO:2001240 | negative regulation of extrinsic apoptotic signaling pathway in absence of ligand | 0.00194786 | 0.00068052 | 2 |
| BP | GO:0060236 | regulation of mitotic spindle organization | 0.00203218 | 0.00070998 | 2 |
| BP | GO:0097191 | extrinsic apoptotic signaling pathway | 0.00226453 | 0.00079116 | 3 |
| BP | GO:2001234 | negative regulation of apoptotic signaling pathway | 0.00238892 | 0.00083461 | 3 |
| BP | GO:0090224 | regulation of spindle organization | 0.00238892 | 0.00083461 | 2 |
| BP | GO:1903320 | regulation of protein modification by small protein conjugation or removal | 0.00238892 | 0.00083461 | 3 |
| BP | GO:0000302 | response to reactive oxygen species | 0.00239038 | 0.00083512 | 3 |
| BP | GO:0002639 | positive regulation of immunoglobulin production | 0.00240739 | 0.00084107 | 2 |
| BP | GO:0042149 | cellular response to glucose starvation | 0.00240739 | 0.00084107 | 2 |
| BP | GO:0071216 | cellular response to biotic stimulus | 0.00242618 | 0.00084763 | 3 |
| BP | GO:0048872 | homeostasis of number of cells | 0.00270999 | 0.00094679 | 3 |
| BP | GO:0071560 | cellular response to transforming growth factor beta stimulus | 0.00277647 | 0.00097002 | 3 |
| BP | GO:0045637 | regulation of myeloid cell differentiation | 0.00281064 | 0.00098195 | 3 |
| BP | GO:0045646 | regulation of erythrocyte differentiation | 0.0028192 | 0.00098494 | 2 |
| BP | GO:2001239 | regulation of extrinsic apoptotic signaling pathway in absence of ligand | 0.0028192 | 0.00098494 | 2 |
| BP | GO:0071559 | response to transforming growth factor beta | 0.00284708 | 0.00099468 | 3 |
| BP | GO:0001774 | microglial cell activation | 0.00284708 | 0.00099468 | 2 |
| BP | GO:0002269 | leukocyte activation involved in inflammatory response | 0.00284708 | 0.00099468 | 2 |
| BP | GO:0032757 | positive regulation of interleukin-8 production | 0.00292278 | 0.00102113 | 2 |
| BP | GO:0051091 | positive regulation of DNA-binding transcription factor activity | 0.00292278 | 0.00102113 | 3 |
| BP | GO:0140014 | mitotic nuclear division | 0.00293407 | 0.00102508 | 3 |
| BP | GO:0001961 | positive regulation of cytokine-mediated signaling pathway | 0.00293407 | 0.00102508 | 2 |
| BP | GO:0031113 | regulation of microtubule polymerization | 0.00293407 | 0.00102508 | 2 |
| BP | GO:0031334 | positive regulation of protein complex assembly | 0.00303264 | 0.00105951 | 3 |
| BP | GO:0031647 | regulation of protein stability | 0.00353923 | 0.0012365 | 3 |
| BP | GO:0065002 | intracellular protein transmembrane transport | 0.00353923 | 0.0012365 | 2 |
| BP | GO:0090307 | mitotic spindle assembly | 0.00353923 | 0.0012365 | 2 |
| BP | GO:0060760 | positive regulation of response to cytokine stimulus | 0.00363162 | 0.00126878 | 2 |
| BP | GO:0061900 | glial cell activation | 0.00372443 | 0.0013012 | 2 |
| BP | GO:0070372 | regulation of ERK1 and ERK2 cascade | 0.00398162 | 0.00139106 | 3 |
| BP | GO:0010803 | regulation of tumor necrosis factor-mediated signaling pathway | 0.0040427 | 0.0014124 | 2 |
| BP | GO:0002753 | cytoplasmic pattern recognition receptor signaling pathway | 0.00409964 | 0.00143229 | 2 |
| BP | GO:0070059 | intrinsic apoptotic signaling pathway in response to endoplasmic reticulum stress | 0.00409964 | 0.00143229 | 2 |
| BP | GO:0002637 | regulation of immunoglobulin production | 0.00419436 | 0.00146538 | 2 |
| BP | GO:0036498 | IRE1-mediated unfolded protein response | 0.00428943 | 0.0014986 | 2 |
| BP | GO:0002548 | monocyte chemotaxis | 0.00434603 | 0.00151837 | 2 |
| BP | GO:0071806 | protein transmembrane transport | 0.00434603 | 0.00151837 | 2 |
| BP | GO:0070371 | ERK1 and ERK2 cascade | 0.00434928 | 0.00151951 | 3 |
| BP | GO:0032496 | response to lipopolysaccharide | 0.00484787 | 0.0016937 | 3 |
| BP | GO:0038034 | signal transduction in absence of ligand | 0.00514771 | 0.00179845 | 2 |
| BP | GO:0097192 | extrinsic apoptotic signaling pathway in absence of ligand | 0.00514771 | 0.00179845 | 2 |
| BP | GO:0002237 | response to molecule of bacterial origin | 0.00528758 | 0.00184732 | 3 |
| BP | GO:0032677 | regulation of interleukin-8 production | 0.00530071 | 0.00185191 | 2 |
| BP | GO:0070373 | negative regulation of ERK1 and ERK2 cascade | 0.00530071 | 0.00185191 | 2 |
| BP | GO:0150076 | neuroinflammatory response | 0.00537898 | 0.00187925 | 2 |
| BP | GO:0007178 | transmembrane receptor protein serine/threonine kinase signaling pathway | 0.00537898 | 0.00187925 | 3 |
| BP | GO:0046785 | microtubule polymerization | 0.00545359 | 0.00190532 | 2 |
| BP | GO:0031397 | negative regulation of protein ubiquitination | 0.00555228 | 0.0019398 | 2 |
| BP | GO:0031016 | pancreas development | 0.00565119 | 0.00197435 | 2 |
| BP | GO:0031331 | positive regulation of cellular catabolic process | 0.00566046 | 0.00197759 | 3 |
| BP | GO:0031110 | regulation of microtubule polymerization or depolymerization | 0.00566046 | 0.00197759 | 2 |
| BP | GO:1900034 | regulation of cellular response to heat | 0.00566046 | 0.00197759 | 2 |
| BP | GO:0010038 | response to metal ion | 0.00574917 | 0.00200858 | 3 |
| BP | GO:0030512 | negative regulation of transforming growth factor beta receptor signaling pathway | 0.00581294 | 0.00203086 | 2 |
| BP | GO:0032436 | positive regulation of proteasomal ubiquitin-dependent protein catabolic process | 0.00581294 | 0.00203086 | 2 |
| BP | GO:0032637 | interleukin-8 production | 0.00591143 | 0.00206527 | 2 |
| BP | GO:0071277 | cellular response to calcium ion | 0.00596528 | 0.00208409 | 2 |
| BP | GO:1903845 | negative regulation of cellular response to transforming growth factor beta stimulus | 0.00596528 | 0.00208409 | 2 |
| BP | GO:1903321 | negative regulation of protein modification by small protein conjugation or removal | 0.00665106 | 0.00232368 | 2 |
| BP | GO:0071674 | mononuclear cell migration | 0.00690348 | 0.00241187 | 2 |
| BP | GO:0000280 | nuclear division | 0.00746582 | 0.00260833 | 3 |
| BP | GO:0002702 | positive regulation of production of molecular mediator of immune response | 0.00746582 | 0.00260833 | 2 |
| BP | GO:0042116 | macrophage activation | 0.00746582 | 0.00260833 | 2 |
| BP | GO:2000060 | positive regulation of ubiquitin-dependent protein catabolic process | 0.00746582 | 0.00260833 | 2 |
| BP | GO:0032755 | positive regulation of interleukin-6 production | 0.00756842 | 0.00264418 | 2 |
| BP | GO:0030099 | myeloid cell differentiation | 0.0076899 | 0.00268662 | 3 |
| BP | GO:2001243 | negative regulation of intrinsic apoptotic signaling pathway | 0.00774214 | 0.00270487 | 2 |
| BP | GO:0043161 | proteasome-mediated ubiquitin-dependent protein catabolic process | 0.00774214 | 0.00270487 | 3 |
| BP | GO:0009896 | positive regulation of catabolic process | 0.00790261 | 0.00276093 | 3 |
| BP | GO:0048661 | positive regulation of smooth muscle cell proliferation | 0.00806363 | 0.00281719 | 2 |
| BP | GO:0001933 | negative regulation of protein phosphorylation | 0.00806363 | 0.00281719 | 3 |
| BP | GO:0051052 | regulation of DNA metabolic process | 0.00806363 | 0.00281719 | 3 |
| BP | GO:1901800 | positive regulation of proteasomal protein catabolic process | 0.00833712 | 0.00291274 | 2 |
| BP | GO:2001237 | negative regulation of extrinsic apoptotic signaling pathway | 0.00833712 | 0.00291274 | 2 |
| BP | GO:0007052 | mitotic spindle organization | 0.00860016 | 0.00300464 | 2 |
| BP | GO:0051225 | spindle assembly | 0.00883702 | 0.00308739 | 2 |
| BP | GO:0009314 | response to radiation | 0.00883702 | 0.00308739 | 3 |
| BP | GO:0048285 | organelle fission | 0.00883702 | 0.00308739 | 3 |
| BP | GO:0031109 | microtubule polymerization or depolymerization | 0.00934228 | 0.00326391 | 2 |
| BP | GO:0002688 | regulation of leukocyte chemotaxis | 0.00955166 | 0.00333706 | 2 |
| BP | GO:0030218 | erythrocyte differentiation | 0.00955166 | 0.00333706 | 2 |
| BP | GO:0043254 | regulation of protein complex assembly | 0.00964273 | 0.00336888 | 3 |
| BP | GO:0042326 | negative regulation of phosphorylation | 0.00964273 | 0.00336888 | 3 |
| BP | GO:0010498 | proteasomal protein catabolic process | 0.01012129 | 0.00353607 | 3 |
| BP | GO:0017015 | regulation of transforming growth factor beta receptor signaling pathway | 0.01024385 | 0.00357889 | 2 |
| BP | GO:1903052 | positive regulation of proteolysis involved in cellular protein catabolic process | 0.01024385 | 0.00357889 | 2 |
| BP | GO:0046718 | viral entry into host cell | 0.01026669 | 0.00358687 | 2 |
| BP | GO:0043312 | neutrophil degranulation | 0.01026669 | 0.00358687 | 3 |
| BP | GO:0032434 | regulation of proteasomal ubiquitin-dependent protein catabolic process | 0.01026669 | 0.00358687 | 2 |
| BP | GO:0034101 | erythrocyte homeostasis | 0.01026669 | 0.00358687 | 2 |
| BP | GO:1903844 | regulation of cellular response to transforming growth factor beta stimulus | 0.01026669 | 0.00358687 | 2 |
| BP | GO:0002283 | neutrophil activation involved in immune response | 0.0102909 | 0.00359533 | 3 |
| BP | GO:0008637 | apoptotic mitochondrial changes | 0.01047596 | 0.00365998 | 2 |
| BP | GO:0090101 | negative regulation of transmembrane receptor protein serine/threonine kinase signaling pathway | 0.01071552 | 0.00374368 | 2 |
| BP | GO:0042119 | neutrophil activation | 0.01071552 | 0.00374368 | 3 |
| BP | GO:1902850 | microtubule cytoskeleton organization involved in mitosis | 0.01147019 | 0.00400734 | 2 |
| BP | GO:0045598 | regulation of fat cell differentiation | 0.01157603 | 0.00404431 | 2 |
| BP | GO:0030260 | entry into host cell | 0.01158907 | 0.00404887 | 2 |
| BP | GO:0032273 | positive regulation of protein polymerization | 0.01158907 | 0.00404887 | 2 |
| BP | GO:0044409 | entry into host | 0.01158907 | 0.00404887 | 2 |
| BP | GO:0051806 | entry into cell of other organism involved in symbiotic interaction | 0.01158907 | 0.00404887 | 2 |
| BP | GO:0051828 | entry into other organism involved in symbiotic interaction | 0.01158907 | 0.00404887 | 2 |
| BP | GO:0001889 | liver development | 0.01169428 | 0.00408563 | 2 |
| BP | GO:0061008 | hepaticobiliary system development | 0.01214346 | 0.00424256 | 2 |
| BP | GO:0002700 | regulation of production of molecular mediator of immune response | 0.01224917 | 0.00427949 | 2 |
| BP | GO:1903364 | positive regulation of cellular protein catabolic process | 0.01235488 | 0.00431642 | 2 |
| BP | GO:0050871 | positive regulation of B cell activation | 0.012635 | 0.00441428 | 2 |
| BP | GO:1902107 | positive regulation of leukocyte differentiation | 0.01291669 | 0.0045127 | 2 |
| BP | GO:0051384 | response to glucocorticoid | 0.01319992 | 0.00461165 | 2 |
| BP | GO:0071236 | cellular response to antibiotic | 0.01330623 | 0.00464879 | 2 |
| BP | GO:0051092 | positive regulation of NF-kappaB transcription factor activity | 0.01351885 | 0.00472308 | 2 |
| BP | GO:2000058 | regulation of ubiquitin-dependent protein catabolic process | 0.01351885 | 0.00472308 | 2 |
| BP | GO:0032675 | regulation of interleukin-6 production | 0.01398414 | 0.00488563 | 2 |
| BP | GO:0032635 | interleukin-6 production | 0.01557227 | 0.00544048 | 2 |
| BP | GO:0031960 | response to corticosteroid | 0.01567989 | 0.00547808 | 2 |
| BP | GO:2001242 | regulation of intrinsic apoptotic signaling pathway | 0.01616923 | 0.00564904 | 2 |
| BP | GO:0090288 | negative regulation of cellular response to growth factor stimulus | 0.01627696 | 0.00568668 | 2 |
| BP | GO:0033209 | tumor necrosis factor-mediated signaling pathway | 0.01638466 | 0.0057243 | 2 |
| BP | GO:0034614 | cellular response to reactive oxygen species | 0.01649231 | 0.00576191 | 2 |
| BP | GO:0048660 | regulation of smooth muscle cell proliferation | 0.01651566 | 0.00577007 | 2 |
| BP | GO:0051100 | negative regulation of binding | 0.01651566 | 0.00577007 | 2 |
| BP | GO:0007051 | spindle organization | 0.01662312 | 0.00580761 | 2 |
| BP | GO:0048659 | smooth muscle cell proliferation | 0.01673053 | 0.00584514 | 2 |
| BP | GO:0050821 | protein stabilization | 0.01800578 | 0.00629067 | 2 |
| BP | GO:0002285 | lymphocyte activation involved in immune response | 0.01851105 | 0.0064672 | 2 |
| BP | GO:0061136 | regulation of proteasomal protein catabolic process | 0.0188191 | 0.00657482 | 2 |
| BP | GO:0043409 | negative regulation of MAPK cascade | 0.01883399 | 0.00658002 | 2 |
| BP | GO:0050864 | regulation of B cell activation | 0.01883399 | 0.00658002 | 2 |
| BP | GO:0030308 | negative regulation of cell growth | 0.01904902 | 0.00665515 | 2 |
| BP | GO:0070507 | regulation of microtubule cytoskeleton organization | 0.01904902 | 0.00665515 | 2 |
| BP | GO:0071248 | cellular response to metal ion | 0.01976075 | 0.00690381 | 2 |
| BP | GO:0001935 | endothelial cell proliferation | 0.01986817 | 0.00694134 | 2 |
| BP | GO:0002377 | immunoglobulin production | 0.02017893 | 0.00704991 | 2 |
| BP | GO:1902115 | regulation of organelle assembly | 0.02028623 | 0.00708739 | 2 |
| BP | GO:0002685 | regulation of leukocyte migration | 0.02059783 | 0.00719626 | 2 |
| BP | GO:0002221 | pattern recognition receptor signaling pathway | 0.02070501 | 0.0072337 | 2 |
| BP | GO:0050870 | positive regulation of T cell activation | 0.02163894 | 0.00755999 | 2 |
| BP | GO:1902905 | positive regulation of supramolecular fiber organization | 0.02195494 | 0.00767039 | 2 |
| BP | GO:0071222 | cellular response to lipopolysaccharide | 0.02206181 | 0.00770773 | 2 |
| BP | GO:0050679 | positive regulation of epithelial cell proliferation | 0.02216862 | 0.00774504 | 2 |
| BP | GO:0051701 | interaction with host | 0.02221341 | 0.00776069 | 2 |
| BP | GO:0097529 | myeloid leukocyte migration | 0.02221341 | 0.00776069 | 2 |
| BP | GO:0045348 | positive regulation of MHC class II biosynthetic process | 0.02221341 | 0.00776069 | 1 |
| BP | GO:0045657 | positive regulation of monocyte differentiation | 0.02221341 | 0.00776069 | 1 |
| BP | GO:0070091 | glucagon secretion | 0.02221341 | 0.00776069 | 1 |
| BP | GO:0097050 | type B pancreatic cell apoptotic process | 0.02221341 | 0.00776069 | 1 |
| BP | GO:1903332 | regulation of protein folding | 0.02221341 | 0.00776069 | 1 |
| BP | GO:1903897 | regulation of PERK-mediated unfolded protein response | 0.02221341 | 0.00776069 | 1 |
| BP | GO:0071219 | cellular response to molecule of bacterial origin | 0.02250412 | 0.00786226 | 2 |
| BP | GO:0045732 | positive regulation of protein catabolic process | 0.02271671 | 0.00793653 | 2 |
| BP | GO:1903050 | regulation of proteolysis involved in cellular protein catabolic process | 0.02271671 | 0.00793653 | 2 |
| BP | GO:0002699 | positive regulation of immune effector process | 0.02294253 | 0.00801542 | 2 |
| BP | GO:0050920 | regulation of chemotaxis | 0.02294253 | 0.00801542 | 2 |
| BP | GO:0071241 | cellular response to inorganic substance | 0.02294253 | 0.00801542 | 2 |
| BP | GO:0032271 | regulation of protein polymerization | 0.02294253 | 0.00801542 | 2 |
| BP | GO:0032886 | regulation of microtubule-based process | 0.02294253 | 0.00801542 | 2 |
| BP | GO:1903039 | positive regulation of leukocyte cell-cell adhesion | 0.02294253 | 0.00801542 | 2 |
| BP | GO:0006620 | posttranslational protein targeting to endoplasmic reticulum membrane | 0.02299187 | 0.00803266 | 1 |
| BP | GO:0032494 | response to peptidoglycan | 0.02299187 | 0.00803266 | 1 |
| BP | GO:0036462 | TRAIL-activated apoptotic signaling pathway | 0.02299187 | 0.00803266 | 1 |
| BP | GO:1900102 | negative regulation of endoplasmic reticulum unfolded protein response | 0.02299187 | 0.00803266 | 1 |
| BP | GO:2000551 | regulation of T-helper 2 cell cytokine production | 0.02299187 | 0.00803266 | 1 |
| BP | GO:0045444 | fat cell differentiation | 0.0233733 | 0.00816592 | 2 |
| BP | GO:0030595 | leukocyte chemotaxis | 0.02347889 | 0.00820281 | 2 |
| BP | GO:0051495 | positive regulation of cytoskeleton organization | 0.02378819 | 0.00831087 | 2 |
| BP | GO:0051054 | positive regulation of DNA metabolic process | 0.02396822 | 0.00837377 | 2 |
| BP | GO:0006983 | ER overload response | 0.02396822 | 0.00837377 | 1 |
| BP | GO:0035437 | maintenance of protein localization in endoplasmic reticulum | 0.02396822 | 0.00837377 | 1 |
| BP | GO:0043922 | negative regulation by host of viral transcription | 0.02396822 | 0.00837377 | 1 |
| BP | GO:0045579 | positive regulation of B cell differentiation | 0.02396822 | 0.00837377 | 1 |
| BP | GO:0072540 | T-helper 17 cell lineage commitment | 0.02396822 | 0.00837377 | 1 |
| BP | GO:1902510 | regulation of apoptotic DNA fragmentation | 0.02396822 | 0.00837377 | 1 |
| BP | GO:1903894 | regulation of IRE1-mediated unfolded protein response | 0.02396822 | 0.00837377 | 1 |
| BP | GO:0033002 | muscle cell proliferation | 0.02495703 | 0.00871923 | 2 |
| BP | GO:0051402 | neuron apoptotic process | 0.02495703 | 0.00871923 | 2 |
| BP | GO:0030217 | T cell differentiation | 0.02495703 | 0.00871923 | 2 |
| BP | GO:0031953 | negative regulation of protein autophosphorylation | 0.02495703 | 0.00871923 | 1 |
| BP | GO:0035745 | T-helper 2 cell cytokine production | 0.02495703 | 0.00871923 | 1 |
| BP | GO:0045779 | negative regulation of bone resorption | 0.02495703 | 0.00871923 | 1 |
| BP | GO:0061029 | eyelid development in camera-type eye | 0.02495703 | 0.00871923 | 1 |
| BP | GO:0061042 | vascular wound healing | 0.02495703 | 0.00871923 | 1 |
| BP | GO:0071287 | cellular response to manganese ion | 0.02495703 | 0.00871923 | 1 |
| BP | GO:1900103 | positive regulation of endoplasmic reticulum unfolded protein response | 0.02495703 | 0.00871923 | 1 |
| BP | GO:0090092 | regulation of transmembrane receptor protein serine/threonine kinase signaling pathway | 0.0249735 | 0.00872498 | 2 |
| BP | GO:1903362 | regulation of cellular protein catabolic process | 0.02587143 | 0.00903869 | 2 |
| BP | GO:0045926 | negative regulation of growth | 0.02587143 | 0.00903869 | 2 |
| BP | GO:0045346 | regulation of MHC class II biosynthetic process | 0.02587143 | 0.00903869 | 1 |
| BP | GO:0090231 | regulation of spindle checkpoint | 0.02587143 | 0.00903869 | 1 |
| BP | GO:0090266 | regulation of mitotic cell cycle spindle assembly checkpoint | 0.02587143 | 0.00903869 | 1 |
| BP | GO:1902043 | positive regulation of extrinsic apoptotic signaling pathway via death domain receptors | 0.02587143 | 0.00903869 | 1 |
| BP | GO:1903504 | regulation of mitotic spindle checkpoint | 0.02587143 | 0.00903869 | 1 |
| BP | GO:1903624 | regulation of DNA catabolic process | 0.02587143 | 0.00903869 | 1 |
| BP | GO:2000345 | regulation of hepatocyte proliferation | 0.02587143 | 0.00903869 | 1 |
| BP | GO:0022409 | positive regulation of cell-cell adhesion | 0.02682228 | 0.00937089 | 2 |
| BP | GO:0006839 | mitochondrial transport | 0.02692602 | 0.00940713 | 2 |
| BP | GO:0045073 | regulation of chemokine biosynthetic process | 0.02700985 | 0.00943642 | 1 |
| BP | GO:0045342 | MHC class II biosynthetic process | 0.02700985 | 0.00943642 | 1 |
| BP | GO:0046851 | negative regulation of bone remodeling | 0.02700985 | 0.00943642 | 1 |
| BP | GO:0055089 | fatty acid homeostasis | 0.02700985 | 0.00943642 | 1 |
| BP | GO:1900119 | positive regulation of execution phase of apoptosis | 0.02700985 | 0.00943642 | 1 |
| BP | GO:0001780 | neutrophil homeostasis | 0.02787089 | 0.00973724 | 1 |
| BP | GO:0002070 | epithelial cell maturation | 0.02787089 | 0.00973724 | 1 |
| BP | GO:0002295 | T-helper cell lineage commitment | 0.02787089 | 0.00973724 | 1 |
| BP | GO:0002830 | positive regulation of type 2 immune response | 0.02787089 | 0.00973724 | 1 |
| BP | GO:0034975 | protein folding in endoplasmic reticulum | 0.02787089 | 0.00973724 | 1 |
| BP | GO:0035970 | peptidyl-threonine dephosphorylation | 0.02787089 | 0.00973724 | 1 |
| BP | GO:0042033 | chemokine biosynthetic process | 0.02787089 | 0.00973724 | 1 |
| BP | GO:0050755 | chemokine metabolic process | 0.02787089 | 0.00973724 | 1 |
| BP | GO:0051447 | negative regulation of meiotic cell cycle | 0.02787089 | 0.00973724 | 1 |
| BP | GO:0050714 | positive regulation of protein secretion | 0.02787089 | 0.00973724 | 2 |
| BP | GO:1902105 | regulation of leukocyte differentiation | 0.02857789 | 0.00998425 | 2 |
| BP | GO:0010715 | regulation of extracellular matrix disassembly | 0.0286258 | 0.01000099 | 1 |
| BP | GO:0032740 | positive regulation of interleukin-17 production | 0.0286258 | 0.01000099 | 1 |
| BP | GO:0032966 | negative regulation of collagen biosynthetic process | 0.0286258 | 0.01000099 | 1 |
| BP | GO:0043373 | CD4-positive, alpha-beta T cell lineage commitment | 0.0286258 | 0.01000099 | 1 |
| BP | GO:0043923 | positive regulation by host of viral transcription | 0.0286258 | 0.01000099 | 1 |
| BP | GO:0048532 | anatomical structure arrangement | 0.0286258 | 0.01000099 | 1 |
| BP | GO:0071850 | mitotic cell cycle arrest | 0.0286258 | 0.01000099 | 1 |
| BP | GO:0097501 | stress response to metal ion | 0.0286258 | 0.01000099 | 1 |
| BP | GO:0030522 | intracellular receptor signaling pathway | 0.02929886 | 0.01023613 | 2 |
| BP | GO:0051258 | protein polymerization | 0.02950231 | 0.01030721 | 2 |
| BP | GO:0010713 | negative regulation of collagen metabolic process | 0.02950231 | 0.01030721 | 1 |
| BP | GO:0010832 | negative regulation of myotube differentiation | 0.02950231 | 0.01030721 | 1 |
| BP | GO:0035994 | response to muscle stretch | 0.02950231 | 0.01030721 | 1 |
| BP | GO:0072574 | hepatocyte proliferation | 0.02950231 | 0.01030721 | 1 |
| BP | GO:0072575 | epithelial cell proliferation involved in liver morphogenesis | 0.02950231 | 0.01030721 | 1 |
| BP | GO:0150078 | positive regulation of neuroinflammatory response | 0.02950231 | 0.01030721 | 1 |
| BP | GO:0002440 | production of molecular mediator of immune response | 0.02957226 | 0.01033165 | 2 |
| BP | GO:0002793 | positive regulation of peptide secretion | 0.02957226 | 0.01033165 | 2 |
| BP | GO:0097193 | intrinsic apoptotic signaling pathway | 0.02957226 | 0.01033165 | 2 |
| BP | GO:0071356 | cellular response to tumor necrosis factor | 0.02957226 | 0.01033165 | 2 |
| BP | GO:0090287 | regulation of cellular response to growth factor stimulus | 0.02957226 | 0.01033165 | 2 |
| BP | GO:0002313 | mature B cell differentiation involved in immune response | 0.02957226 | 0.01033165 | 1 |
| BP | GO:0002363 | alpha-beta T cell lineage commitment | 0.02957226 | 0.01033165 | 1 |
| BP | GO:0002689 | negative regulation of leukocyte chemotaxis | 0.02957226 | 0.01033165 | 1 |
| BP | GO:0002726 | positive regulation of T cell cytokine production | 0.02957226 | 0.01033165 | 1 |
| BP | GO:0010888 | negative regulation of lipid storage | 0.02957226 | 0.01033165 | 1 |
| BP | GO:0030150 | protein import into mitochondrial matrix | 0.02957226 | 0.01033165 | 1 |
| BP | GO:0051131 | chaperone-mediated protein complex assembly | 0.02957226 | 0.01033165 | 1 |
| BP | GO:0060252 | positive regulation of glial cell proliferation | 0.02957226 | 0.01033165 | 1 |
| BP | GO:0071243 | cellular response to arsenic-containing substance | 0.02957226 | 0.01033165 | 1 |
| BP | GO:0072576 | liver morphogenesis | 0.02957226 | 0.01033165 | 1 |
| BP | GO:1902074 | response to salt | 0.02957226 | 0.01033165 | 1 |
| BP | GO:0048511 | rhythmic process | 0.02995961 | 0.01046698 | 2 |
| BP | GO:0002758 | innate immune response-activating signal transduction | 0.03035982 | 0.0106068 | 2 |
| BP | GO:0010042 | response to manganese ion | 0.03035982 | 0.0106068 | 1 |
| BP | GO:0034104 | negative regulation of tissue remodeling | 0.03035982 | 0.0106068 | 1 |
| BP | GO:0043369 | CD4-positive or CD8-positive, alpha-beta T cell lineage commitment | 0.03035982 | 0.0106068 | 1 |
| BP | GO:0045655 | regulation of monocyte differentiation | 0.03035982 | 0.0106068 | 1 |
| BP | GO:0070262 | peptidyl-serine dephosphorylation | 0.03035982 | 0.0106068 | 1 |
| BP | GO:1903978 | regulation of microglial cell activation | 0.03035982 | 0.0106068 | 1 |
| BP | GO:0060326 | cell chemotaxis | 0.03086875 | 0.01078461 | 2 |
| BP | GO:1903037 | regulation of leukocyte cell-cell adhesion | 0.03086875 | 0.01078461 | 2 |
| BP | GO:0046034 | ATP metabolic process | 0.03096949 | 0.0108198 | 2 |
| BP | GO:0032682 | negative regulation of chemokine production | 0.0312959 | 0.01093384 | 1 |
| BP | GO:0035743 | CD4-positive, alpha-beta T cell cytokine production | 0.0312959 | 0.01093384 | 1 |
| BP | GO:1903055 | positive regulation of extracellular matrix organization | 0.0312959 | 0.01093384 | 1 |
| BP | GO:0042113 | B cell activation | 0.03156655 | 0.01102839 | 2 |
| BP | GO:0034612 | response to tumor necrosis factor | 0.03186215 | 0.01113167 | 2 |
| BP | GO:0050863 | regulation of T cell activation | 0.03210344 | 0.01121597 | 2 |
| BP | GO:0006925 | inflammatory cell apoptotic process | 0.03210344 | 0.01121597 | 1 |
| BP | GO:0032069 | regulation of nuclease activity | 0.03210344 | 0.01121597 | 1 |
| BP | GO:0043496 | regulation of protein homodimerization activity | 0.03210344 | 0.01121597 | 1 |
| BP | GO:1901522 | positive regulation of transcription from RNA polymerase II promoter involved in cellular response to chemical stimulus | 0.03210344 | 0.01121597 | 1 |
| BP | GO:0002218 | activation of innate immune response | 0.03265869 | 0.01140996 | 2 |
| BP | GO:0060055 | angiogenesis involved in wound healing | 0.03335837 | 0.0116544 | 1 |
| BP | GO:0019058 | viral life cycle | 0.03424086 | 0.01196272 | 2 |
| BP | GO:0051235 | maintenance of location | 0.03449774 | 0.01205247 | 2 |
| BP | GO:0032897 | negative regulation of viral transcription | 0.03449774 | 0.01205247 | 1 |
| BP | GO:0051251 | positive regulation of lymphocyte activation | 0.03513876 | 0.01227642 | 2 |
| BP | GO:0009205 | purine ribonucleoside triphosphate metabolic process | 0.03523785 | 0.01231103 | 2 |
| BP | GO:0007159 | leukocyte cell-cell adhesion | 0.03531237 | 0.01233707 | 2 |
| BP | GO:0002335 | mature B cell differentiation | 0.03531237 | 0.01233707 | 1 |
| BP | GO:0021680 | cerebellar Purkinje cell layer development | 0.03531237 | 0.01233707 | 1 |
| BP | GO:0090025 | regulation of monocyte chemotaxis | 0.03531237 | 0.01233707 | 1 |
| BP | GO:0009167 | purine ribonucleoside monophosphate metabolic process | 0.03572993 | 0.01248295 | 2 |
| BP | GO:0009126 | purine nucleoside monophosphate metabolic process | 0.03572993 | 0.01248295 | 2 |
| BP | GO:0009199 | ribonucleoside triphosphate metabolic process | 0.03572993 | 0.01248295 | 2 |
| BP | GO:0009144 | purine nucleoside triphosphate metabolic process | 0.03582861 | 0.01251743 | 2 |
| BP | GO:0001101 | response to acid chemical | 0.03589784 | 0.01254162 | 2 |
| BP | GO:0002360 | T cell lineage commitment | 0.03589784 | 0.01254162 | 1 |
| BP | GO:0006309 | apoptotic DNA fragmentation | 0.03589784 | 0.01254162 | 1 |
| BP | GO:0072539 | T-helper 17 cell differentiation | 0.03589784 | 0.01254162 | 1 |
| BP | GO:0070997 | neuron death | 0.03652066 | 0.01275921 | 2 |
| BP | GO:0000188 | inactivation of MAPK activity | 0.0366569 | 0.01280681 | 1 |
| BP | GO:0043921 | modulation by host of viral transcription | 0.0366569 | 0.01280681 | 1 |
| BP | GO:0048143 | astrocyte activation | 0.0366569 | 0.01280681 | 1 |
| BP | GO:0052472 | modulation by host of symbiont transcription | 0.0366569 | 0.01280681 | 1 |
| BP | GO:2000144 | positive regulation of DNA-templated transcription, initiation | 0.0366569 | 0.01280681 | 1 |
| BP | GO:1902903 | regulation of supramolecular fiber organization | 0.03671167 | 0.01282594 | 2 |
| BP | GO:0030098 | lymphocyte differentiation | 0.03680972 | 0.0128602 | 2 |
| BP | GO:0009161 | ribonucleoside monophosphate metabolic process | 0.03690771 | 0.01289443 | 2 |
| BP | GO:0045577 | regulation of B cell differentiation | 0.03749182 | 0.0130985 | 1 |
| BP | GO:0052312 | modulation of transcription in other organism involved in symbiotic interaction | 0.03749182 | 0.0130985 | 1 |
| BP | GO:0009141 | nucleoside triphosphate metabolic process | 0.03819749 | 0.01334505 | 2 |
| BP | GO:0045862 | positive regulation of proteolysis | 0.03820197 | 0.01334661 | 2 |
| BP | GO:0006402 | mRNA catabolic process | 0.03820197 | 0.01334661 | 2 |
| BP | GO:0010575 | positive regulation of vascular endothelial growth factor production | 0.03820197 | 0.01334661 | 1 |
| BP | GO:0070102 | interleukin-6-mediated signaling pathway | 0.03820197 | 0.01334661 | 1 |
| BP | GO:0072538 | T-helper 17 type immune response | 0.03820197 | 0.01334661 | 1 |
| BP | GO:0035690 | cellular response to drug | 0.03898128 | 0.01361888 | 2 |
| BP | GO:0002828 | regulation of type 2 immune response | 0.03919703 | 0.01369425 | 1 |
| BP | GO:0071480 | cellular response to gamma radiation | 0.03919703 | 0.01369425 | 1 |
| BP | GO:0051098 | regulation of binding | 0.03947248 | 0.01379049 | 2 |
| BP | GO:0009123 | nucleoside monophosphate metabolic process | 0.03977001 | 0.01389443 | 2 |
| BP | GO:0002675 | positive regulation of acute inflammatory response | 0.03985207 | 0.0139231 | 1 |
| BP | GO:0002724 | regulation of T cell cytokine production | 0.03985207 | 0.0139231 | 1 |
| BP | GO:0048710 | regulation of astrocyte differentiation | 0.03985207 | 0.0139231 | 1 |
| BP | GO:1902895 | positive regulation of pri-miRNA transcription by RNA polymerase II | 0.03985207 | 0.0139231 | 1 |
| BP | GO:0050678 | regulation of epithelial cell proliferation | 0.03985207 | 0.0139231 | 2 |
| BP | GO:0002696 | positive regulation of leukocyte activation | 0.04014128 | 0.01402415 | 2 |
| BP | GO:0042176 | regulation of protein catabolic process | 0.04014128 | 0.01402415 | 2 |
| BP | GO:0045089 | positive regulation of innate immune response | 0.04014128 | 0.01402415 | 2 |
| BP | GO:0045765 | regulation of angiogenesis | 0.04030133 | 0.01408006 | 2 |
| BP | GO:0046685 | response to arsenic-containing substance | 0.04030133 | 0.01408006 | 1 |
| BP | GO:1901976 | regulation of cell cycle checkpoint | 0.04030133 | 0.01408006 | 1 |
| BP | GO:2000778 | positive regulation of interleukin-6 secretion | 0.04030133 | 0.01408006 | 1 |
| BP | GO:0048545 | response to steroid hormone | 0.04042323 | 0.01412265 | 2 |
| BP | GO:0033028 | myeloid cell apoptotic process | 0.04132733 | 0.01443852 | 1 |
| BP | GO:0050867 | positive regulation of cell activation | 0.04132733 | 0.01443852 | 2 |
| BP | GO:0010639 | negative regulation of organelle organization | 0.04132733 | 0.01443852 | 2 |
| BP | GO:0000737 | DNA catabolic process, endonucleolytic | 0.04132733 | 0.01443852 | 1 |
| BP | GO:0007094 | mitotic spindle assembly checkpoint | 0.04132733 | 0.01443852 | 1 |
| BP | GO:0010574 | regulation of vascular endothelial growth factor production | 0.04132733 | 0.01443852 | 1 |
| BP | GO:0031577 | spindle checkpoint | 0.04132733 | 0.01443852 | 1 |
| BP | GO:0032660 | regulation of interleukin-17 production | 0.04132733 | 0.01443852 | 1 |
| BP | GO:0033120 | positive regulation of RNA splicing | 0.04132733 | 0.01443852 | 1 |
| BP | GO:0043368 | positive T cell selection | 0.04132733 | 0.01443852 | 1 |
| BP | GO:0070050 | neuron cellular homeostasis | 0.04132733 | 0.01443852 | 1 |
| BP | GO:0071173 | spindle assembly checkpoint | 0.04132733 | 0.01443852 | 1 |
| BP | GO:0071174 | mitotic spindle checkpoint | 0.04132733 | 0.01443852 | 1 |
| BP | GO:0006401 | RNA catabolic process | 0.0413685 | 0.0144529 | 2 |
| BP | GO:1903532 | positive regulation of secretion by cell | 0.04166087 | 0.01455504 | 2 |
| BP | GO:0030262 | apoptotic nuclear changes | 0.0419401 | 0.0146526 | 1 |
| BP | GO:0042092 | type 2 immune response | 0.0419401 | 0.0146526 | 1 |
| BP | GO:0060251 | regulation of glial cell proliferation | 0.0419401 | 0.0146526 | 1 |
| BP | GO:0022407 | regulation of cell-cell adhesion | 0.0419401 | 0.0146526 | 2 |
| BP | GO:0045785 | positive regulation of cell adhesion | 0.0419401 | 0.0146526 | 2 |
| BP | GO:0010573 | vascular endothelial growth factor production | 0.04248467 | 0.01484286 | 1 |
| BP | GO:0030224 | monocyte differentiation | 0.04248467 | 0.01484286 | 1 |
| BP | GO:0045740 | positive regulation of DNA replication | 0.04248467 | 0.01484286 | 1 |
| BP | GO:0045841 | negative regulation of mitotic metaphase/anaphase transition | 0.04248467 | 0.01484286 | 1 |
| BP | GO:1903131 | mononuclear cell differentiation | 0.04248467 | 0.01484286 | 1 |
| BP | GO:0007517 | muscle organ development | 0.04270579 | 0.01492011 | 2 |
| BP | GO:0006921 | cellular component disassembly involved in execution phase of apoptosis | 0.04293697 | 0.01500088 | 1 |
| BP | GO:0007431 | salivary gland development | 0.04293697 | 0.01500088 | 1 |
| BP | GO:0021587 | cerebellum morphogenesis | 0.04293697 | 0.01500088 | 1 |
| BP | GO:0071276 | cellular response to cadmium ion | 0.04293697 | 0.01500088 | 1 |
| BP | GO:1902100 | negative regulation of metaphase/anaphase transition of cell cycle | 0.04293697 | 0.01500088 | 1 |
| BP | GO:2000142 | regulation of DNA-templated transcription, initiation | 0.04293697 | 0.01500088 | 1 |
| BP | GO:0001558 | regulation of cell growth | 0.04317147 | 0.0150828 | 2 |
| BP | GO:0051222 | positive regulation of protein transport | 0.04346136 | 0.01518408 | 2 |
| BP | GO:0032620 | interleukin-17 production | 0.0434745 | 0.01518867 | 1 |
| BP | GO:1904037 | positive regulation of epithelial cell apoptotic process | 0.0434745 | 0.01518867 | 1 |
| BP | GO:1905898 | positive regulation of response to endoplasmic reticulum stress | 0.0434745 | 0.01518867 | 1 |
| BP | GO:2000279 | negative regulation of DNA biosynthetic process | 0.0434745 | 0.01518867 | 1 |
| BP | GO:1901342 | regulation of vasculature development | 0.04373653 | 0.01528022 | 2 |
| BP | GO:0002251 | organ or tissue specific immune response | 0.04379678 | 0.01530127 | 1 |
| BP | GO:0033574 | response to testosterone | 0.04379678 | 0.01530127 | 1 |
| BP | GO:0055090 | acylglycerol homeostasis | 0.04379678 | 0.01530127 | 1 |
| BP | GO:0070328 | triglyceride homeostasis | 0.04379678 | 0.01530127 | 1 |
| BP | GO:0072595 | maintenance of protein localization in organelle | 0.04379678 | 0.01530127 | 1 |
| BP | GO:1901998 | toxin transport | 0.04379678 | 0.01530127 | 1 |
| BP | GO:2000816 | negative regulation of mitotic sister chromatid separation | 0.04379678 | 0.01530127 | 1 |
| BP | GO:0051047 | positive regulation of secretion | 0.04409864 | 0.01540673 | 2 |
| BP | GO:0002369 | T cell cytokine production | 0.04437325 | 0.01550267 | 1 |
| BP | GO:0042036 | negative regulation of cytokine biosynthetic process | 0.04437325 | 0.01550267 | 1 |
| BP | GO:0045124 | regulation of bone resorption | 0.04437325 | 0.01550267 | 1 |
| BP | GO:1905819 | negative regulation of chromosome separation | 0.04437325 | 0.01550267 | 1 |
| BP | GO:0006605 | protein targeting | 0.04437325 | 0.01550267 | 2 |
| BP | GO:0048732 | gland development | 0.04440585 | 0.01551406 | 2 |
| BP | GO:0050673 | epithelial cell proliferation | 0.04440585 | 0.01551406 | 2 |
| BP | GO:0006308 | DNA catabolic process | 0.04440585 | 0.01551406 | 1 |
| BP | GO:0021575 | hindbrain morphogenesis | 0.04440585 | 0.01551406 | 1 |
| BP | GO:0044743 | protein transmembrane import into intracellular organelle | 0.04440585 | 0.01551406 | 1 |
| BP | GO:0050434 | positive regulation of viral transcription | 0.04440585 | 0.01551406 | 1 |
| BP | GO:0150077 | regulation of neuroinflammatory response | 0.04440585 | 0.01551406 | 1 |
| BP | GO:1900117 | regulation of execution phase of apoptosis | 0.04440585 | 0.01551406 | 1 |
| BP | GO:1902893 | regulation of pri-miRNA transcription by RNA polymerase II | 0.04440585 | 0.01551406 | 1 |
| BP | GO:1903053 | regulation of extracellular matrix organization | 0.04440585 | 0.01551406 | 1 |
| BP | GO:0033048 | negative regulation of mitotic sister chromatid segregation | 0.04527938 | 0.01581924 | 1 |
| BP | GO:0051154 | negative regulation of striated muscle cell differentiation | 0.04527938 | 0.01581924 | 1 |
| BP | GO:0021762 | substantia nigra development | 0.04604471 | 0.01608662 | 1 |
| BP | GO:0034198 | cellular response to amino acid starvation | 0.04604471 | 0.01608662 | 1 |
| BP | GO:0060612 | adipose tissue development | 0.04604471 | 0.01608662 | 1 |
| BP | GO:0045088 | regulation of innate immune response | 0.04659877 | 0.0162802 | 2 |
| BP | GO:0014002 | astrocyte development | 0.04659877 | 0.0162802 | 1 |
| BP | GO:0032965 | regulation of collagen biosynthetic process | 0.04659877 | 0.0162802 | 1 |
| BP | GO:0033046 | negative regulation of sister chromatid segregation | 0.04659877 | 0.0162802 | 1 |
| BP | GO:1901028 | regulation of mitochondrial outer membrane permeabilization involved in apoptotic signaling pathway | 0.04659877 | 0.0162802 | 1 |
| BP | GO:1904951 | positive regulation of establishment of protein localization | 0.04689991 | 0.01638541 | 2 |
| BP | GO:0002697 | regulation of immune effector process | 0.04718456 | 0.01648485 | 2 |
| BP | GO:0031018 | endocrine pancreas development | 0.04724087 | 0.01650453 | 1 |
| BP | GO:0051985 | negative regulation of chromosome segregation | 0.04724087 | 0.01650453 | 1 |
| BP | GO:0042110 | T cell activation | 0.04757055 | 0.01661971 | 2 |
| BP | GO:0010883 | regulation of lipid storage | 0.04757055 | 0.01661971 | 1 |
| BP | GO:0022602 | ovulation cycle process | 0.04757055 | 0.01661971 | 1 |
| BP | GO:0031648 | protein destabilization | 0.04757055 | 0.01661971 | 1 |
| BP | GO:0071354 | cellular response to interleukin-6 | 0.04757055 | 0.01661971 | 1 |
| BP | GO:0071675 | regulation of mononuclear cell migration | 0.04757055 | 0.01661971 | 1 |
| BP | GO:1990928 | response to amino acid starvation | 0.04757055 | 0.01661971 | 1 |
| BP | GO:0006953 | acute-phase response | 0.04798936 | 0.01676603 | 1 |
| BP | GO:0021695 | cerebellar cortex development | 0.04798936 | 0.01676603 | 1 |
| BP | GO:0031952 | regulation of protein autophosphorylation | 0.04798936 | 0.01676603 | 1 |
| BP | GO:0045058 | T cell selection | 0.04798936 | 0.01676603 | 1 |
| BP | GO:0061614 | pri-miRNA transcription by RNA polymerase II | 0.04798936 | 0.01676603 | 1 |
| BP | GO:1900744 | regulation of p38MAPK cascade | 0.04798936 | 0.01676603 | 1 |
| BP | GO:0050708 | regulation of protein secretion | 0.04826606 | 0.0168627 | 2 |
| BP | GO:0050769 | positive regulation of neurogenesis | 0.04829864 | 0.01687408 | 2 |
| BP | GO:0002711 | positive regulation of T cell mediated immunity | 0.04829864 | 0.01687408 | 1 |
| BP | GO:0007595 | lactation | 0.04829864 | 0.01687408 | 1 |
| BP | GO:0035272 | exocrine system development | 0.04829864 | 0.01687408 | 1 |
| BP | GO:0046850 | regulation of bone remodeling | 0.04829864 | 0.01687408 | 1 |
| BP | GO:0072604 | interleukin-6 secretion | 0.04829864 | 0.01687408 | 1 |
| BP | GO:0002686 | negative regulation of leukocyte migration | 0.04869672 | 0.01701316 | 1 |
| BP | GO:0010712 | regulation of collagen metabolic process | 0.04869672 | 0.01701316 | 1 |
| BP | GO:0051445 | regulation of meiotic cell cycle | 0.04869672 | 0.01701316 | 1 |
| BP | GO:1904707 | positive regulation of vascular smooth muscle cell proliferation | 0.04869672 | 0.01701316 | 1 |
| BP | GO:1990090 | cellular response to nerve growth factor stimulus | 0.04869672 | 0.01701316 | 1 |
| BP | GO:2001238 | positive regulation of extrinsic apoptotic signaling pathway | 0.04869672 | 0.01701316 | 1 |
| BP | GO:0016049 | cell growth | 0.04898722 | 0.01711465 | 2 |
| BP | GO:0051249 | regulation of lymphocyte activation | 0.04898722 | 0.01711465 | 2 |
| BP | GO:0010718 | positive regulation of epithelial to mesenchymal transition | 0.04898722 | 0.01711465 | 1 |
| BP | GO:0014009 | glial cell proliferation | 0.04898722 | 0.01711465 | 1 |
| BP | GO:0032873 | negative regulation of stress-activated MAPK cascade | 0.04898722 | 0.01711465 | 1 |
| BP | GO:0070303 | negative regulation of stress-activated protein kinase signaling cascade | 0.04898722 | 0.01711465 | 1 |
| BP | GO:0070741 | response to interleukin-6 | 0.04898722 | 0.01711465 | 1 |
| BP | GO:0030071 | regulation of mitotic metaphase/anaphase transition | 0.04956083 | 0.01731505 | 1 |
| BP | GO:0031103 | axon regeneration | 0.04956083 | 0.01731505 | 1 |
| BP | GO:0045599 | negative regulation of fat cell differentiation | 0.04956083 | 0.01731505 | 1 |
| BP | GO:0048146 | positive regulation of fibroblast proliferation | 0.04956083 | 0.01731505 | 1 |
| BP | GO:0045839 | negative regulation of mitotic nuclear division | 0.05032195 | 0.01758096 | 1 |
| BP | GO:1990089 | response to nerve growth factor | 0.05032195 | 0.01758096 | 1 |
| BP | GO:0050900 | leukocyte migration | 0.05067994 | 0.01770603 | 2 |
| BP | GO:0002791 | regulation of peptide secretion | 0.05067994 | 0.01770603 | 2 |
| BP | GO:0032964 | collagen biosynthetic process | 0.05067994 | 0.01770603 | 1 |
| BP | GO:0038066 | p38MAPK cascade | 0.05067994 | 0.01770603 | 1 |
| BP | GO:0071320 | cellular response to cAMP | 0.05067994 | 0.01770603 | 1 |
| BP | GO:1902099 | regulation of metaphase/anaphase transition of cell cycle | 0.05067994 | 0.01770603 | 1 |
| BP | GO:0002720 | positive regulation of cytokine production involved in immune response | 0.05112736 | 0.01786235 | 1 |
| BP | GO:0002763 | positive regulation of myeloid leukocyte differentiation | 0.05112736 | 0.01786235 | 1 |
| BP | GO:0007091 | metaphase/anaphase transition of mitotic cell cycle | 0.05112736 | 0.01786235 | 1 |
| BP | GO:0043392 | negative regulation of DNA binding | 0.05112736 | 0.01786235 | 1 |
| BP | GO:0097345 | mitochondrial outer membrane permeabilization | 0.05112736 | 0.01786235 | 1 |
| BP | GO:0010823 | negative regulation of mitochondrion organization | 0.05166423 | 0.01804991 | 1 |
| BP | GO:0042220 | response to cocaine | 0.05166423 | 0.01804991 | 1 |
| BP | GO:0043030 | regulation of macrophage activation | 0.05166423 | 0.01804991 | 1 |
| BP | GO:2000242 | negative regulation of reproductive process | 0.05166423 | 0.01804991 | 1 |
| BP | GO:0010332 | response to gamma radiation | 0.05239096 | 0.01830381 | 1 |
| BP | GO:0044784 | metaphase/anaphase transition of cell cycle | 0.05239096 | 0.01830381 | 1 |
| BP | GO:0010965 | regulation of mitotic sister chromatid separation | 0.05311175 | 0.01855563 | 1 |
| BP | GO:0043525 | positive regulation of neuron apoptotic process | 0.05311175 | 0.01855563 | 1 |
| BP | GO:0032722 | positive regulation of chemokine production | 0.05362429 | 0.0187347 | 1 |
| BP | GO:0042093 | T-helper cell differentiation | 0.05362429 | 0.0187347 | 1 |
| BP | GO:0055081 | anion homeostasis | 0.05362429 | 0.0187347 | 1 |
| BP | GO:1902041 | regulation of extrinsic apoptotic signaling pathway via death domain receptors | 0.05362429 | 0.0187347 | 1 |
| BP | GO:0001836 | release of cytochrome c from mitochondria | 0.05433148 | 0.01898177 | 1 |
| BP | GO:0031102 | neuron projection regeneration | 0.05433148 | 0.01898177 | 1 |
| BP | GO:0002294 | CD4-positive, alpha-beta T cell differentiation involved in immune response | 0.05482837 | 0.01915537 | 1 |
| BP | GO:0051306 | mitotic sister chromatid separation | 0.05482837 | 0.01915537 | 1 |
| BP | GO:0051784 | negative regulation of nuclear division | 0.05482837 | 0.01915537 | 1 |
| BP | GO:1902110 | positive regulation of mitochondrial membrane permeability involved in apoptotic process | 0.05482837 | 0.01915537 | 1 |
| BP | GO:0002287 | alpha-beta T cell activation involved in immune response | 0.05531749 | 0.01932625 | 1 |
| BP | GO:0002293 | alpha-beta T cell differentiation involved in immune response | 0.05531749 | 0.01932625 | 1 |
| BP | GO:0090303 | positive regulation of wound healing | 0.05531749 | 0.01932625 | 1 |
| BP | GO:1905953 | negative regulation of lipid localization | 0.05531749 | 0.01932625 | 1 |
| BP | GO:0010830 | regulation of myotube differentiation | 0.05569701 | 0.01945885 | 1 |
| BP | GO:0045453 | bone resorption | 0.05569701 | 0.01945885 | 1 |
| BP | GO:0048857 | neural nucleus development | 0.05569701 | 0.01945885 | 1 |
| BP | GO:1902686 | mitochondrial outer membrane permeabilization involved in programmed cell death | 0.05569701 | 0.01945885 | 1 |
| BP | GO:1905818 | regulation of chromosome separation | 0.05569701 | 0.01945885 | 1 |
| BP | GO:0046686 | response to cadmium ion | 0.05647849 | 0.01973187 | 1 |
| BP | GO:0035794 | positive regulation of mitochondrial membrane permeability | 0.05684255 | 0.01985906 | 1 |
| BP | GO:0045600 | positive regulation of fat cell differentiation | 0.05684255 | 0.01985906 | 1 |
| BP | GO:0045669 | positive regulation of osteoblast differentiation | 0.05684255 | 0.01985906 | 1 |
| BP | GO:0046782 | regulation of viral transcription | 0.05684255 | 0.01985906 | 1 |
| BP | GO:0050922 | negative regulation of chemotaxis | 0.05684255 | 0.01985906 | 1 |
| BP | GO:0051148 | negative regulation of muscle cell differentiation | 0.05761266 | 0.02012812 | 1 |
| BP | GO:1902108 | regulation of mitochondrial membrane permeability involved in apoptotic process | 0.05827457 | 0.02035937 | 1 |
| BP | GO:1905710 | positive regulation of membrane permeability | 0.05827457 | 0.02035937 | 1 |
| BP | GO:0042108 | positive regulation of cytokine biosynthetic process | 0.05903712 | 0.02062578 | 1 |
| BP | GO:0002292 | T cell differentiation involved in immune response | 0.05937092 | 0.0207424 | 1 |
| BP | GO:0019915 | lipid storage | 0.05937092 | 0.0207424 | 1 |
| BP | GO:0033047 | regulation of mitotic sister chromatid segregation | 0.05937092 | 0.0207424 | 1 |
| BP | GO:0035914 | skeletal muscle cell differentiation | 0.05937092 | 0.0207424 | 1 |
| BP | GO:0071230 | cellular response to amino acid stimulus | 0.05937092 | 0.0207424 | 1 |
| BP | GO:0042698 | ovulation cycle | 0.06012256 | 0.021005 | 1 |
| BP | GO:0002709 | regulation of T cell mediated immunity | 0.06076339 | 0.02122888 | 1 |
| BP | GO:0060395 | SMAD protein signal transduction | 0.06076339 | 0.02122888 | 1 |
| BP | GO:0042531 | positive regulation of tyrosine phosphorylation of STAT protein | 0.06139929 | 0.02145105 | 1 |
| BP | GO:0071479 | cellular response to ionizing radiation | 0.06139929 | 0.02145105 | 1 |
| BP | GO:0002312 | B cell activation involved in immune response | 0.06287672 | 0.02196722 | 1 |
| BP | GO:0045685 | regulation of glial cell differentiation | 0.06287672 | 0.02196722 | 1 |
| BP | GO:0014015 | positive regulation of gliogenesis | 0.06316766 | 0.02206886 | 1 |
| BP | GO:0031670 | cellular response to nutrient | 0.06316766 | 0.02206886 | 1 |
| BP | GO:0032507 | maintenance of protein location in cell | 0.06316766 | 0.02206886 | 1 |
| BP | GO:0043367 | CD4-positive, alpha-beta T cell differentiation | 0.06316766 | 0.02206886 | 1 |
| BP | GO:1903036 | positive regulation of response to wounding | 0.06316766 | 0.02206886 | 1 |
| BP | GO:0001960 | negative regulation of cytokine-mediated signaling pathway | 0.06389458 | 0.02232283 | 1 |
| BP | GO:0046902 | regulation of mitochondrial membrane permeability | 0.06461857 | 0.02257577 | 1 |
| BP | GO:0048678 | response to axon injury | 0.06522661 | 0.0227882 | 1 |
| BP | GO:0051851 | modification by host of symbiont morphology or physiology | 0.06522661 | 0.0227882 | 1 |
| BP | GO:0030433 | ubiquitin-dependent ERAD pathway | 0.06571674 | 0.02295944 | 1 |
| BP | GO:0043407 | negative regulation of MAP kinase activity | 0.06571674 | 0.02295944 | 1 |
| BP | GO:0072347 | response to anesthetic | 0.06571674 | 0.02295944 | 1 |
| BP | GO:0003151 | outflow tract morphogenesis | 0.06642894 | 0.02320826 | 1 |
| BP | GO:0022617 | extracellular matrix disassembly | 0.06702334 | 0.02341592 | 1 |
| BP | GO:0033045 | regulation of sister chromatid segregation | 0.06702334 | 0.02341592 | 1 |
| BP | GO:0048708 | astrocyte differentiation | 0.06749809 | 0.02358179 | 1 |
| BP | GO:0060761 | negative regulation of response to cytokine stimulus | 0.06749809 | 0.02358179 | 1 |
| BP | GO:0110110 | positive regulation of animal organ morphogenesis | 0.06749809 | 0.02358179 | 1 |
| BP | GO:0032642 | regulation of chemokine production | 0.06808302 | 0.02378614 | 1 |
| BP | GO:0051702 | interaction with symbiont | 0.06808302 | 0.02378614 | 1 |
| BP | GO:0042509 | regulation of tyrosine phosphorylation of STAT protein | 0.06854761 | 0.02394846 | 1 |
| BP | GO:0045582 | positive regulation of T cell differentiation | 0.06854761 | 0.02394846 | 1 |
| BP | GO:0048145 | regulation of fibroblast proliferation | 0.06854761 | 0.02394846 | 1 |
| BP | GO:0002718 | regulation of cytokine production involved in immune response | 0.0691233 | 0.02414959 | 1 |
| BP | GO:0048144 | fibroblast proliferation | 0.0691233 | 0.02414959 | 1 |
| BP | GO:1904705 | regulation of vascular smooth muscle cell proliferation | 0.06969474 | 0.02434923 | 1 |
| BP | GO:1990874 | vascular smooth muscle cell proliferation | 0.06969474 | 0.02434923 | 1 |
| BP | GO:0001776 | leukocyte homeostasis | 0.07002776 | 0.02446558 | 1 |
| BP | GO:0007260 | tyrosine phosphorylation of STAT protein | 0.07002776 | 0.02446558 | 1 |
| BP | GO:0008625 | extrinsic apoptotic signaling pathway via death domain receptors | 0.07002776 | 0.02446558 | 1 |
| BP | GO:0090559 | regulation of membrane permeability | 0.07002776 | 0.02446558 | 1 |
| BP | GO:0002690 | positive regulation of leukocyte chemotaxis | 0.07070718 | 0.02470294 | 1 |
| BP | GO:0034103 | regulation of tissue remodeling | 0.07102999 | 0.02481572 | 1 |
| BP | GO:0050829 | defense response to Gram-negative bacterium | 0.07102999 | 0.02481572 | 1 |
| BP | GO:0097194 | execution phase of apoptosis | 0.07102999 | 0.02481572 | 1 |
| BP | GO:1904035 | regulation of epithelial cell apoptotic process | 0.07102999 | 0.02481572 | 1 |
| BP | GO:0006094 | gluconeogenesis | 0.07146553 | 0.02496789 | 1 |
| BP | GO:0032602 | chemokine production | 0.07146553 | 0.02496789 | 1 |
| BP | GO:0046427 | positive regulation of JAK-STAT cascade | 0.07146553 | 0.02496789 | 1 |
| BP | GO:0010717 | regulation of epithelial to mesenchymal transition | 0.07166184 | 0.02503647 | 1 |
| BP | GO:0030901 | midbrain development | 0.07166184 | 0.02503647 | 1 |
| BP | GO:0045778 | positive regulation of ossification | 0.07166184 | 0.02503647 | 1 |
| BP | GO:0046849 | bone remodeling | 0.07166184 | 0.02503647 | 1 |
| BP | GO:0051304 | chromosome separation | 0.07166184 | 0.02503647 | 1 |
| BP | GO:0051899 | membrane depolarization | 0.0723227 | 0.02526736 | 1 |
| BP | GO:0019319 | hexose biosynthetic process | 0.07274447 | 0.02541471 | 1 |
| BP | GO:0035710 | CD4-positive, alpha-beta T cell activation | 0.07274447 | 0.02541471 | 1 |
| BP | GO:1904894 | positive regulation of STAT cascade | 0.07274447 | 0.02541471 | 1 |
| BP | GO:0007589 | body fluid secretion | 0.07339856 | 0.02564323 | 1 |
| BP | GO:0045621 | positive regulation of lymphocyte differentiation | 0.07393073 | 0.02582915 | 1 |
| BP | GO:1901216 | positive regulation of neuron death | 0.07393073 | 0.02582915 | 1 |
| BP | GO:0042102 | positive regulation of T cell proliferation | 0.0744591 | 0.02601375 | 1 |
| BP | GO:0051651 | maintenance of location in cell | 0.0744591 | 0.02601375 | 1 |
| BP | GO:0008585 | female gonad development | 0.07474415 | 0.02611334 | 1 |
| BP | GO:0042632 | cholesterol homeostasis | 0.07474415 | 0.02611334 | 1 |
| BP | GO:1990823 | response to leukemia inhibitory factor | 0.07474415 | 0.02611334 | 1 |
| BP | GO:1990830 | cellular response to leukemia inhibitory factor | 0.07474415 | 0.02611334 | 1 |
| BP | GO:0055092 | sterol homeostasis | 0.07514449 | 0.02625321 | 1 |
| BP | GO:1990868 | response to chemokine | 0.07514449 | 0.02625321 | 1 |
| BP | GO:1990869 | cellular response to chemokine | 0.07514449 | 0.02625321 | 1 |
| BP | GO:0006626 | protein targeting to mitochondrion | 0.07566038 | 0.02643344 | 1 |
| BP | GO:0046364 | monosaccharide biosynthetic process | 0.07566038 | 0.02643344 | 1 |
| BP | GO:0048525 | negative regulation of viral process | 0.07617265 | 0.02661241 | 1 |
| BP | GO:0070301 | cellular response to hydrogen peroxide | 0.07617265 | 0.02661241 | 1 |
| BP | GO:0002824 | positive regulation of adaptive immune response based on somatic recombination of immune receptors built from immunoglobulin superfamily domains | 0.07656076 | 0.02674801 | 1 |
| BP | GO:0036503 | ERAD pathway | 0.07656076 | 0.02674801 | 1 |
| BP | GO:0070498 | interleukin-1-mediated signaling pathway | 0.07656076 | 0.02674801 | 1 |
| BP | GO:0046545 | development of primary female sexual characteristics | 0.07682464 | 0.0268402 | 1 |
| BP | GO:0046632 | alpha-beta T cell differentiation | 0.07682464 | 0.0268402 | 1 |
| BP | GO:0050830 | defense response to Gram-positive bacterium | 0.07682464 | 0.0268402 | 1 |
| BP | GO:1990542 | mitochondrial transmembrane transport | 0.07682464 | 0.0268402 | 1 |
| BP | GO:0002367 | cytokine production involved in immune response | 0.07720477 | 0.026973 | 1 |
| BP | GO:0021549 | cerebellum development | 0.07720477 | 0.026973 | 1 |
| BP | GO:0035335 | peptidyl-tyrosine dephosphorylation | 0.07720477 | 0.026973 | 1 |
| BP | GO:0032091 | negative regulation of protein binding | 0.07770128 | 0.02714647 | 1 |
| BP | GO:0051983 | regulation of chromosome segregation | 0.07770128 | 0.02714647 | 1 |
| BP | GO:0071887 | leukocyte apoptotic process | 0.07831542 | 0.02736103 | 1 |
| BP | GO:0002708 | positive regulation of lymphocyte mediated immunity | 0.07856303 | 0.02744754 | 1 |
| BP | GO:0002821 | positive regulation of adaptive immune response | 0.07856303 | 0.02744754 | 1 |
| BP | GO:0033138 | positive regulation of peptidyl-serine phosphorylation | 0.07856303 | 0.02744754 | 1 |
| BP | GO:0045185 | maintenance of protein location | 0.07856303 | 0.02744754 | 1 |
| BP | GO:0002286 | T cell activation involved in immune response | 0.07892794 | 0.02757503 | 1 |
| BP | GO:0002456 | T cell mediated immunity | 0.07892794 | 0.02757503 | 1 |
| BP | GO:0007041 | lysosomal transport | 0.07892794 | 0.02757503 | 1 |
| BP | GO:0048524 | positive regulation of viral process | 0.07953163 | 0.02778594 | 1 |
| BP | GO:0006275 | regulation of DNA replication | 0.0798892 | 0.02791086 | 1 |
| BP | GO:0032526 | response to retinoic acid | 0.0798892 | 0.02791086 | 1 |
| BP | GO:2000278 | regulation of DNA biosynthetic process | 0.0798892 | 0.02791086 | 1 |
| BP | GO:0022037 | metencephalon development | 0.08180026 | 0.02857853 | 1 |
| BP | GO:1904019 | epithelial cell apoptotic process | 0.08180026 | 0.02857853 | 1 |
| BP | GO:0001938 | positive regulation of endothelial cell proliferation | 0.08201956 | 0.02865515 | 1 |
| BP | GO:0014902 | myotube differentiation | 0.08201956 | 0.02865515 | 1 |
| BP | GO:0043279 | response to alkaloid | 0.08201956 | 0.02865515 | 1 |
| BP | GO:0051817 | modification of morphology or physiology of other organism involved in symbiotic interaction | 0.08201956 | 0.02865515 | 1 |
| BP | GO:0043200 | response to amino acid | 0.08260744 | 0.02886053 | 1 |
| BP | GO:0042035 | regulation of cytokine biosynthetic process | 0.0831932 | 0.02906518 | 1 |
| BP | GO:0032963 | collagen metabolic process | 0.08365125 | 0.02922521 | 1 |
| BP | GO:0046660 | female sex differentiation | 0.08365125 | 0.02922521 | 1 |
| BP | GO:0021782 | glial cell development | 0.08423214 | 0.02942815 | 1 |
| BP | GO:0002761 | regulation of myeloid leukocyte differentiation | 0.08455815 | 0.02954205 | 1 |
| BP | GO:0031398 | positive regulation of protein ubiquitination | 0.08455815 | 0.02954205 | 1 |
| BP | GO:0051153 | regulation of striated muscle cell differentiation | 0.08455815 | 0.02954205 | 1 |
| BP | GO:0045047 | protein targeting to ER | 0.08513355 | 0.02974308 | 1 |
| BP | GO:0022612 | gland morphogenesis | 0.0864064 | 0.03018777 | 1 |
| BP | GO:0072599 | establishment of protein localization to endoplasmic reticulum | 0.08767412 | 0.03063068 | 1 |
| BP | GO:0042089 | cytokine biosynthetic process | 0.08824065 | 0.0308286 | 1 |
| BP | GO:0014013 | regulation of gliogenesis | 0.0885432 | 0.03093431 | 1 |
| BP | GO:0042107 | cytokine metabolic process | 0.0885432 | 0.03093431 | 1 |
| BP | GO:0051101 | regulation of DNA binding | 0.0885432 | 0.03093431 | 1 |
| BP | GO:0045667 | regulation of osteoblast differentiation | 0.08979575 | 0.03137191 | 1 |
| BP | GO:0035270 | endocrine system development | 0.09022097 | 0.03152047 | 1 |
| BP | GO:0045727 | positive regulation of translation | 0.09022097 | 0.03152047 | 1 |
| BP | GO:0002687 | positive regulation of leukocyte migration | 0.09077628 | 0.03171448 | 1 |
| BP | GO:0050671 | positive regulation of lymphocyte proliferation | 0.09201556 | 0.03214744 | 1 |
| BP | GO:0030183 | B cell differentiation | 0.09243049 | 0.03229241 | 1 |
| BP | GO:0032946 | positive regulation of mononuclear cell proliferation | 0.09243049 | 0.03229241 | 1 |
| BP | GO:0002705 | positive regulation of leukocyte mediated immunity | 0.09352361 | 0.03267431 | 1 |
| BP | GO:0071333 | cellular response to glucose stimulus | 0.09352361 | 0.03267431 | 1 |
| BP | GO:0007006 | mitochondrial membrane organization | 0.09392745 | 0.0328154 | 1 |
| BP | GO:0032355 | response to estradiol | 0.09392745 | 0.0328154 | 1 |
| BP | GO:0043484 | regulation of RNA splicing | 0.09392745 | 0.0328154 | 1 |
| BP | GO:0050921 | positive regulation of chemotaxis | 0.09392745 | 0.0328154 | 1 |
| BP | GO:0060359 | response to ammonium ion | 0.09392745 | 0.0328154 | 1 |
| BP | GO:0071331 | cellular response to hexose stimulus | 0.09392745 | 0.0328154 | 1 |
| BP | GO:1903322 | positive regulation of protein modification by small protein conjugation or removal | 0.09392745 | 0.0328154 | 1 |
| BP | GO:0071326 | cellular response to monosaccharide stimulus | 0.09446443 | 0.033003 | 1 |
| BP | GO:0046425 | regulation of JAK-STAT cascade | 0.09486323 | 0.03314234 | 1 |
| BP | GO:0072655 | establishment of protein localization to mitochondrion | 0.09486323 | 0.03314234 | 1 |
| BP | GO:0046631 | alpha-beta T cell activation | 0.09539591 | 0.03332844 | 1 |
| BP | GO:0033135 | regulation of peptidyl-serine phosphorylation | 0.09551679 | 0.03337067 | 1 |
| BP | GO:0045580 | regulation of T cell differentiation | 0.09551679 | 0.03337067 | 1 |
| BP | GO:0050715 | positive regulation of cytokine secretion | 0.09551679 | 0.03337067 | 1 |
| BP | GO:0070665 | positive regulation of leukocyte proliferation | 0.09551679 | 0.03337067 | 1 |
| BP | GO:0001837 | epithelial to mesenchymal transition | 0.0960441 | 0.0335549 | 1 |
| BP | GO:0070585 | protein localization to mitochondrion | 0.0965696 | 0.03373849 | 1 |
| BP | GO:0007034 | vacuolar transport | 0.09709328 | 0.03392145 | 1 |
| BP | GO:0030879 | mammary gland development | 0.09761515 | 0.03410377 | 1 |
| BP | GO:0071322 | cellular response to carbohydrate stimulus | 0.09813523 | 0.03428547 | 1 |
| BP | GO:0002822 | regulation of adaptive immune response based on somatic recombination of immune receptors built from immunoglobulin superfamily domains | 0.09837563 | 0.03436946 | 1 |
| BP | GO:0007612 | learning | 0.09837563 | 0.03436946 | 1 |
| BP | GO:0071901 | negative regulation of protein serine/threonine kinase activity | 0.09837563 | 0.03436946 | 1 |
| BP | GO:0055123 | digestive system development | 0.09847557 | 0.03440438 | 1 |
| BP | GO:1904892 | regulation of STAT cascade | 0.09847557 | 0.03440438 | 1 |
| BP | GO:2000241 | regulation of reproductive process | 0.09847557 | 0.03440438 | 1 |
| BP | GO:2001251 | negative regulation of chromosome organization | 0.09847557 | 0.03440438 | 1 |
| BP | GO:0010212 | response to ionizing radiation | 0.09871156 | 0.03448683 | 1 |
| BP | GO:0043524 | negative regulation of neuron apoptotic process | 0.09871156 | 0.03448683 | 1 |
| BP | GO:0070972 | protein localization to endoplasmic reticulum | 0.09871156 | 0.03448683 | 1 |
| BP | GO:0061041 | regulation of wound healing | 0.09922089 | 0.03466477 | 1 |
| BP | GO:0002706 | regulation of lymphocyte mediated immunity | 0.09972849 | 0.03484211 | 1 |
| CC | GO:0008180 | COP9 signalosome | 7.4088E-08 | 3.4889E-08 | 4 |
| CC | GO:1904813 | ficolin-1-rich granule lumen | 0.00044545 | 0.00020977 | 3 |
| CC | GO:0005814 | centriole | 0.00044545 | 0.00020977 | 3 |
| CC | GO:0072562 | blood microparticle | 0.00044545 | 0.00020977 | 3 |
| CC | GO:0044450 | microtubule organizing center part | 0.00058844 | 0.0002771 | 3 |
| CC | GO:0101002 | ficolin-1-rich granule | 0.00058844 | 0.0002771 | 3 |
| CC | GO:0016235 | aggresome | 0.00112238 | 0.00052854 | 2 |
| CC | GO:0016234 | inclusion body | 0.00336597 | 0.00158508 | 2 |
| CC | GO:0005925 | focal adhesion | 0.00336597 | 0.00158508 | 3 |
| CC | GO:0005924 | cell-substrate adherens junction | 0.00336597 | 0.00158508 | 3 |
| CC | GO:0030055 | cell-substrate junction | 0.00336597 | 0.00158508 | 3 |
| CC | GO:0030176 | integral component of endoplasmic reticulum membrane | 0.00787114 | 0.00370663 | 2 |
| CC | GO:0031227 | intrinsic component of endoplasmic reticulum membrane | 0.00804663 | 0.00378927 | 2 |
| CC | GO:0034663 | endoplasmic reticulum chaperone complex | 0.01510832 | 0.00711472 | 1 |
| CC | GO:0005744 | TIM23 mitochondrial import inner membrane translocase complex | 0.01793458 | 0.00844565 | 1 |
| CC | GO:0005788 | endoplasmic reticulum lumen | 0.02407865 | 0.01133898 | 2 |
| CC | GO:0005719 | nuclear euchromatin | 0.0337864 | 0.01591049 | 1 |
| CC | GO:0016607 | nuclear speck | 0.03422936 | 0.01611909 | 2 |
| CC | GO:0005790 | smooth endoplasmic reticulum | 0.03422936 | 0.01611909 | 1 |
| CC | GO:0000791 | euchromatin | 0.03631041 | 0.01709909 | 1 |
| CC | GO:0017053 | transcriptional repressor complex | 0.07564651 | 0.03562301 | 1 |
| CC | GO:0042470 | melanosome | 0.08672307 | 0.04083912 | 1 |
| CC | GO:0048770 | pigment granule | 0.08672307 | 0.04083912 | 1 |
| CC | GO:0005793 | endoplasmic reticulum-Golgi intermediate compartment | 0.09834289 | 0.04631105 | 1 |
| KEGG | hsa05162 | Measles | 6.4078E-06 | 3.5974E-06 | 5 |
| KEGG | hsa05134 | Legionellosis | 6.4078E-06 | 3.5974E-06 | 4 |
| KEGG | hsa04141 | Protein processing in endoplasmic reticulum | 6.4078E-06 | 3.5974E-06 | 5 |
| KEGG | hsa04612 | Antigen processing and presentation | 1.2317E-05 | 6.9146E-06 | 4 |
| KEGG | hsa05020 | Prion disease | 3.9473E-05 | 2.216E-05 | 5 |
| KEGG | hsa04010 | MAPK signaling pathway | 4.7455E-05 | 2.6642E-05 | 5 |
| KEGG | hsa04915 | Estrogen signaling pathway | 6.967E-05 | 3.9113E-05 | 4 |
| KEGG | hsa04213 | Longevity regulating pathway - multiple species | 0.00026415 | 0.0001483 | 3 |
| KEGG | hsa05145 | Toxoplasmosis | 0.00138235 | 0.00077606 | 3 |
| KEGG | hsa04932 | Non-alcoholic fatty liver disease | 0.00274064 | 0.0015386 | 3 |
| KEGG | hsa03040 | Spliceosome | 0.00274064 | 0.0015386 | 3 |
| KEGG | hsa05012 | Parkinson disease | 0.01036108 | 0.00581675 | 3 |
| KEGG | hsa04144 | Endocytosis | 0.01036108 | 0.00581675 | 3 |
| KEGG | hsa05321 | Inflammatory bowel disease | 0.01112899 | 0.00624786 | 2 |
| KEGG | hsa05133 | Pertussis | 0.01415474 | 0.00794652 | 2 |
| KEGG | hsa05323 | Rheumatoid arthritis | 0.0187382 | 0.01051969 | 2 |
| KEGG | hsa04657 | IL-17 signaling pathway | 0.0187382 | 0.01051969 | 2 |
| KEGG | hsa04933 | AGE-RAGE signaling pathway in diabetic complications | 0.0187382 | 0.01051969 | 2 |
| KEGG | hsa05142 | Chagas disease | 0.0187382 | 0.01051969 | 2 |
| KEGG | hsa04620 | Toll-like receptor signaling pathway | 0.0187382 | 0.01051969 | 2 |
| KEGG | hsa04625 | C-type lectin receptor signaling pathway | 0.0187382 | 0.01051969 | 2 |
| KEGG | hsa04659 | Th17 cell differentiation | 0.01891019 | 0.01061625 | 2 |
| KEGG | hsa04668 | TNF signaling pathway | 0.0197771 | 0.01110293 | 2 |
| KEGG | hsa05135 | Yersinia infection | 0.02769945 | 0.01555057 | 2 |
| KEGG | hsa05418 | Fluid shear stress and atherosclerosis | 0.02769945 | 0.01555057 | 2 |
| KEGG | hsa05022 | Pathways of neurodegeneration - multiple diseases | 0.03137537 | 0.01761424 | 3 |
| KEGG | hsa05161 | Hepatitis B | 0.03447647 | 0.01935521 | 2 |
| KEGG | hsa04621 | NOD-like receptor signaling pathway | 0.04113668 | 0.02309428 | 2 |
| KEGG | hsa05167 | Kaposi sarcoma-associated herpesvirus infection | 0.04490557 | 0.02521014 | 2 |
| KEGG | hsa05130 | Pathogenic Escherichia coli infection | 0.04514139 | 0.02534254 | 2 |
| KEGG | hsa05169 | Epstein-Barr virus infection | 0.04582235 | 0.02572483 | 2 |
| KEGG | hsa05166 | Human T-cell leukemia virus 1 infection | 0.05175609 | 0.02905605 | 2 |
| KEGG | hsa03060 | Protein export | 0.06154753 | 0.034553 | 1 |
| KEGG | hsa05132 | Salmonella infection | 0.0620702 | 0.03484643 | 2 |
| KEGG | hsa01523 | Antifolate resistance | 0.07794469 | 0.04375842 | 1 |
| KEGG | hsa05143 | African trypanosomiasis | 0.09021209 | 0.05064538 | 1 |
| KEGG | hsa05332 | Graft-versus-host disease | 0.09942004 | 0.05581476 | 1 |
| MF | GO:0044183 | protein binding involved in protein folding | 1.9861E-08 | 6.8347E-09 | 4 |
| MF | GO:0051787 | misfolded protein binding | 1.9861E-08 | 6.8347E-09 | 4 |
| MF | GO:0051082 | unfolded protein binding | 8.7282E-08 | 3.0036E-08 | 5 |
| MF | GO:0031072 | heat shock protein binding | 5.1429E-06 | 1.7698E-06 | 4 |
| MF | GO:0042623 | ATPase activity, coupled | 0.00011894 | 4.093E-05 | 4 |
| MF | GO:0031625 | ubiquitin protein ligase binding | 0.00011894 | 4.093E-05 | 4 |
| MF | GO:0044389 | ubiquitin-like protein ligase binding | 0.00012923 | 4.4472E-05 | 4 |
| MF | GO:0016887 | ATPase activity | 0.00026861 | 9.2435E-05 | 4 |
| MF | GO:0001618 | virus receptor activity | 0.00394974 | 0.00135922 | 2 |
| MF | GO:0104005 | hijacked molecular function | 0.00394974 | 0.00135922 | 2 |
| MF | GO:0051087 | chaperone binding | 0.00679009 | 0.00233667 | 2 |
| MF | GO:0047485 | protein N-terminus binding | 0.0067911 | 0.00233702 | 2 |
| MF | GO:0042826 | histone deacetylase binding | 0.0067911 | 0.00233702 | 2 |
| MF | GO:0001158 | enhancer sequence-specific DNA binding | 0.0072347 | 0.00248968 | 2 |
| MF | GO:0035326 | enhancer binding | 0.0084066 | 0.00289296 | 2 |
| MF | GO:0017017 | MAP kinase tyrosine/serine/threonine phosphatase activity | 0.02277843 | 0.00783873 | 1 |
| MF | GO:0003714 | transcription corepressor activity | 0.02277843 | 0.00783873 | 2 |
| MF | GO:0035497 | cAMP response element binding | 0.02277843 | 0.00783873 | 1 |
| MF | GO:0033549 | MAP kinase phosphatase activity | 0.02311508 | 0.00795458 | 1 |
| MF | GO:0071837 | HMG box domain binding | 0.02341733 | 0.0080586 | 1 |
| MF | GO:0001664 | G protein-coupled receptor binding | 0.02556043 | 0.0087961 | 2 |
| MF | GO:0070412 | R-SMAD binding | 0.03054781 | 0.0105124 | 1 |
| MF | GO:0045296 | cadherin binding | 0.0316305 | 0.01088499 | 2 |
| MF | GO:0051019 | mitogen-activated protein kinase binding | 0.0316305 | 0.01088499 | 1 |
| MF | GO:0060589 | nucleoside-triphosphatase regulator activity | 0.03180895 | 0.0109464 | 2 |
| MF | GO:0097718 | disordered domain specific binding | 0.03699239 | 0.01273017 | 1 |
| MF | GO:0060590 | ATPase regulator activity | 0.04310187 | 0.01483263 | 1 |
| MF | GO:0001228 | DNA-binding transcription activator activity, RNA polymerase II-specific | 0.04497421 | 0.01547695 | 2 |
| MF | GO:0008138 | protein tyrosine/serine/threonine phosphatase activity | 0.04508823 | 0.01551619 | 1 |
| MF | GO:0001102 | RNA polymerase II activating transcription factor binding | 0.05122969 | 0.01762965 | 1 |
| MF | GO:0050839 | cell adhesion molecule binding | 0.0515566 | 0.01774215 | 2 |
| MF | GO:0043022 | ribosome binding | 0.05160019 | 0.01775715 | 1 |
| MF | GO:0004722 | protein serine/threonine phosphatase activity | 0.06639478 | 0.0228484 | 1 |
| MF | GO:0046332 | SMAD binding | 0.06776495 | 0.02331992 | 1 |
| MF | GO:0033613 | activating transcription factor binding | 0.06985457 | 0.02403902 | 1 |
| MF | GO:0000980 | RNA polymerase II distal enhancer sequence-specific DNA binding | 0.07744486 | 0.02665106 | 1 |
| MF | GO:0004725 | protein tyrosine phosphatase activity | 0.07744486 | 0.02665106 | 1 |
| MF | GO:0043021 | ribonucleoprotein complex binding | 0.09722791 | 0.033459 | 1 |
| MF | GO:0070851 | growth factor receptor binding | 0.09722791 | 0.033459 | 1 |
| MF | GO:0019838 | growth factor binding | 0.09722791 | 0.033459 | 1 |
